# UV-Induced Photodegradation of 2-Aminothiazole-4-Carboxylic Acid: Identification of New Carbodiimide Molecules

**DOI:** 10.3390/molecules30183713

**Published:** 2025-09-12

**Authors:** Daria Bumażnik, Magdalena Sałdyka

**Affiliations:** Faculty of Chemistry, University of Wrocław, F. Joliot-Curie 14, 50-383 Wrocław, Poland; daria.bumaznik@uwr.edu.pl

**Keywords:** thiazole, carboxylic acid, carbodiimide, matrix isolation, FTIR, photochemistry, DFT, B3LYP-D3, molecular complex

## Abstract

The UV-induced photolysis of 2-aminothiazole-4-carboxylic acid (ACA), a biologically active molecule, was studied using the infrared matrix isolation method. As the first step of photolysis, a decarboxylation reaction occurred. Subsequently, two main photolysis pathways of 2-aminothiazole were observed, during which a number of new molecules, including potential prebiotic carbodiimides or molecular complexes, were identified. The CS–CN bond cleavage path produced *N*-(1-sulfanylethen-2-yl)carbodiimide (fp1), *N*-(thiiran-2-yl)carbodiimide (fp3), *N*-(1-thioethan-2-yl)carbodiimide (fp2), *N*-(1-thioethan-1-yl)carbodiimide (fp4) and *N*-(1-thioethan-2-yl)cyanamide (fp33), which were identified for the first time. In this channel, additional disruption of the N–C bond produced cyanamide (fp27) and thiirene (fp28) and subsequent photoreactions generated carbodiimide (fp29) or ethynethiol (fp30). The CS–CC bond cleavage path occurred simultaneously and produced several new molecules: *N*’-ethynylcarbamimidothioic acid (fp14), *N*-ethynylcarbamimidothioic acid (fp17), *N*-ethenylidenecarbamimidothioic acid (fp18) and *N*-ethenylidenethiourea (fp15). In this channel, additional disruption of the N–C bond produced acetylene (fp23) and *N*-thiolcarbodiimide (fp26). Among the small molecules, *N*-thiolcarbodiimide and thiirene, as well as all molecular complexes, were observed for the first time.

## 1. Introduction

Thiazole derivatives are of interest in many fields such as pharmacology, biochemistry, biology, chemistry and genetics [1]. Many S-heterocycles, including thiazole derivatives, exhibit a wide spectrum of biological activity; therefore, they have been used in the synthesis of drugs and medically important compounds [2,3,4]. The literature indicates that some of the 2-aminothiazole derivatives exhibit psychotropic, antiallergic, antifungal, anticancer, antiviral and antibacterial properties [5,6,7]. Both mono- and bicyclic 2-aminothiazole derivatives are being studied for their use in the treatment of various neurodegenerative diseases [8,9]. Moreover, thiazole carboxylate derivatives with both anticancer and antibacterial properties are being investigated for the possibility of monotherapy in the treatment of bacterial infections associated with cancer [10].

Taking the above into account, the examination of photochemical reactions of heterocycles and the determination of their decay products is important in the context of analysing the effect of a drug containing thiazole as an active ingredient on the human body. In addition, the fact that the photochemistry of heterocyclic compounds with various functional groups is extremely complex, this type of research may contribute to finding new practical applications of thiazole or its derivatives. Photochemistry of some five-membered ring containing two heteroatoms has been investigated but the complete reaction pathways have not been established yet [11]. In flash photolysis of thiazole in the gas phase, initially the highly excited NCS molecule was detected, which acted as a precursor to the rotationally and vibrationally excited CN radical [12]. VUV (vacuum ultraviolet) photodissociation of thiazole studied by TOF-MS (time-of-flight mass spectrometry) and photoelectron photoion coincidence spectroscopy produced several ionic species, like C_2_H_2_S^+^, CHS^+^, CH_2_N^+^ and S^+^ [13]. Noteworthy is the work on the spectroscopic and photochemical analysis of non-substituted thiazole isolated in an argon matrix, especially the detailed photolysis of the compound under the influence of UV (ultraviolet) radiation [14]. The preferred paths of photolysis of thiazole were studied, taking place mainly by breaking the CS–CN bond with the accompanying hydrogen migration, and the resulting photoproducts, among which a group of isocyano compounds should be highlighted, were determined. On the other hand, in our group, we recently conducted an analysis of the photolytic transformations of the irradiated thiazole derivative, namely 2-amino-4-methylthiazole, in low-temperature matrices, which occurred in a similar way to the previously discussed unsubstituted thiazole. It was possible to propose the full photolysis path of the compound and indicate several photoproducts and molecular complexes, most of which belonged to the group of carbodiimides [15]. It can be assumed that the difference in the type of photoproducts observed between thiazole and its derivative results from the attachment of the NH_2_ group to the ring: the presence of one nitrogen atom in the precursor molecule produces mainly isocyano molecules upon irradiation, while the presence of two nitrogen atoms generates carbodiimides. In the literature, one can also find photochemical studies on the carboxylic acids of heterocyclic compounds; in particular, photochemistry in low-temperature matrices was investigated for carboxylic acids of pyridine [16], pyrazine [17], indazole [18] or furan [19]. Photo-transformations were also studied for thiazole-2-carboxylic acid in solid argon and nitrogen [20,21] and, interestingly, irradiation of the molecule at λ = 254 nm in an Ar matrix resulted in the loss of CO_2_ and production of 2,3-dihydrothiazole-2-ylidene. A similar decarboxylation path in the photolysis mechanism (production of CO_2_ + appropriate co-product) was observed for other heterocyclic carboxylic acids: 3-aminopyrazine-2-carboxylic acid [17], indazole-3-carboxylic acid [18] or 2-furoic acid [19]. Our photochemical study on both COOH- and NH_2_-substituted thiazole (ACA) indicates that decarboxylation occurs first, generating 2-aminothiazole in situ, which then undergoes complex ring-opening reactions. It can be stated that such sequential photochemistry can be considered a key entry point into the chemistry of new heterocyclic carboxylic acids.

**Figure 1:**
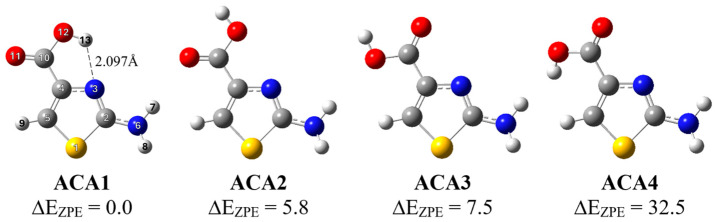
The structure of ACA isomers with the adopted atom numbering; relative energy (ΔE_ZPE_) values (in kJ mol^−1^) are given.

We have recently studied the structure, spectroscopic properties and photo-induced conformational changes of 2-aminothiazole-4-carboxylic acid (ACA) isolated in argon and nitrogen matrices [22]. The three most stable isomers of the studied compound were identified experimentally with the gas phase relative abundance values of ACA1, ACA2 and ACA3 of 69.5, 18.2 and 12.2%, respectively. UV irradiation induced forward ACA2 → ACA1 (λ = 296 nm) and reverse ACA1 → ACA2 (λ = 285 nm) photoisomerization processes that involve the COOH group of the molecule as well as formation/breaking of an intramolecular hydrogen bond between the N atom in the thiazole ring and the OH group of the COOH group. The same photoisomerization processes were observed during selective irradiation using NIR (near-infrared) light at 1434 nm for forward and at 1456 nm for reverse reactions, respectively. In the present paper, we present a combined spectroscopic and theoretical study of photodissociation of ACA in low-temperature matrices and the aim of the work was to investigate the photolysis paths of this thiazole derivative under the influence of UV radiation. Results from our previous work on conformational photo-transformations together with the determination of photolysis pathways will allow for the full characterization of the photochemical behavior of the ACA molecule. The structures of the most stable ACA isomers are presented in Figure 1.

## 2. Results and Discussion

### 2.1. UV-Induced Photolysis of ACA

**Figure 2:**
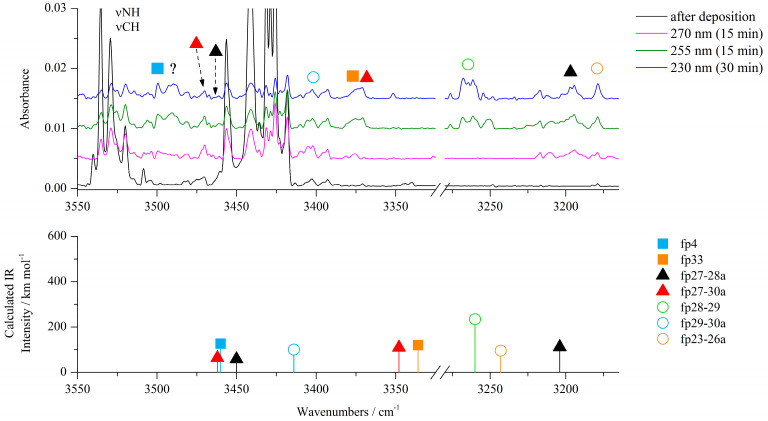
Top: 3550−3160 cm^−1^ region of the ACA/Ar spectra after deposition at 15 K/10 K (black traces) and after irradiation at 270 nm (pink traces), 255 nm (green traces) and 230 nm (blue traces). Bottom: anharmonic B3LYP-D3/6-311++G(3df,3pd) stick spectra of the identified photoproducts.

**Figure 3:**
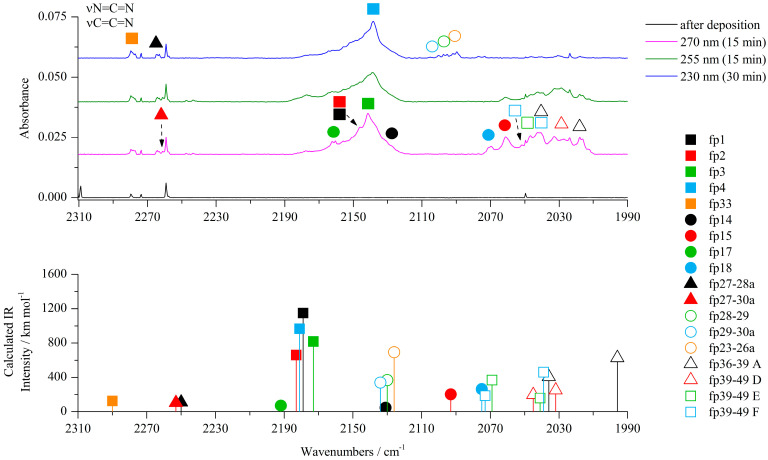
The 2310−1990 cm^−1^ region of the ACA/Ar spectra presented in Figure 1.

**Figure 4:**
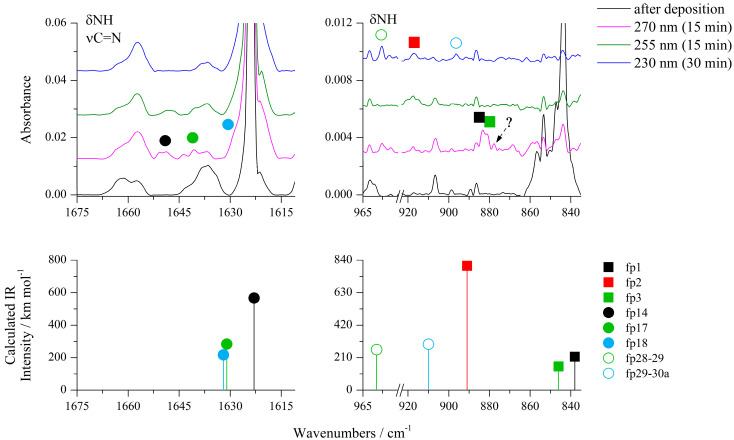
The 1675−1615 cm^−1^ and 965−835 cm^−1^ regions of the ACA/Ar spectra presented in Figure 1.

2-aminothiazole-4-carboxylic acid (ACA) isolated in argon and nitrogen matrices was irradiated with UV light provided by an OPO (optical parametric oscillator) laser system starting at a wavelength of 300 nm and proceeded with gradual decreases in the output wavelength. Infrared spectrum was measured after each step of irradiation in order to control the course of ongoing photochemical reactions. Photoisomerization reactions of ACA were observed at wavelengths of 300–285 nm [22], while the photolysis of the compound began slowly at 285 nm, causing a gradual decrease in the intensity of the bands of all ACA isomers and growth of new bands. The most characteristic changes in the spectra during irradiation at 270, 255 and 230 nm are presented in Figure 2, Figure 3, Figure 4, Figures S6 and S7; Figure S1 shows the behavior of the most representative bands at each step of irradiation in the range 285–230 nm. As shown by careful spectral analysis, new bands that can be observed in Figure 2, Figure 3 and Figure 4 did not originate from isomers of the irradiated compound, but from photodissociation products of ACA.

Also, the gradually increasing intensity of the bands at 2345/2339 cm^−1^, characteristic of asymmetric stretching vibrations of CO_2_ [23] (Figure S2), may also be evidence of the photolysis of the studied compound. The increase in the intensity of CO_2_ bands indicates decarboxylation of ACA; however, comparison of the calculated spectrum with the experimental one excluded the presence of 2-aminothiazole (Table S1), a direct second product of decarboxylation, which indicated further degradation of this molecule in the matrices. Decarboxylation has also been observed as a result of UV irradiations of thiazole-2-carboxylic acid in argon and nitrogen matrices [20,21]. The literature describes the course of photolytic reactions under the influence of UV radiation of two similar thiazole derivatives isolated in low-temperature matrices: unsubstituted thiazole [14] and 2-amino-4-methylthiazole [15] molecules. In both cases, fragmentation of the thiazole ring during irradiation proceeded mainly by breaking one of the C−S bonds. The main photolysis pathway of these compounds is based on the cleavage of the S1–C2 bond, but products related to the cleavage of the S1–C5 bond may also appear in the matrix. These processes can be followed by a C−N bond cleavage leading to the formation of smaller molecules.

Based on all the above considerations and literature reports on photochemical rearrangements of thiazole, thiazole derivatives and five-membered ring heterocycles in general [11,14,15,24,25,26,27], we have proposed detailed photolysis pathways of ACA, which are shown in Figures S3–S5. The structures of possible ACA photoproducts were optimized at the B3LYP-D3/6-311++G(3df,3pd) level of theory; harmonic and anharmonic wavenumbers of selected structures are gathered in Table S1; their geometrical parameters are collected in Table S3 and Cartesian coordinates are presented in Table S4. The comparison of the most intense and characteristic bands for the proposed photoproducts to the bands appearing in the spectra after irradiation allowed for preliminary rejection of the following structures from further considerations: fp5–fp13, fp16, fp19–fp22, fp31, fp32, fp34, fp35, fp41–fp45. The photoproduct bands of ACA were identified and assigned to fp1–fp4, fp14, fp15, fp17, fp18 fp23, fp26–fp30, fp33, fp36, fp39, fp49 structures.

**Scheme 1:**
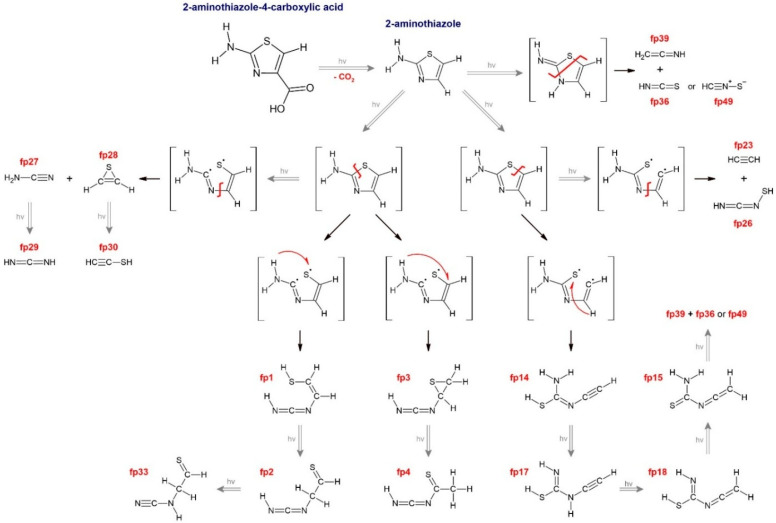
The identified photoreactions of ACA. Description: fp1: *N*-(1-sulfanylethen-2-yl)carbodiimide, fp2: *N*-(1-thioethan-2-yl)carbodiimide, fp3: *N*-(thiiran-2-yl)carbodiimide, fp4: *N*-(1-thioethan-1-yl)carbodiimide, fp14: *N*’-ethynylcarbamimidothioic acid, fp15: *N*-ethenylidenethiourea, fp17: *N*-ethynylcarbamimidothioic acid, fp18: *N*-ethenylidenecarbamimidothioic acid, fp23: acetylene, fp26: *N*-thiolcarbodiimide, fp27: cyanamide, fp28: thiirene, fp29: carbodiimide, fp30: ethynethiol, fp33: *N*-(1-thioethan-2-yl)cyanamide, fp36: isothiocyanic acid, fp39: ketenimine and fp49: thiofulminic acid.

In order to fully interpret the spectra, we considered the possibility of matrix site effects or band overlap between conformers with very similar structures. In the case of ACA photolysis, observed photoproduct bands are weak or broadened, and this did not allow the identification of well-defined site bands. Having obtained the results of anharmonic calculations, we also analyzed the experimental spectrum for the occurrence of combination vibrations and overtones of relatively high intensities (I_theor_ > 100 km mol^−1^). As can be seen in Table S1, only several absorptions from combination vibrations should be expected for all the conformers; comparison of the calculated spectra with the experimental one allowed the identification of only one band due to the δNCN+δCH_2_ vibration for fp15 (Section 2.3.1). Scheme 1 presents the identified photoreaction pathways of ACA.

### 2.2. Ring Opening Reactions by Cleavage of the S1-C2 Bond

#### 2.2.1. Formation of Carbodiimides or Cyanamides Containing Sulfur Atom

The most characteristic regions of the IR spectrum, where new product bands can be observed, are presented in Figure 2, Figure 3, Figure 4, Figures S6 and S7. The most noticeable photoproduct bands appeared in the region 2300–2000 cm^−1^. The broad band at ca. 2141 cm^−1^ in an argon matrix (2140 cm^−1^ in a nitrogen matrix) was identified after 5 min of irradiation at 285 nm and its intensity increased for 65 min of exposure in the range from 285 nm to 270 nm. Further irradiation for 80 min in the wavelength range from 265 nm to 230 nm showed a gradual decrease of 2141 cm^−1^ absorption and appearance of the 2138 cm^−1^ band. According to the literature, the band at ca. 2140 cm^−1^ is characteristic of molecules containing the isocyano, cyano or carbodiimide group [14,26,27,28,29]. As the cleavage of the S1–C2 bond was postulated for the thiazole molecule, a similar reaction can be proposed for ACA as the mechanism of initial photoproduct formation.

The experimental wavenumbers compared with calculated harmonic and anharmonic wavenumbers of the photoproduct molecules observed after photolysis of ACA/Ar(N_2_) matrices together with their optimized structures are gathered in Table 1 and Table 6 placed in Section 2.3.1. All harmonic and anharmonic wavenumbers calculated for the identified photoproducts are collected in Table S1. After the S1–C2 bond cleavage, the hydrogen atom of the NH_2_ group can migrate to S1 giving *N*-(1-sulfanylethen-2-yl)carbodiimide (fp1) as a photoproduct (see Scheme 1). Four conformers of fp1 were optimized with *E* and *Z* conformation around the C=C bond and *syn/anti* orientation of the C–SH group (fp1a–fp1d), with the relative zero point energy difference ΔE_ZPE_ from 5.4 to 10.0 kJ mol^−1^ in terms of the structure with the highest stability (Figure S8). The obtained calculated spectral pattern of fp1a–fp1d did not allow for unambiguous assignment of the experimental bands to a specific conformer, so we assigned the 2146, 883 cm^−1^ bands in the Ar matrix and 2145, 890 cm^−1^ bands in the N_2_ matrix to the most stable fp1a with the S–H∙∙∙N intramolecular hydrogen bond and with the highest value of the calculated population equal to 83.4% (Figure S8). When the hydrogen atom migrates from the NH_2_ group to C5 after the cleavage of the S1–C2 bond in ACA, the *N*-(thiiran-2-yl)carbodiimide molecule (fp3) can be formed. The analysis of the spectrum revealed bands of fp3 photoproduct at 2141 and 881 cm^−1^ in solid Ar and 2140 and 890 cm^−1^ in solid N_2_. Additionally, absorptions due to *N*-(1-thioethan-2-yl)carbodiimide (fp2) can also be observed. On the basis of calculations, a broad absorption at 2146 cm^−1^ in Ar (2145 cm^−1^ in N_2_) and the 917 cm^−1^ band observed in an argon matrix were attributed to the fp2 photoproduct. Anharmonic calculations showed that we should expect another band of fp2 due to the νC-N + δCH_2_ combination vibrations at 2168 cm^−1^ (I_theor_ = 385 km mol^−1^), but it was not observed in the experimental spectrum. The calculations revealed stability of two fp2 conformers differing only slightly in the relative arrangement of the HN=C=N and HC=S groups and with the small relative energy difference ΔE_ZPE_ = 0.3 kJ mol^−1^ (Figure S8). Taking into account different transformations of the identified photoproducts and the fact that upon irradiation with a higher energy laser beam, the bands assigned to fp1, fp2 and fp3 decrease and the band at 2138 cm^−1^ in Ar (2139 cm^−1^ in N_2_) starts to increase, several bands that showed the same behavior as the 2138 cm^−1^ band during ACA photolysis were assigned to *N*-(1-thioethan-1-yl)carbodiimide (fp4) photoproduct. This is consistent with the fact that fp4 can be produced from the photo-tautomerization reaction of fp3 (as can be seen from Scheme 1). In addition, the values of the calculated harmonic ν_as_NCN vibration wavenumber for fp1, fp2, fp3 and fp4, i.e., 2228, 2229, 2224 and 2217 cm^−1^ (2214, 2218, 2210 and 2203 cm^−1^ in a nitrogen environment), are consistent with the position of this band in the experimental spectrum, i.e., 2146, 2146, 2141 and 2138 cm^−1^, respectively (2145, 2145, 2140 and 2139 cm^−1^ in N_2_ matrix). Anharmonic wavenumbers seem to follow this order: 2179, 2183, 2173 and 2181 cm^−1^ (2167, 2172, 2165 and 2170 cm^−1^ in N_2_ environment). In addition to the band at 2138/2139 cm^−1^, we were able to identify several other bands with the highest calculated intensities for fp4: 3499, 1415 and 1233 cm^−1^ in an argon matrix and 1416 and 1232 cm^−1^ in a nitrogen matrix, assigned to the νNH, νC–N and νC=S vibrations, respectively.

Analysis of the experimental spectrum also showed the appearance and slow increase in the intensity of the weak band at ca. 2280 cm^−1^ in both matrices during the whole photolysis process. In this range, a stretching vibration band of the C≡N group can be observed. The photolysis reaction scheme indicated several possible structures of photoproducts with a C≡N group: fp32–fp35. Comparison of the spectra calculated for these structures with the experimental spectrum showed that *N*-(1-thioethan-2-yl)cyanamide (fp33) is a possible product to consider and its presence is a direct consequence of the photo-tautomerization reaction of the photoproduct fp2, which was discussed earlier. The calculations showed stability of two conformers of fp33 differing in the relative arrangement of the HN–C≡N and HC=S groups and with the relative energy difference ΔE_ZPE_ equal to 13.5 kJ mol^−1^ (Figure S8). In addition to the band at 2278 cm^−1^ (2280 cm^−1^ in nitrogen matrix), we were able to assign two more bands to fp33: at 3387 cm^−1^ in the Ar matrix (not observed in N_2_ matrix) and 1430 cm^−1^ in the Ar matrix (1426 cm^−1^ in N_2_ matrix), which showed that a more stable conformer of this product is formed in the matrix with the highest value of the calculated population equal to 99.1% (Figure S8) (in both harmonic and anharmonic spectrum of the less stable conformer, there is only one band with an intensity more than 100 km mol^−1^).

#### 2.2.2. Formation of Cyanamide or Carbodiimide Complexes

Cleavage of the S1–C2 bond followed by disruption of the N3–C4 bond (Scheme 1) produces NH_2_**˙**C=N and **˙**CH=CH–S**˙** radicals, which change into cyanamide (fp27) and thiirene (fp28) molecules. Further photoreactions can produce carbodiimide (fp29) or ethynethiol (fp30). The spectral analysis revealed several new vibrational features present in the typical regions of cyanamide (H_2_N–C≡N) absorptions. We observed a relatively broad absorption near the cyanamide molecule νC≡N band at 2264 cm^−1^ [28,29] and several new bands in the higher wavenumber range. These bands grew during photolysis, although the rate of increase in the intensity of these bands decreased upon irradiation at wavelengths below 260 nm. The calculation results allowed us to divide the observed bands into two groups, labeled as fp27–28 and fp27–30 (Figure 2, Figure 3 and Figure 4). The absorptions assigned to group fp27–28 appeared at 3465, ca. 3200 and 2260 cm^−1^ in an argon matrix. The bands due to group fp27–30 were observed at 3470, 3375, 3310 and 2260 cm^−1^. For the discussed reaction path, we also considered the formation of carbodiimide complexes since literature reports show that carbodiimide (HN=C=NH) formation takes place by both photochemically and thermally induced isomerization from cyanamide at low temperature [28,29]. Indeed, we observed the appearance of new bands in the range of vibrations of the carbodiimide molecule [28] when irradiated with a beam of wavelength λ < 260 nm. On the other hand, ethynethiol (HC≡CSH) has previously been identified as a photoproduct of 1,2,3-thiadiazole [30] or thiazole [14] photolysis in solid argon. And we observed the appearance of a new photoproduct band at approximately 3310 cm^−1^ in the range of the strongest band of the ethynethiol molecule. Based on the calculation results, we distinguished two groups of bands fp28–29 and fp29–30 (Figure 2, Figure 3 and Figure 4) to which we assigned bands: ca. 3260, 2090, 955, 917 cm^−1^ and 3395, 3313, 2094, 903, 896 cm^−1^, respectively. As will be discussed below, the bands due to group fp27–28 and fp27–30 can be attributed to the H_2_N–C≡N + H–CSC–H and H_2_N–C≡N + HC≡CSH complexes and the bands of group fp28–29 and fp29–30 can be assigned to the HN=C=NH + H–CSC–H and HN=C=NH + HC≡CSH complexes, respectively.

**Figure 5:**
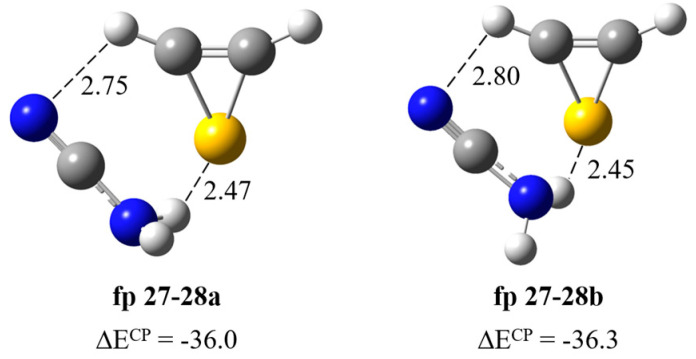
B3LYP-D3/6-311++G(3df,3pd) optimized structures of the H_2_N–C≡N∙∙∙H-CSC–H complex. The selected bond distances (in Å) and interaction energies ΔE^CP^ (in kJ mol^−1^) are given.

*Formation of H_2_N*–*C≡N*∙∙∙*H*–*CSC*–*H complex.* Figure 5 presents two structures corresponding to the stationary points calculated for the cyanamide–thiirene system. In both structures, the NH∙∙∙S bond is formed and a weak interaction of the H atom of H–CSC–H with the nitrogen atom of CN group of H_2_N–C≡N is also present. The counterpoise corrected binding energy values of the complexes ΔE^CP^ are very similar and equal to −36.0 and −36.3 kJ mol^−1^ for fp27–28a and fp27–28b but the structures differ in the geometry of the NH_2_ group: the NH_bonded_∙∙∙SC dihedral angle is 66.5 and 55.2 degrees, respectively. In Table 2, theoretical wavenumber shifts, Δν_theoretical_ = (ν_complex_ − ν_monomer_)_theoretical_, for both optimized structures are compared with the experimental ones, Δν_experimental_ = (ν_complex_ − ν_monomer_)_experimental_. The experimental wavenumber for the H_2_N–C≡N molecule was taken from Refs. [28,29]. The full sets of vibrational wavenumbers of the optimized structures are presented in Table S2. The comparison of the experimental spectra (bands assigned to the group fp27–28) with the calculated for the two structures suggest that it is not possible to clearly determine which structure of the complex is formed in the matrix. Three bands due to the H_2_N–C≡N∙∙∙H–CSC–H complex were observed in the argon matrix spectral regions of cyanamide: at 3465 cm^−1^ (Δν_exp_ = –21 cm^−1^) in the ν_as_NH_2_ region; at ca. 3200 cm^−1^ (Δν_exp_ = –200 cm^−1^) in the vicinity of ν_s_NH_2_ and at 2260 cm^−1^ (Δν_exp_ = –4 cm^−1^) in the region of the νC≡N vibration, respectively. The observed wavenumber shifts agree with the values calculated for both fp27–28 structures and we cannot rule out that both forms are present in the matrices. Unfortunately, no bands due to the perturbed thiirene molecule were identified, which may be caused by the overlapping of its bands with other absorptions as the calculations predict only low-intensity bands for this compound (see Table S2).

**Figure 6:**
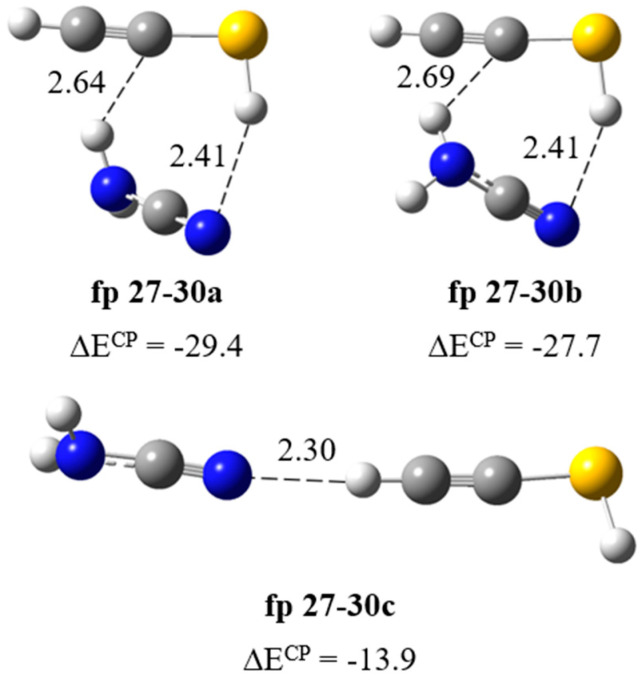
B3LYP-D3/6-311++G(3df,3pd) optimized structures of the H_2_N–C≡N∙∙∙HC≡CSH complex. The selected bond distances (in Å) and interaction energies ΔE^CP^ (in kJ mol^−1^) are given.

*Formation of H_2_N*–*C≡N∙∙∙HC≡CSH complex*. The calculations resulted in three local minima on the potential energy surface of the cyanamide–ethynethiol system that correspond to the stable structures presented in Figure 6. In the most stable structure, fp27–30a (ΔE^CP^ = −29.4 kJ mol^−1^), the cyanamide molecule serves as a proton donor towards the C≡C bond and as a proton acceptor from the SH group of ethynethiol. In the fp27–30b configuration (ΔE^CP^ = −27.7 kJ mol^−1^), similar NH∙∙∙C≡C and SH∙∙∙N interactions are present but the mutual orientation of the molecules changes to the opposite and the lengths of both hydrogen bonds differ by about 0.05 Å. In the least stable structure fp27–30c (ΔE^CP^ = −13.9 kJ mol^−1^), H_2_N–C≡N plays the role of proton acceptor of the CH group of HC≡CSH. The full sets of vibrational wavenumbers of the optimized structures are presented in Table S2. In Table 3, the theoretical wavenumber shifts for both optimized structures are compared with the experimental ones. The cyanamide and ethynethiol monomer wavenumbers were taken from various studies [14,28,29]. The comparison of the experimental spectra (bands assigned to the group fp27–30) with the calculated values for the three structures shows that it is rather difficult to determine with certainty which of the two complex structures, fp27–30a or fp27–30b, is trapped. The formation of fp27–30 in the matrix is reflected by the presence of the bands due to perturbed ν_as_NH_2_, ν_s_NH_2_, νCH, νC≡N and ωNH_2_ modes of cyanamide. The 3470, 3375 and 2260 cm^−1^ bands assigned to the perturbed NH_2_ and C≡N stretching vibrations are 16, 25 and ca. 4 cm^−1^ red shifted and the 762 cm^−1^ band attributed to the NH_2_ wagging mode is 34 cm^−1^ blue shifted with the corresponding modes of the H_2_N-C≡N monomer in solid argon, respectively. Table 3 shows that the observed pattern of the band shifts is better reflected by the shifts of the most stable complex structure fp27–30a, especially since the observed shift of the ωNH_2_ band (Δν_exp_ = +34 cm^−1^) may indicate a better agreement with the one calculated for this structure (Δν_theor_ = +42 cm^−1^).

**Figure 7:**
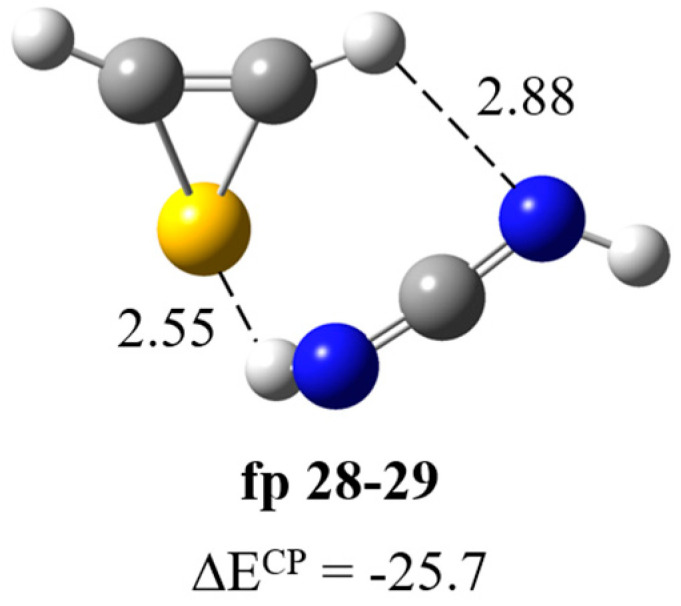
B3LYP-D3/6-311++G(3df,3pd) optimized structures of the HN=C=NH∙∙∙H–CSC–H complex. The selected bond distances (in Å) and interaction energies ΔE^CP^ (in kJ mol^−1^) are given.

*Formation of HN=C=NH*∙∙∙*H*–*CSC*–*H complex.* The results of optimization of possible structures of the carbodiimide–thiirene complex fp28–29 (Figure 7) showed that this system has a structure with a N–H∙∙∙S type interaction and a relatively parallel arrangement of molecules with respect to each other (ΔE^CP^ = −25.7 kJ mol^−1^). The full set of vibrational wavenumbers of the optimized structure is presented in Table S2. Table 4 presents theoretical and experimental wavenumber shifts for the optimized structure. The experimental wavenumbers for the HN=C=NH molecule were taken from Refs. [28,29]. The presence of the N–H∙∙∙S interaction in the complex is manifested as a significant red shift of the NH stretching vibration of the bound NH group and a relatively strong perturbation of the bending vibrations of both NH groups. We observed bands at ca. 3260, 2090, 955 and 917 cm^−1^ corresponding to the νNH_bonded_, ν_as_NCN, δNH_bonded_ and δNH_free_ vibrations, respectively.

**Figure 8:**
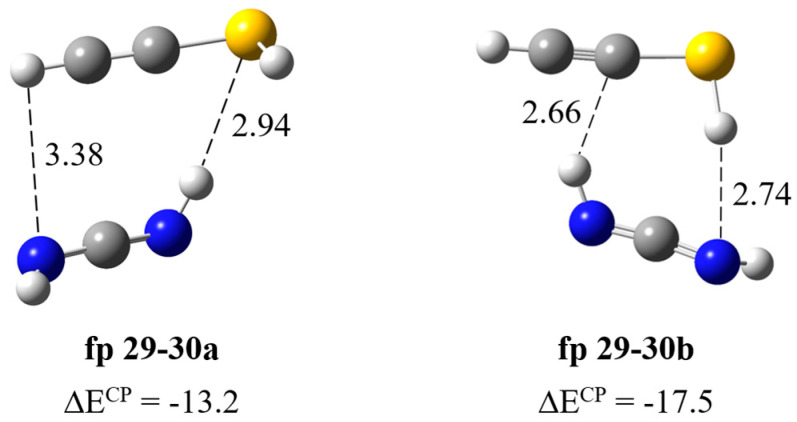
B3LYP-D3/6-311++G(3df,3pd) optimized structures of the HN=C=NH∙∙∙HC≡CSH complex. The selected bond distances (in Å) and interaction energies ΔE^CP^ (in kJ mol^−1^) are given.

*Formation of HN=C=NH∙∙∙HC≡CSH complex.* The calculations resulted in two local minima on the potential energy surface of the carbodiimide complex with ethynethiol presented in Figure 8. In the fp29–30a structure (ΔE^CP^ = −13.2 kJ mol^−1^), the NH∙∙∙S bond is formed and both interacting molecules are almost parallel to each other; the dihedral angle C=N∙∙∙C≡C is −20.8 degrees. In the structure fp29–30b (ΔE^CP^ = −17.5 kJ mol^−1^), two SH∙∙∙N and NH∙∙∙C bonds are formed, where the ethynethiol molecule acts as a both proton donor and acceptor, which explains the slightly greater stability of this complex. In Table 5, the theoretical wavenumber shifts for the fp29–30 structures are compared with the experimental ones. The full set of vibrational wavenumbers of the optimized structure is presented in Table S2. Several bands of the fp29–30 complex were observed experimentally. The 3395, 3312 and 2094 cm^−1^ bands assigned to the perturbed NH_,_ CH and N=C=N stretching vibrations are 30, 1 and ca. 4 cm^−1^ red shifted and the 903, 896 cm^−1^ bands due to the δNH_bonded_, δNH_free_ modes are 17, 10 cm^−1^ blue shifted with the corresponding modes of the HN=C=NH monomer in the argon matrix, respectively. Three bands of the complex were also observed in a nitrogen matrix: 2097, 900 and 895 cm^−1^. As one can see in Table 5, the experimental shifts are in agreement with the theoretical ones for the fp29–30a complex. Unfortunately, we did not observe the bands assigned to the SH group vibrations in the complex, which may be due to the very low intensity of these absorptions; the calculated intensities, both harmonic and anharmonic, of the νSH or δSH vibrations did not exceed 20 km mol^−1^ (see Table S2).

### 2.3. Ring Opening Reactions by Cleavage of the S1-C5 Bond

#### 2.3.1. Identification of Thiourea Derivatives

After cleavage of the S1–C5 bond, the hydrogen atom can migrate from C4 to S1, giving *N*’-ethynylcarbamimidothioic acid (fp14) as a photoproduct (see Scheme 1). As one can see in Figure 3, Figure 4, Figures S1, S6 and S7, two absorptions were observed for fp14 at 2131 and 1648 cm^−1^ in the Ar matrix and 2133 and 1650 cm^−1^ in the N_2_ matrix. These bands appeared after 5 min of irradiation at 285 nm and their intensity grew during 30 min of photolysis up to 280 nm. The performed optimization resulted in four stable structures of fp14 with different conformations of both SH and NH_2_ groups around the C=N double bond and the relative energy differences (ΔE_ZPE_) of up to 12.7 kJ mol^−1^ with respect to the structure with the highest stability (Figure S8). The comparison of the experimental bands with the absorptions calculated for the conformers of fp14 suggests that the most stable conformer fp14a with the highest population equal to 85.9% is present in the matrix with the SH group directed to the C≡C triple bond (Table 6). The photoproduct fp14a can be transformed into *N*-ethynylcarbamimidothioic acid (fp17a) with a structure similar to that of fp14a, that is, with the S–H∙∙∙C interaction and an *anti* position of the NH group. The calculations revealed eight isomeric structures for this photoproduct resulting from rotation around C–N, C–S bonds and with the relative energy difference ΔE_ZPE_ from 4.0 to 15.3 kJ mol^−1^ with respect to the structure with the highest stability and the highest population (Figure S8).

On the basis of calculation results, we were able to assign four absorptions to fp17a, namely 2162, 1641, 1283 and 1151 cm^−1^ bands in solid argon (2163, 1638 and 1284 cm^−1^ in solid nitrogen). These bands were observed after 5 min of irradiation at 285 nm and their intensity increased slowly during 65 min of photolysis up to 270 nm. As one can see in Scheme 1, fp17 may undergo a tautomerization reaction to *N*-ethenylidenecarbamimidothioic acid (fp18). Four isomeric structures were optimized for fp18 with different orientations of both SH and NH groups in relation to the C=N double bond. The most stable and populated structure fp18a differed from other isomers in energy (ΔE_ZPE_) up to 3.6 kJ mol^−1^ (Figure S8) and its most intense bands were identified at 2070, 1632 and 1220 cm^−1^ in an argon matrix. Two bands at 2060 and 1633 cm^−1^ could be assigned to fp18 in a nitrogen matrix. In turn, also on the basis of calculations, the absorptions at 2061, 1598 and 1280 cm^−1^ in the Ar matrix and at 2052 and 1283 cm^−1^ in the N_2_ matrix were attributed to fp15 photoproduct that can be formed by transferring hydrogen from the SH to the NH group of fp18 photoproduct. Anharmonic calculations showed that the appearance of a band due to the combination vibrations δNCN+δCH_2_ at 2084 cm^−1^ (I_theor_ = 531 km mol^−1^) in the spectrum of fp15 should be expected, and indeed the band at 2083 cm^−1^ was identified, which behaved identically during photolysis as the other bands assigned to fp15.

#### 2.3.2. Formation of Acetylene Complex

**Figure 9:**
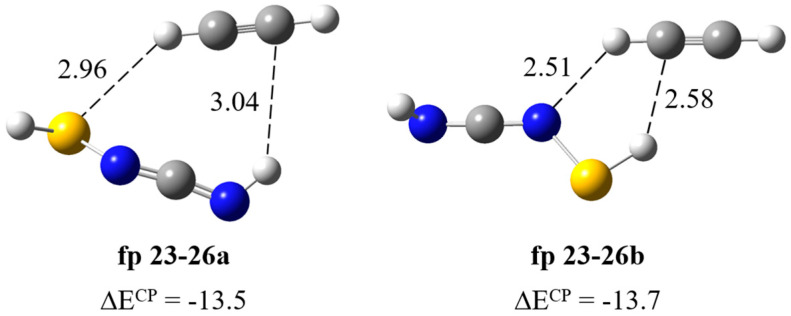
B3LYP-D3/6-311++G(3df,3pd) optimized structures of the HS–N=C=NH∙∙∙HC≡CH complex. The selected bond distances (in Å) and interaction energies ΔE^CP^ (in kJ mol^−1^) are given.

*Formation of HS*–*N=C=NH∙∙∙HC≡CH complex.* As can be seen in Scheme 1, after the cleavage of the S1–C5 bond, the N3–C4 bond can be disrupted to co–produce CH≡CH (fp23) and thiaziren-3-amine (fp24) molecules. The calculations showed stability of two fp23–24 complex structures in which the acetylene molecule plays the role of proton donor or proton acceptor and predicted that the strongest band should appear at 1832 cm^−1^ (I_theor_ = ca. 260 km mol^−1^). A careful spectral analysis indicates that there is no photoproduct band in this wavenumber range, so a complex does not form in the matrix or immediately undergo further photoreactions. The tautomerization products of thiaziren-3-amine (fp24) are thiaziridin-3-imine (fp25) and *N*-thiolcarbodiimide (fp26) (Figure S4); therefore, we optimized possible structures of the fp23–25 and fp23–26 complexes. Similarly to fp23–24, calculations predict the formation of two structures of the fp23–25 complex and the strongest band at ca. 1840 cm^−1^ (I_theor_ = ca. 340 km mol^−1^), which is not present in the experimental spectrum. In turn, for the fp23–26 complex, two structures were optimized with CH∙∙∙S and SH∙∙∙C or SH∙∙∙C and CH∙∙∙N interactions and very similar binding energy values ΔE^CP^ equal to −13.5 and −13.7 kJ mol^−1^ for fp23–26a and fp23–26b, respectively (Figure 9). In Table 7, the theoretical wavenumber shifts for the fp23–26 structures are compared with the experimental ones and the full set of vibrational wavenumbers of the optimized structures is presented in Table S2. In the region of the carbodiimide group vibration, ν_as_N=C=N, a distinct band appears after matrix irradiation (at 2100 cm^−1^ in both matrices), which suggests formation of the fp23–26 complex. This band is located in close proximity to the 2094 cm^−1^ absorption due to the ν_as_NCN mode of the already assigned carbodiimide complex HN=C=NH∙∙∙HC≡CSH (fp29–30). The presence of fp23–26 complex is also supported by the appearance of the 924 cm^−1^ band due to the δNH vibration of HS–N=C=NH and by the ν_s_CH and δ_s_CH vibrations of HC≡CH at 3178 and 747 cm^−1^, respectively. Two bands were also identified at 929 and 755 cm^−1^ in a nitrogen matrix. The calculated wavenumber shift did not allow for unambiguous assignment of the experimental bands to a specific fp23–26 complex geometry. Some inconsistency in these values may be due to the lack of experimental wavenumbers for *N*-thiolcarbodiimide. The anharmonic wavenumber of ν_as_NCN in the monomer (2161 cm^−1^) is much higher than the experimental wavenumber of the perturbed ν_as_NCN vibration in the complex (2100 cm^−1^). Most likely, the anharmonicity of this vibration is greater than the calculations predict, which may be due to the coupling of the stretching vibrations of the NCN group and the NH group present in the close vicinity. A similar situation occurs for photoproduct molecules, e.g., fp1, fp2, fp3 or fp14, for which the ν_as_NCN vibration bands were observed at ca. 2140 cm^−1^, and the calculations predicted the anharmonic wavenumbers between 2183 and 2171 cm^−1^ for this mode.

#### 2.3.3. Complexes of Isothiocyanic Acid and Its Isomers

Similarly to the photolysis of thiazole [14], in the spectral range of 2050–2000 cm^−1^ and after irradiation of ACA, new bands appeared, which may indicate the formation of molecules with accumulated multiple bonds like N=C=S or C=C=N. As can be seen from Scheme 1, photolysis of 2-aminothiazole via tautomerization to 2-iminothiazole could give HNCS (fp36) molecules and its isomers as well as acetonitrile (CH_3_CN, fp40) or ketenimine (CH_2_CNH, fp39); on the other hand, photodissociation of fp15 photoproduct would also directly lead to the HNCS-CH_2_CNH complex. The most visible photoproduct band in the discussed region is an absorption band at ca. 2017 cm^−1^ (2020 cm^−1^ in a nitrogen matrix), which appeared after 5 min of irradiation at 285 nm and grew for 50 min up to λ = 280 nm. After further irradiation at λ < 280 nm, the intensity of these bands decreased strongly, while the intensity of the remaining bands in this range slowly increased up to 270 nm. Tentatively, we assigned 2017 cm^−1^ bands to the HNCS-CH_2_CNH complex (fp36–39) in which isothiocyanic acid acts as a proton donor towards ketenimine. Calculations show that the νCN vibration of HNCS after complex formation should be shifted by 34 cm^−1^ towards higher frequencies with respect to the monomer band (1981.8 cm^−1^ in an argon matrix [32,33]), so the νCN band of the complex should appear at 2016 cm^−1^, which agrees with the observed wavenumber. The ν_as_CCN vibration of ketenimine is practically not shifted in the complex so it should be located at ca. 2040 cm^−1^ in solid Ar [34] and indeed there is a band at ca. 2040 cm^−1^ in the argon matrix spectra (2039 cm^−1^ in a nitrogen matrix), as shown by bands A in Figure 3. Unfortunately, we were not able to observe the NH stretching vibration of the proton donor in the complex, which should be a very intense absorption located at the 3200–3100 cm^−1^ region. In this area, as a result of irradiation, numerous broad bands appeared, but their intensities increased throughout the photolysis. It is possible that the broad and diffuse band of the HNCS-CH_2_CNH complex was covered by other photoproduct bands of in this range. For comparison, the νNH vibration was identified at 3492.6, 3459.4 and 3435.5 cm^−1^, while the νCN band was observed at 1986.7, 1997.3 and 2005.5 cm^−1^ for HNCS-N_2_, HNCS-CO and HNCS-SO_2_ complexes, respectively [33,35,36].

Due to the fact that a weak νCN vibration of thiofulminic acid (HCNS) at 2034 cm^−1^ was observed during photolysis of the thiazole molecule [14], we decided to perform calculations of possible complexes of HCNS with ketenimine (similar to the HNCS molecule) and obtained three stable structures of the HCNS-CH_2_CNH complex where the NH group of ketenimine acts as a proton donor or acceptor towards the HCNS molecule. The calculated wavenumber shifts indicate that these complexes could probably form in the matrix due to the number of experimental bands in the vicinity of the monomer vibrations: CH_2_CNH at 2040 cm^−1^ [34] and HCNS at 2035 cm^−1^ [37]. So, following these observations, we assigned 2033 and 2027 cm^−1^ bands to complex D; 2047 and 2043 cm^−1^ to complex E; 2051 and 2041 cm^−1^ to complex F (Figure 3 and Table S5). However, this is only an approximate assignment, and additional experimental work is necessary to confirm the above considerations.

## 3. Materials and Methods

The solid sample of ACA (2-aminothiazole-4-carboxylic acid, 98%, abcr GmbH) was allowed to sublimate at 424 K in a small electrical oven assembled inside the closed-cycle helium cryostat (ARS-2HW, APD-Cryogenics). Deposition of an ACA vapor mixed with a large excess of a matrix gas (Ar or N_2_) onto a cold CsI window was performed to prepare ACA/Ar and ACA/N_2_ matrices. The temperature of the matrix window was maintained at 15 K. FTIR spectra were recorded at 10 K between 4000 and 400 cm^−1^ by means of a Nicolet iS50 FTIR spectrometer with a resolution of 0.5 cm^−1^ and using a liquid N_2_ cooled MCT detector. Photochemical reactions were induced in ACA/Ar(N_2_) matrices by UV radiation of a pulsed optical parametric oscillator named Vibrant (Opotek, Inc., Carlsbad, CA, USA) pumped with a pulsed Nd:YAG laser (Quantel). The experiments started using λ = 300 nm light and proceeded with gradual decreases in the output wavelength. The process was controlled by recording infrared spectra of the matrix after each irradiation.

All calculations were performed using the Gaussian 16 [38] program. Structures of photoproducts were optimized at the B3LYP-D3/6-311++G(3df,3pd) level [39,40] of theory with D3 dispersion correction [41,42]. Force constant matrices were calculated at the same level of theory to evaluate harmonic frequencies and zero-point vibrational corrections. Anharmonic wavenumbers were calculated for the selected monomeric species at the B3LYP-D3/6-311++G(3df,3pd) level of theory by the vibrational perturbation theory (VPT2) [43,44,45]. In the case of calculations performed in a nitrogen environment, the SMD-IEFPCM solvation model (ε = 1.546) was included [46]. The structures of the complexes were also optimized at the same level of theory and their binding energies were corrected by the Boys–Bernardi full counterpoise procedure (CP) [47].

## 4. Conclusions

The UV laser-induced photodegradation of 2-aminothiazole-4-carboxylic acid was studied by the infrared matrix isolation method for the first time. As the first step, decarboxylation occurred, which produced CO_2_ and 2-aminothiazole molecules. Next, two main mechanisms that initiated subsequent photochemical reactions were proposed taking into account the research on thiazole photolysis [14]. The ring-opening photoreactions caused by the cleavage of the S1–C2 bond lead to the formation of **˙**C(NH_2_)=N-CH=CH-S**˙** biradicals. The hydrogen atom of the NH_2_ group of the radical migrated to S1 to form *N*-(1-sulfanylethen-2-yl)carbodiimide (fp1) or to C5 to produce *N*-(thiiran-2-yl)carbodiimide molecule (fp3); further photochemical reactions may transform both fp1 and fp3 photoproducts into *N*-(1-thioethan-2-yl)carbodiimide (fp2) as shown in Scheme 1. Additionally, fp2 may photo-tautomerize into *N*-(1-thioethan-2-yl)cyanamide (fp33) and fp3 may turn into *N*-(1-thioethan-1-yl)carbodiimide (fp4) photoproduct. Cleavage of the S1–C2 bond followed by disruption of the N3–C4 bond produces the cyanamide molecule (fp27) and the **˙**CH=CH-S**˙** biradical that transforms into thiirene (fp28); subsequent photoreactions can produce carbodiimide (fp29) or ethynethiol (fp30). The ring-opening photoreaction caused by the cleavage of the S1–C5 bond occurred simultaneously. The hydrogen-atom migration from C4 to S1 in **˙**S-C(NH_2_)=N-CH=CH**˙** forms *N*’-ethynylcarbamimidothioic acid (fp14) as a photoproduct, which becomes a substrate in the following chain of transformations: fp14 → fp17 (*N*-ethynylcarbamimidothioic acid) → fp18 (*N*-ethenylidenecarbamimidothioic acid) → fp15 (*N*-ethenylidenethiourea). Cleavage of the S1–C5 bond followed by disruption of the N3–C4 bond produces the acetylene molecule (fp23) and **˙**S-C(NH_2_)=N**˙** biradical that transforms into *N*-thiolcarbodiimide (fp26). We also observed several new bands appearing after ACA irradiation in the range of 2050–2000 cm^−1^, which, based on the literature and calculations, could most likely be attributed to isothiocyanic acid and thiofulminic acid complexes with ketenimine: HNCS-CH_2_CNH and HCNS-CH_2_CNH. All larger photoproducts containing sulphur were identified for the first time; *N*-thiolcarbodiimide and thiirene, among the small molecules, as well as all molecular complexes, were newly observed.

It is worth mentioning that cyanamide [48], acetylene [49], carbodiimide [50] or ketenimine [51] molecules discussed in this paper have been identified in space, so their molecular complexes may have potential significance for astrophysics, astrochemistry or even astrobiology. Our results confirm that complex organic compounds undergo photodissociation into simpler prebiotic building blocks. More generally, the dependence of photoproducts on precursor composition suggests the need for studies involving in situ matrix isolation and UV irradiation of complex organic molecules or clusters to decipher molecular synthesis pathways under astrochemical conditions and better understand the evolution of interstellar ice [52,53].

## Figures and Tables

**Table 1:** Experimental and B3LYP-D3/6-311++G(3df,3pd) calculated wavenumbers (cm^−1^) and intensities (km mol^−1^) of fp1, fp2, fp3, fp4 and fp33 photoproduct molecules observed after photolysis of ACA/Ar and ACA/N_2_ matrices.

ACA/Ar			ACA/N_2_				
fp1							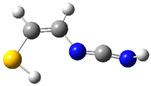
ν exp	ν harm	ν anharm	ν exp	ν harm	ν anharm	Assignmt ^b^	
2146	2228 (1441) ^a^	2179	2145	2214 (1430)	2167	ν_as_N=C=N	
883	906 (431)	838	890	906 (403)	849	δNH	
fp2							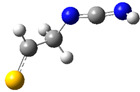
ν exp	ν harm	ν anharm	ν exp	ν harm	ν anharm	Assignmt	
2146	2229 (1148)	2183	2145	2218 (1095)	2172	ν_as_N=C=N	
917 br	932 (277)	869	-	936 (275)	895	δNH	
fp3							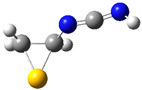
ν exp	ν harm	ν anharm	ν exp	ν harm	ν anharm	Assignmt	
2141	2224 (1259)	2173	2140	2210 (1222)	2165	ν_as_N=C=N	
881	910 (349)	846	890	911 (330)	865	δNH	
fp4							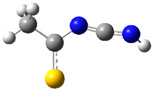
ν exp	ν harm	ν anharm	ν exp	ν harm	ν anharm	Assignmt	
3499	3628 (171)	3460	-	3624 (175)	3452	νNH	
2138	2217 (1271)	2181	2139	2203 (1243)	2170	ν_as_N=C=N	
1415	1442 (211)	1418	1416	1442 (220)	1414	νC-N, ν_s_N=C=N	
1233	1238 (200)	1213	1232	1230 (208)	1207	ωCH_3_, νC=S,	
fp33							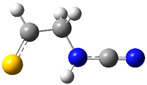
ν exp	ν harm	ν anharm	ν exp	ν harm	ν anharm	Assignmt	
3387	3506 (134)	3335	-	3501 (138)	3337	νNH	
2278	2334 (237)	2290	2280	2328 (258)	2281	νC≡N	
1430	1480 (100)	1450	1426	1476 (99)	1445	δNH, δCH_2_	

^a^ The IR calculated intensities expressed in km mol^−1^. ^b^ Abbreviations: ν—bond stretching, δ—bending, deformation in plane, ω—wagging; br—broad.

**Table 2:** Wavenumbers and wavenumbers shifts (Δν = (ν_compl_ − ν_mon_)) (in cm^−1^) calculated and observed for the H_2_N–C≡N∙∙∙H–CSC–H complex in solid argon and nitrogen ^a^.

H_2_N–C≡N∙∙∙H–CSC–H						
Experimental		Calculated harmonic				Assignment
		fp27–28a		fp27–28b		
ν	Δν	ν	Δν	ν	Δν	
3465	−21	3605	−33	3600	−38	ν_as_NH_2_
3200 br	−200	3348	−199	3336	−211	ν_s_NH_2_
2260 [2270]	−4	2336	−16	2336	−16	νC≡N

^a^ The experimental wavenumbers for the H_2_N–C≡N monomer were taken from Refs. [28,29]. Wavenumbers from nitrogen matrix are given in brackets.

**Table 3:** Wavenumbers and wavenumber shifts (Δν = (ν_compl_ − ν_mon_)) (in cm^−1^) calculated and observed for the H_2_N–C≡N∙∙∙HC≡CSH complex in solid argon and nitrogen ^a^.

H2N–C≡N∙∙∙HC≡CSH								
Experimental		Calculated harmonic						Assignment
		fp27–30a		fp27–30b		fp27–30c		
ν	Δν	ν	Δν	ν	Δν	ν	Δν	
3470	−16	3618	−19	3621	−16	3640	3	νasNH2
3375 [3370]	−25	3498	−50	3503	−45	3548	0	νsNH2
3310	−3	3461	−7	3462	−6	3387	−81	νCH
2260 [2270]	−4	2340	−12	2341	−11	2358	6	νC≡N
762	34	609	42	572	5	551	−16	ωNH2

^a^ The experimental wavenumbers for the monomers were taken from Refs. [14,28,29]. Wavenumbers from nitrogen matrix are given in brackets.

**Table 4:** Wavenumbers and wavenumber shifts (Δν = (ν_compl_ − ν_mon_)) (in cm^−1^) calculated and observed for the HN=C=NH∙∙∙H–CSC–H complex in solid argon and nitrogen ^a^.

HN=C=NH∙∙∙H–CSC–H				
Experimental		Calculated harmonic		Assignment
		fp28–29		
ν	Δν	ν	Δν	
3260 [3260]	−165	3406	−200	νNH_bonded_
2090 [2090]	−8	2212	−7	ν_as_NCN
955	69	974	60	δNH_bonded_
917 br	31	954	40	δNH_free_

^a^ The experimental wavenumbers for the HNCNH monomer were taken from Refs. [28,29]. Wavenumbers from nitrogen matrix are given in brackets.

**Table 5:** Wavenumbers and wavenumber shifts (Δν = (ν_compl_ − ν_mon_)) (in cm^−1^) calculated and observed for the HN=C=NH∙∙∙HC≡CSH complex in solid argon and nitrogen ^a^.

HN=C=NH∙∙∙HC≡CSH						
Experimental		Calculated harmonic				Assignment
		fp29–30a		fp29–30b		
ν	Δν	ν	Δν	ν	Δν	
3395	−30	3567	−33	3553	−42	νNH_bonded_
3312	−1	3465	−3	3463	−5	νCH
2094 [2097]	−4	2216	−3	2216	−3	ν_as_NCN
903 [900]	17	936	22	944	30	δNH_bonded_
896 [895]	10	925	11	936	22	δNH_free_

^a^ The experimental wavenumbers for the monomers were taken from Refs. [14,28,29]. Wavenumbers from nitrogen matrix are given in brackets.

**Table 6:** Experimental and B3LYP-D3/6-311++G(3df,3pd) calculated wavenumbers (cm^−1^) and intensities (km mol^−1^) of fp14, fp15, fp17 and fp18 photoproduct molecules observed after photolysis of ACA/Ar and ACA/N_2_ matrices.

ACA/Ar			ACA/N_2_				
fp14							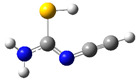
ν exp	ν harm	ν anharm	ν exp	ν harm	ν anharm	Assignmt ^b^	
2131	2191 (165) ^a^	2160	2133	2185 (186)	2152	νC≡C	
1648	1663 (612)	1624	1650	1652 (594)	1606	δNH_2_, νC=N	
fp15							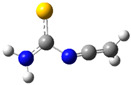
ν exp	ν harm	ν anharm	ν exp	ν harm	ν anharm	Assignmt	
2061	2125 (685)	2094	2052	2118 (680)	2069	ν_as_N=C=C	
1598	1624 (222)	1584	-	1624 (210)	1557	δNH_2_	
1280	1304 (232)	1287	1283	1301 (242)	1270	νC-N, ρNH_2_	
2083	-	2084	-	-	2069	δNCN+δCH_2_	
fp17							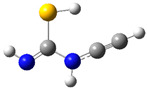
ν exp	ν harm	ν anharm	ν exp	ν harm	ν anharm	Assignmt	
2162	2233 (140)	2192	2163	2229 (138)	2189	νC≡C	
1641	1662 (418)	1631	1638	1652 (427)	1621	νC=N	
1283	1319 (140)	1271	1284	1320 (135)	1270	νC-N, δNH	
1151	1186 (137)	1154	-	1186 (136)	1151	δNH, νC-N	
fp18							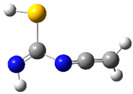
ν exp	ν harm	ν anharm	ν exp	ν harm	ν anharm	Assignmt	
2070	2126 (688)	2075	2060	2120 (681)	2072	ν_as_N=C=C	
1632	1672 (290)	1632	1633	1665 (291)	1623	νC=N	
1220	1274 (230)	1237	-	1273 (225)	1239	δNH, νC-N	

^a^ The IR calculated intensities expressed in km mol^−1^. ^b^ Abbreviations: ν—bond stretching, δ—bending, deformation in plane; ρ—rocking.

**Table 7:** Wavenumbers and wavenumber shifts (Δν = (ν_compl_ − ν_mon_)) (in cm^−1^) calculated and observed for the HS–N=C=NH∙∙∙HC≡CH complex in solid argon and nitrogen ^a^.

HS–N=C=NH∙∙∙HC≡CH						
Experimental		Calculated harmonic				Assignment
		fp23–26a		fp23–26b		
ν	Δν	ν	Δν	ν	Δν	
3178	−110	3389	−122	3389	−122	νsCH
2100 [2100]	−61 ^b^	2208	−4	2209	−3	νasNCN
924 [929]	40 ^b^	960	16	942	−2	δNH
747 [755]	11	794	32	787	25	δsCH

^a^ The experimental wavenumbers for the acetylene monomer were taken from Ref. [31]; for the HN=C=N–SH monomer, the anharmonic wavenumbers were used for Δν_exp_ calculations (Table S1). ^b^ The magnitude of this shift is explained in the text. Wavenumbers from nitrogen matrix are given in brackets.

## Data Availability

The data presented in this study are available in this article.

## Supplementary Materials

The following supporting information can be downloaded at https://www.mdpi.com/article/10.3390/molecules30183713/s1: Figure S1: The 2200–2100 cm^−1^ region of the ACA/Ar matrix spectra during UV irradiation at wavelengths between 285 and 230 nm; Figure S2: The asymmetric stretching vibrations of CO_2_ region in the ACA/Ar matrix spectra during UV irradiation at wavelengths between 270 and 230 nm; Figure S3: Possible photolysis pathways of ACA. Part 1; Figure S3: Possible photolysis pathways of ACA. Part 1; Figure S5: Possible photolysis pathways of ACA. Part 3; Figure S6: Top: The 2295–1965 cm^−1^ region of the ACA/N_2_ spectra after deposition at 15 K/10 K (black traces), after 15 min irradiation at 270 nm (pink traces), 15 min irradiation at 255 nm (green traces), 30 min irradiation at 230 nm (blue traces) and B3LYP-D3/6-311++G(3df,3pd) stick spectra of the identified photoproducts; Figure S7: The 1675–1615 and 965–875 cm^−1^ regions of the ACA/N_2_ spectra presented in Figure S6; Figure S8: Conformers of identified photoproducts with relative energy (ΔE_ZPE_) values (in kJ mol^−1^) and calculated population (%); Table S1: Anharmonic and harmonic wavenumbers (in cm^−1^) calculated for ACA photoproducts at the B3LYP-D3/6-311++G(3df,3pd) level of theory. The IR calculated intensities expressed in km mol^−1^. The combination wavenumbers with intensity > 100 km mol-1 are included. Assignment: ν—stretching modes and δ—bending modes of all kinds; Table S2: Anharmonic and harmonic wavenumbers (in cm^−1^) calculated for ACA complexes at the B3LYP-D3/6-311++G(3df,3pd) level of theory. The IR calculated intensities expressed in km mol^−1^. Assignment: ν—stretching modes and δ—bending modes of all kinds; Table S3: Optimized structures (B3LYP-D3/6-311++G(3df,3pd)) and geometrical parameters of the ACA photoproduct molecules. Bond lengths (R) are given in Ångstroms, valence (A) and dihedral (D) angles are given in degrees; Table S4: Cartesian coordinates (B3LYP-D3/6-311++G(3df,3pd)) of the identified ACA photoproduct molecules; Table S5: Calculated and argon matrix experimental wavenumber shifts (in cm^−1^) in HNCS-CH_2_CNH and HCNS-CH_2_CNH complexes with interaction energies ΔE^CP^ (in kJ mol^−1^).
