# Supplementary material for: UV-Induced Photodegradation of 2-Aminothiazole-4-Carboxylic Acid: Identification of New Carbodiimide Molecules
Source: Molecules. 2025 Sep 12;30(18):3713. doi: 10.3390/molecules30183713 (PMC12472919; doi:10.3390/molecules30183713)
Supplement: Supplementary file 1 [file molecules-30-03713-s001.zip › molecules-3805670-supplementary.pdf]

# Supplementary Materials

**UV–induced photodegradation of 2-aminothiazole-4-carboxylic acid: identification of new carbodiimide molecules**

Daria Bumażnik and Magdalena Sałdyka*

*Faculty of Chemistry, University of Wrocław, F. Joliot-Curie 14, 50-383 Wrocław, Poland; daria.bumaznik@chem.uni.wroc.pl (D.B.); magdalena.saldyka@chem.uni.wroc.pl (M.S.)*

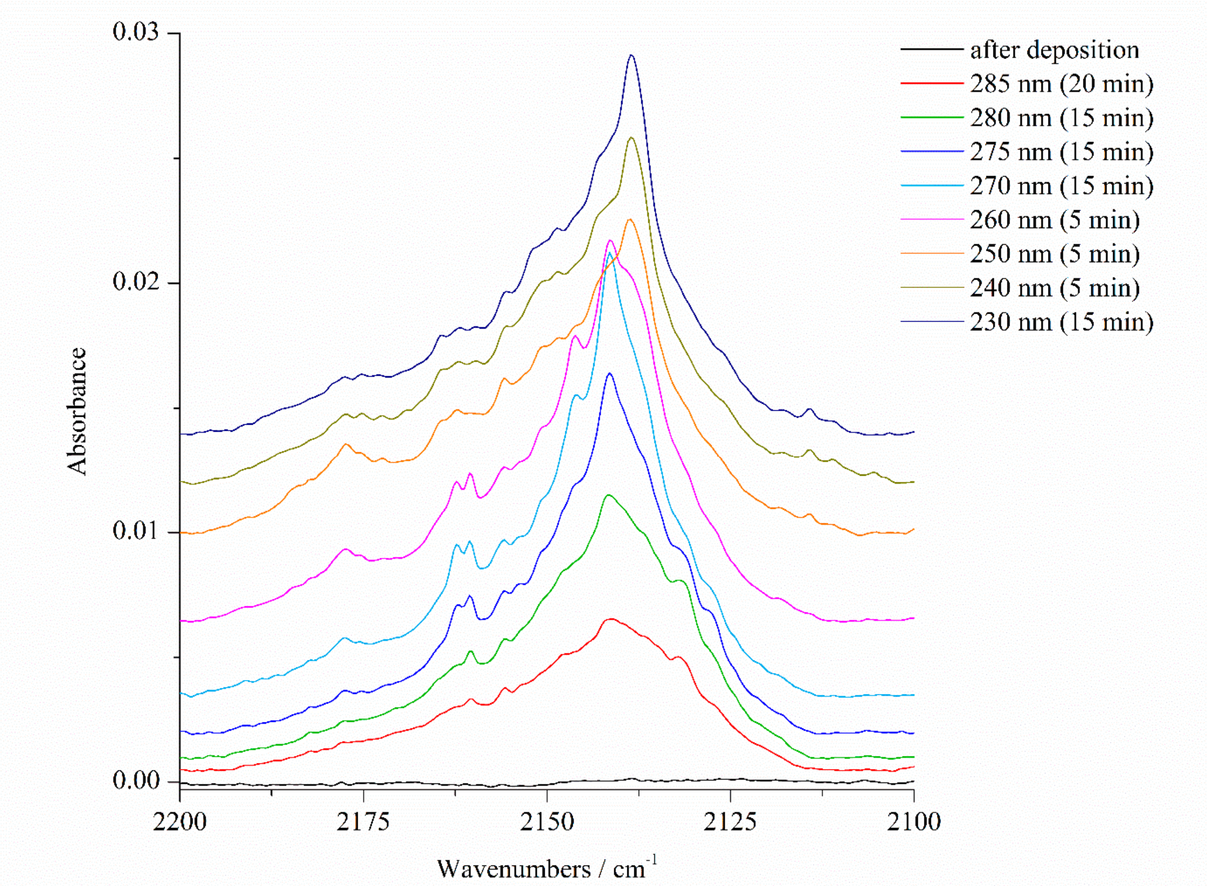


**Figure S1.** The 2200-2100 cm$^{-1}$ region of the ACA/Ar matrix spectra during UV irradiation at wavelengths between 285 and 230 nm.

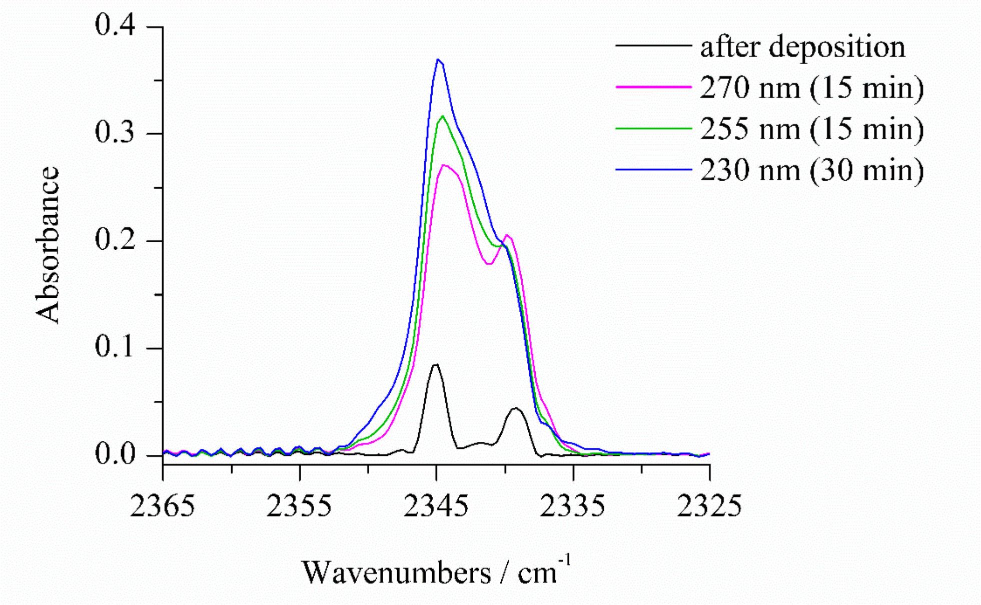


**Figure S2.** The asymmetric stretching vibrations of $CO_2$ region in the ACA/Ar matrix spectra during UV irradiation at wavelengths between 270 and 230 nm.

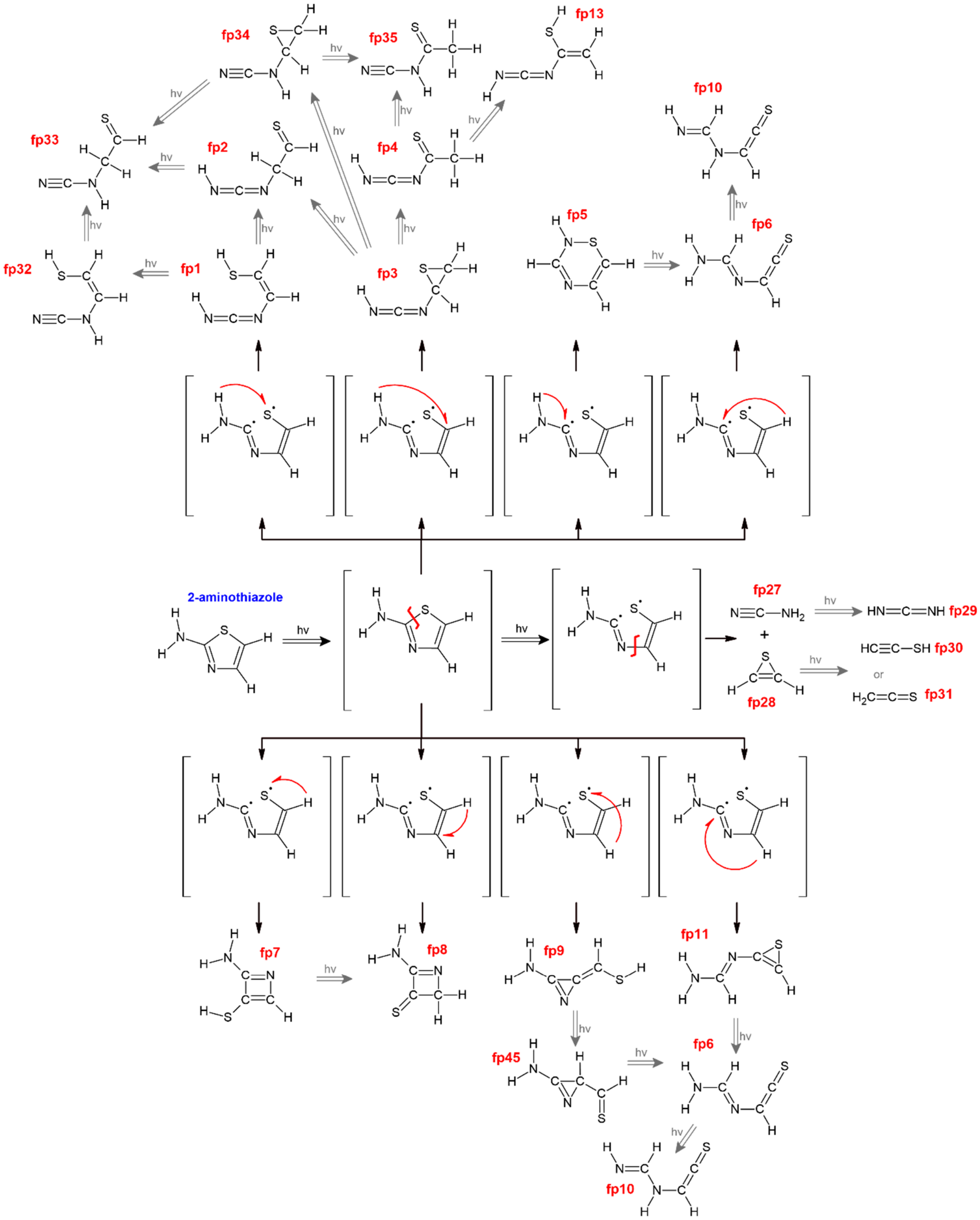


**Figure S3.** Possible photolysis pathways of ACA. Part 1.

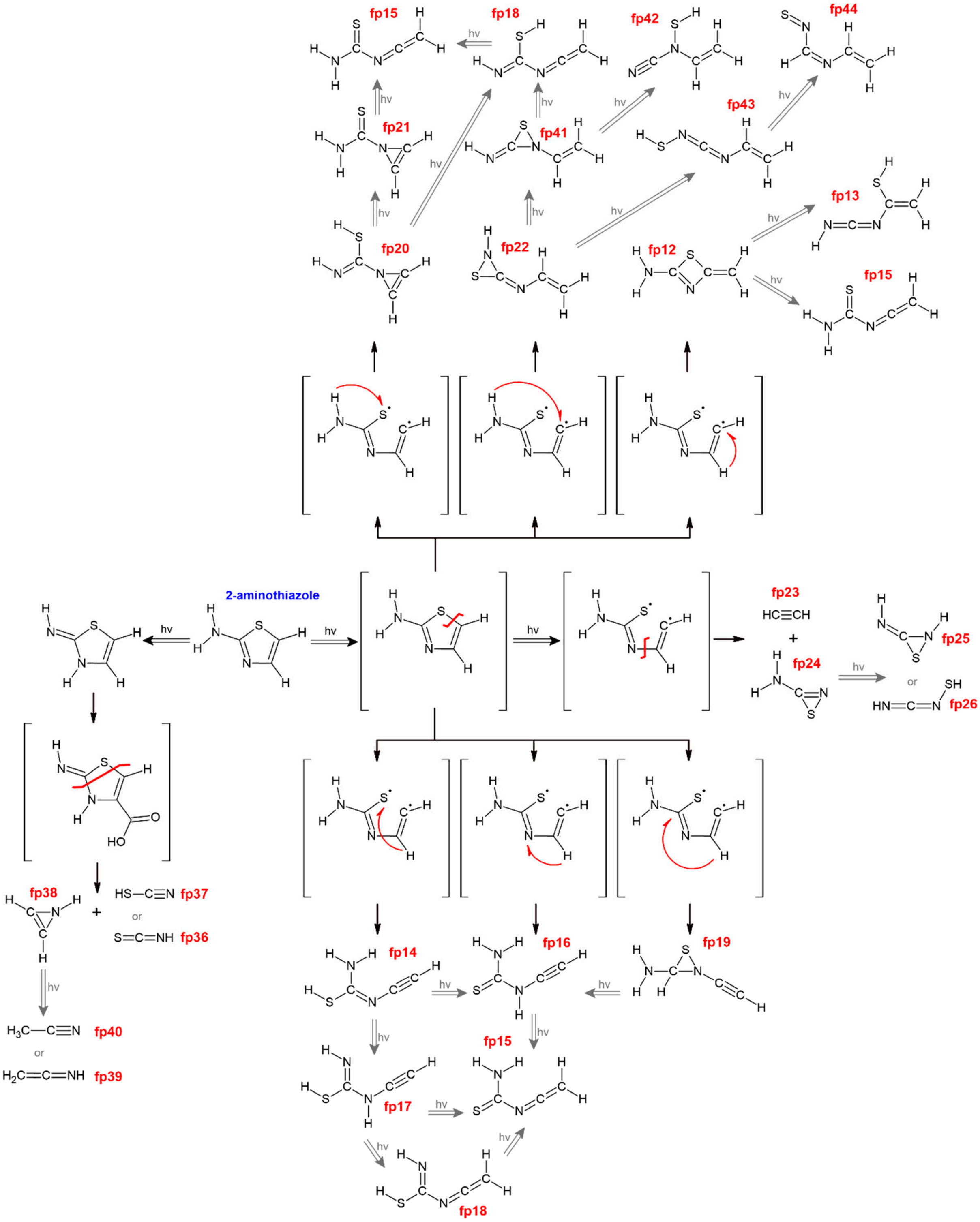


**Figure S4.** Possible photolysis pathways of ACA. Part 2.

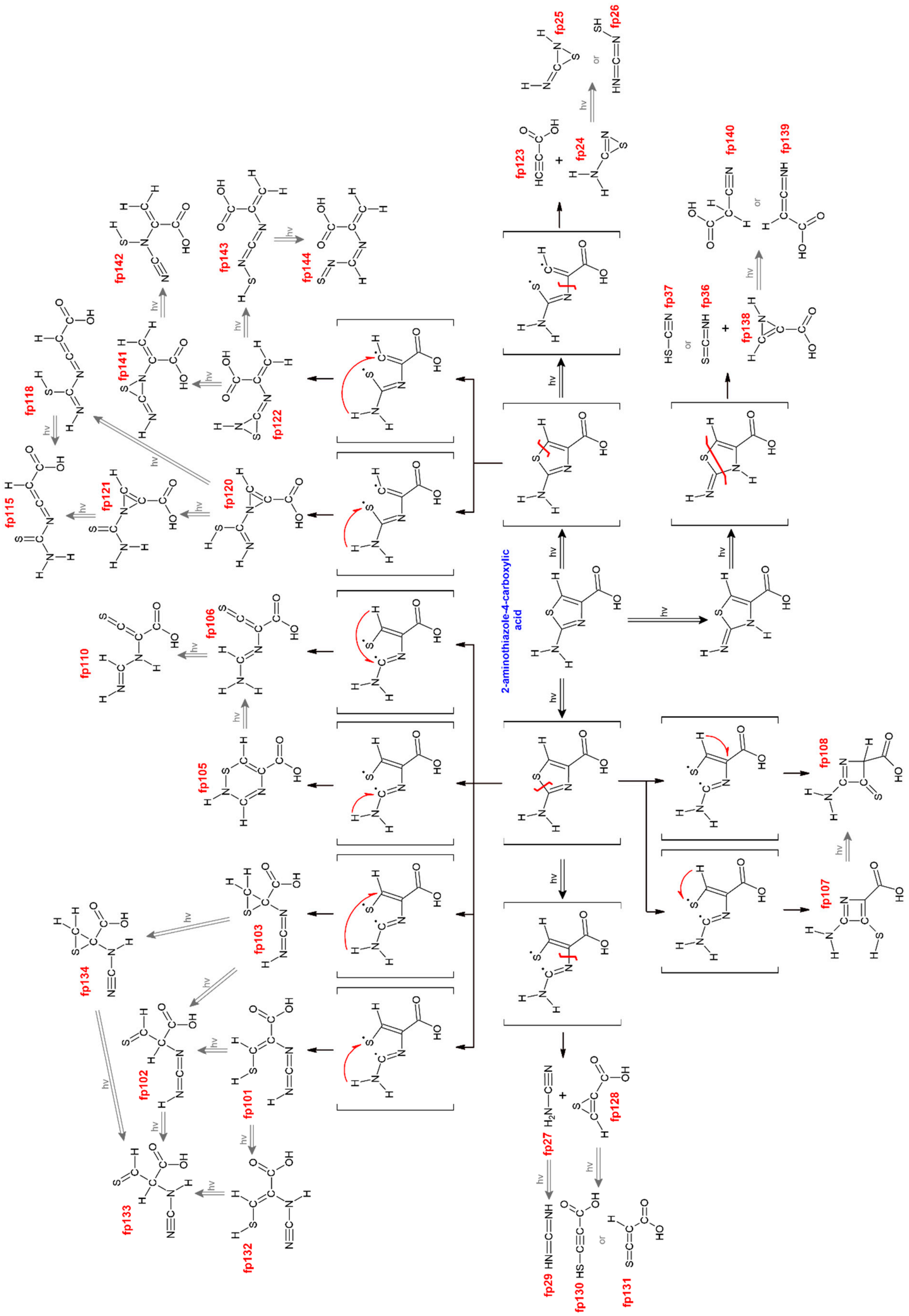


**Figure S5.** Possible photolysis pathways of ACA. Part 3.

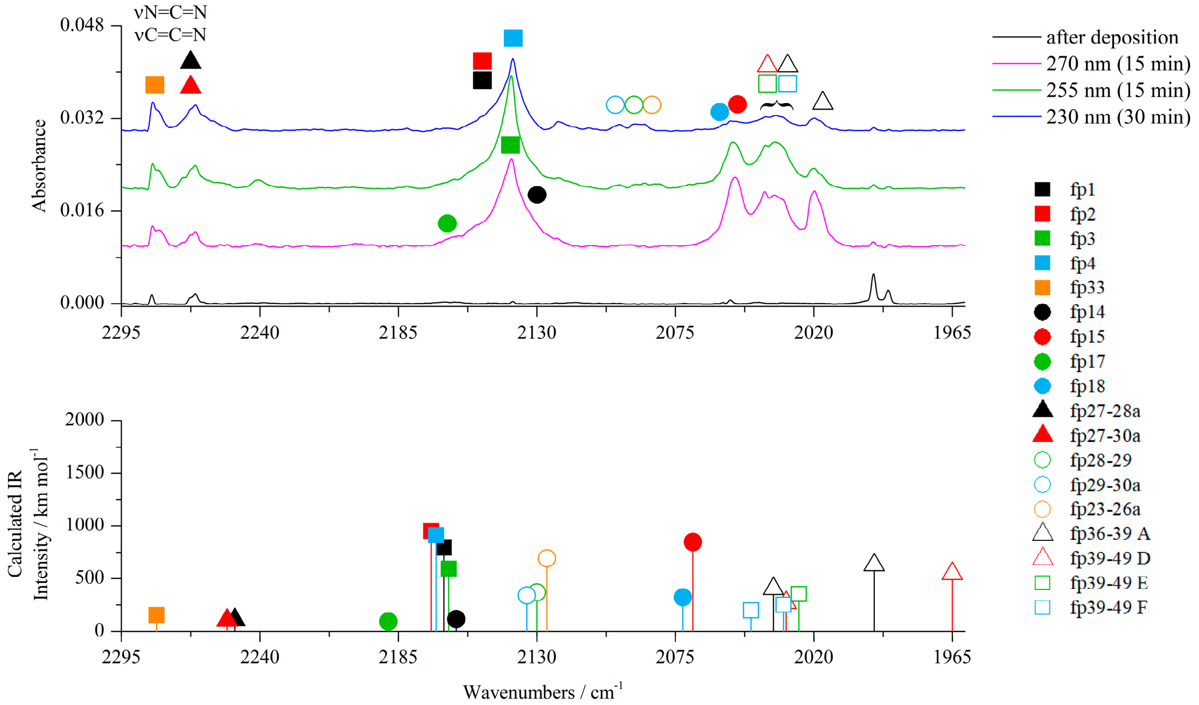


**Figure S6.** Top: The 2295-1965 cm$^{-1}$ region of the ACA/$N_2$ spectra after deposition at 15 K/10 K (black traces), after 15 min irradiation at 270 nm (pink traces), 15 min irradiation at 255 nm (green traces), 30 min irradiation at 230 nm (blue traces) and B3LYP-D3/6-311++G(3df,3pd) stick spectra of the identified photoproducts. For molecular complexes, it was not possible to include the SMD-IEFPCM solvation model in the calculations, therefore the band positions were marked on the basis of calculations performed for the gas phase.

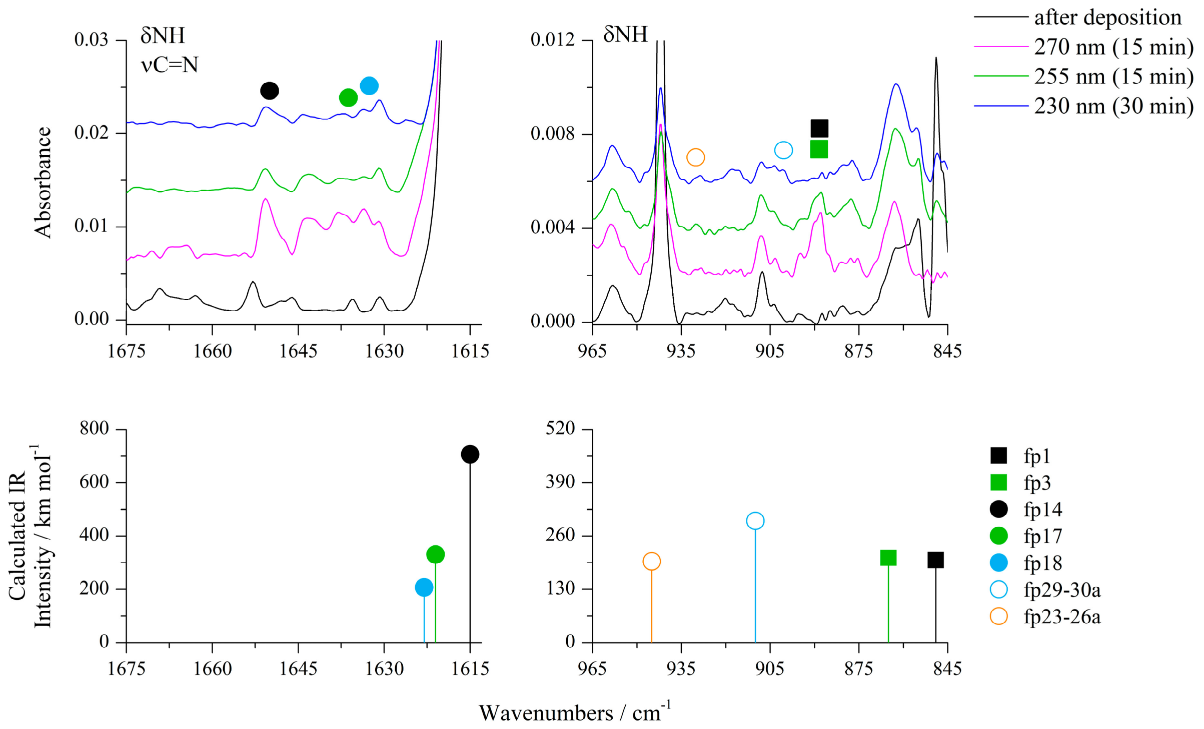


**Figure S7.** The 1675-1615 and 965-875 cm$^{-1}$ regions of the ACA/$N_2$ spectra presented in Fig. S6.

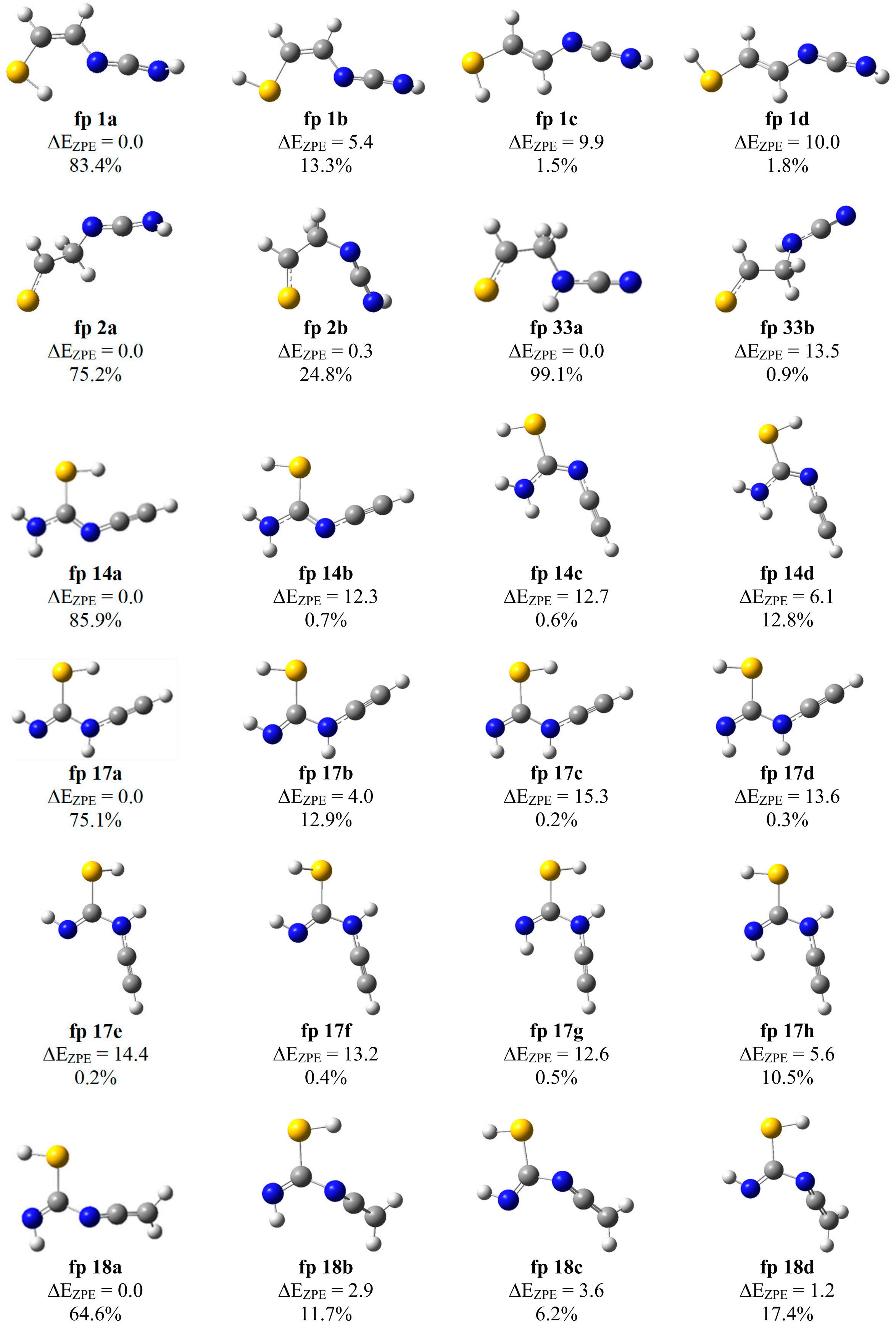


**Figure S8.** Conformers of identified photoproducts with relative energy ($\Delta E_{ZPE}$) values (in kJ $mol^{-1}$) and calculated population (%).

**Table S1.** Anharmonic and harmonic wavenumbers (in $cm^{-1}$) calculated for ACA photoproducts at the B3LYP-D3/6-311++G(3df,3pd) level of theory. The IR calculated intensities expressed in km $mol^{-1}$. The combination wavenumbers with intensity > 100 km $mol^{-1}$ are included. Assignment: ν – stretching modes, δ – bending modes of all kinds.

| **2-aminothiazole** | | | | | **2-aminothiazole / $N_2$** | | | | |
|---|---|---|---|---|---|---|---|---|---|
| Mode | $E_{harm}$ | $E_{anharm}$ | $I_{harm}$ | $I_{anharm}$ | Mode | $E_{harm}$ | $E_{anharm}$ | $I_{harm}$ | $I_{anharm}$ |
| ν1 | 3671.58 | 3514.35 | 35.88 | 28.65 | ν1 | 3671.38 | 3510.93 | 36.28 | 31.22 |
| ν2 | 3562.98 | 3417.43 | 42.80 | 37.35 | ν2 | 3563.25 | 3416.75 | 44.55 | 40.63 |
| ν3 | 3260.07 | 3130.00 | 1.10 | 1.73 | ν3 | 3260.36 | 3130.20 | 1.18 | 1.59 |
| ν4 | 3211.41 | 3081.68 | 5.90 | 6.85 | ν4 | 3210.99 | 3080.29 | 5.64 | 6.38 |
| δ5 | 1641.55 | 1603.96 | 140.11 | 79.54 | δ5 | 1639.56 | 1588.56 | 126.55 | 104.95 |
| ν6 | 1563.81 | 1526.89 | 107.80 | 2.45 | ν6 | 1559.63 | 1520.77 | 111.16 | 95.40 |
| ν7 | 1512.80 | 1466.91 | 85.76 | 78.80 | ν7 | 1512.68 | 1469.44 | 94.83 | 79.61 |
| δ8 | 1350.74 | 1328.84 | 46.24 | 37.14 | δ8 | 1349.05 | 1325.56 | 45.75 | 39.60 |
| ν9 | 1294.83 | 1260.53 | 53.37 | 31.53 | ν 9 | 1293.17 | 1257.74 | 54.48 | 37.01 |
| δ10 | 1233.08 | 1208.96 | 15.09 | 26.42 | δ10 | 1231.75 | 1206.27 | 15.19 | 29.28 |
| δ11 | 1091.63 | 1076.88 | 3.98 | 8.35 | δ11 | 1090.95 | 1074.21 | 3.79 | 4.56 |
| δ12 | 1048.63 | 1025.64 | 44.92 | 16.43 | δ12 | 1047.58 | 1021.82 | 43.99 | 30.63 |
| δ13 | 906.33 | 893.48 | 3.67 | 2.97 | δ13 | 907.29 | 889.97 | 3.26 | 2.86 |
| δ14 | 874.27 | 862.99 | 1.75 | 3.80 | δ14 | 873.35 | 858.50 | 2.24 | 6.18 |
| ν15 | 759.14 | 746.58 | 9.40 | 13.41 | ν15 | 758.46 | 745.39 | 8.89 | 11.99 |
| δ16 | 702.44 | 696.83 | 21.06 | 21.71 | δ16 | 702.61 | 694.47 | 20.90 | 24.01 |
| ν17 | 657.74 | 645.68 | 10.55 | 2.92 | ν17 | 658.41 | 645.23 | 8.10 | 2.41 |
| δ18 | 637.75 | 588.56 | 89.07 | 25.41 | δ18 | 634.97 | 589.52 | 71.65 | 26.29 |
| δ19 | 581.60 | 559.46 | 14.60 | 1.78 | δ19 | 580.68 | 555.23 | 11.50 | 2.28 |
| δ20 | 546.67 | 503.60 | 45.34 | 1.85 | δ20 | 541.96 | 512.61 | 30.53 | 2.58 |
| δ21 | 503.41 | 381.35 | 168.02 | 279.76 | δ21 | 497.57 | 327.83 | 192.14 | 223.80 |
| δ22 | 369.36 | 367.97 | 1.58 | 5.30 | δ22 | 370.63 | 374.16 | 1.60 | 4.36 |
| δ23 | 290.11 | 303.77 | 25.05 | 6.81 | δ23 | 297.14 | 161.33 | 23.33 | 59.52 |
| δ24 | 253.76 | 214.14 | 12.74 | 31.24 | δ24 | 254.29 | 281.95 | 10.67 | 4.10 |
| ν17 + δ13 | - | 1536.62 | - | 200.99 | ν17 + δ13 | - | - | - | <100 |
| **fp1a** | | | | | **fp1a / $N_2$** | | | | |
| Mode | $E_{harm}$ | $E_{anharm}$ | $I_{harm}$ | $I_{anharm}$ | Mode | $E_{harm}$ | $E_{anharm}$ | $I_{harm}$ | $I_{anharm}$ |
| ν1 | 3576.46 | 3406.86 | 80.05 | 49.22 | ν1 | 3569.53 | 3395.79 | 83.02 | 52.07 |
| ν2 | 3201.60 | 3068.35 | 3.29 | 2.23 | ν2 | 3203.47 | 3067.88 | 3.02 | 1.80 |
| ν3 | 3150.29 | 3029.27 | 14.84 | 14.29 | ν3 | 3153.61 | 3034.41 | 13.66 | 11.04 |
| ν4 | 2669.86 | 2539.38 | 1.47 | 2.52 | ν4 | 2670.18 | 2541.21 | 1.62 | 2.55 |
| ν5 | 2226.93 | 2178.72 | 1440.90 | 1148.55 | ν5 | 2213.68 | 2167.22 | 1429.75 | 796.32 |
| ν6 | 1646.53 | 1602.97 | 44.29 | 28.54 | ν6 | 1645.37 | 1603.99 | 45.41 | 16.34 |
| ν7 | 1430.06 | 1409.46 | 17.74 | 9.92 | ν7 | 1428.45 | 1408.27 | 17.29 | 12.01 |
| δ8 | 1368.23 | 1342.69 | 32.68 | 13.01 | δ8 | 1367.69 | 1344.21 | 32.77 | 28.19 |
| δ9 | 1251.73 | 1225.04 | 3.72 | 1.27 | δ9 | 1251.19 | 1226.34 | 3.30 | 0.89 |
| δ10 | 1015.45 | 992.32 | 9.00 | 3.94 | δ10 | 1013.08 | 996.41 | 10.05 | 5.00 |
| ν11 | 934.85 | 910.37 | 9.63 | 0.63 | ν11 | 932.78 | 929.63 | 15.97 | 0.93 |
| δ12 | 930.13 | 898.14 | 2.73 | 2.79 | δ12 | 932.53 | 878.62 | 0.74 | 0.04 |
| δ13 | 905.50 | 838.35 | 431.06 | 216.49 | δ13 | 905.52 | 849.17 | 403.61 | 200.76 |

| | | | | | | | | | |
|---|---|---|---|---|---|---|---|---|---|
| δ14 | 738.85 | 722.72 | 37.48 | 33.80 | δ14 | 740.39 | 722.62 | 35.82 | 33.76 |
| ν15 | 716.61 | 700.11 | 72.76 | 43.97 | ν15 | 713.93 | 699.31 | 75.45 | 54.37 |
| δ16 | 629.64 | 619.83 | 15.46 | 14.08 | δ16 | 628.99 | 618.66 | 16.81 | 18.01 |
| δ17 | 610.47 | 603.04 | 1.15 | 1.96 | δ17 | 609.31 | 603.25 | 1.04 | 2.19 |
| δ18 | 567.58 | 542.81 | 60.10 | 54.09 | δ18 | 566.57 | 536.48 | 57.42 | 42.68 |
| δ19 | 487.41 | 480.61 | 23.64 | 20.83 | δ19 | 487.25 | 478.17 | 22.58 | 20.64 |
| δ20 | 413.91 | 383.11 | 51.21 | 73.68 | δ20 | 413.01 | 389.67 | 47.85 | 52.28 |
| δ21 | 294.39 | 231.51 | 16.47 | 15.60 | δ21 | 288.38 | 210.69 | 16.40 | 14.89 |
| δ22 | 225.39 | 219.59 | 11.20 | 10.25 | δ22 | 224.68 | 223.14 | 11.62 | 11.10 |
| δ23 | 109.22 | 104.30 | 1.07 | 3.11 | δ23 | 108.74 | 104.59 | 1.15 | 1.24 |
| δ24 | 87.46 | 76.41 | 8.80 | 8.81 | δ24 | 86.44 | 70.69 | 8.43 | 9.34 |
| | | **fp1b** | | | | | **fp1b / $N_2$** | | |
| Mode | $E_{harm}$ | $E_{anharm}$ | $I_{harm}$ | $I_{anharm}$ | Mode | $E_{harm}$ | $E_{anharm}$ | $I_{harm}$ | $I_{anharm}$ |
| ν1 | 3578.20 | 3402.50 | 80.51 | 53.04 | ν1 | 3570.98 | 3391.09 | 83.13 | 34.25 |
| ν2 | 3213.60 | 3080.21 | 3.21 | 2.84 | ν2 | 3214.92 | 3086.22 | 2.95 | 2.54 |
| ν3 | 3149.49 | 3031.39 | 17.25 | 15.02 | ν3 | 3153.66 | 3035.44 | 15.56 | 13.71 |
| ν4 | 2691.24 | 2565.52 | 0.27 | 0.64 | ν4 | 2691.50 | 2573.55 | 0.06 | 0.07 |
| ν5 | 2228.14 | 2180.70 | 1476.65 | 1152.03 | ν5 | 2213.74 | 2167.18 | 1454.39 | 1080.33 |
| ν6 | 1655.27 | 1617.66 | 54.35 | 50.57 | ν6 | 1653.50 | 1627.24 | 53.93 | 41.65 |
| ν7 | 1427.76 | 1405.08 | 18.07 | 2.46 | ν7 | 1424.85 | 1399.07 | 17.60 | 10.04 |
| δ8 | 1362.95 | 1336.10 | 45.60 | 37.09 | δ8 | 1362.07 | 1332.23 | 44.84 | 36.61 |
| δ9 | 1239.15 | 1213.20 | 0.41 | 0.15 | δ9 | 1238.62 | 1207.51 | 0.39 | 0.32 |
| δ10 | 1013.64 | 995.12 | 10.60 | 2.11 | δ10 | 1011.01 | 993.52 | 11.14 | 1.52 |
| ν11 | 961.26 | 929.15 | 4.61 | 9.11 | ν11 | 959.99 | 930.19 | 5.21 | 14.56 |
| δ12 | 919.45 | 889.37 | 6.39 | 27.01 | δ12 | 922.14 | 889.37 | 6.05 | 134.60 |
| δ13 | 905.87 | 847.43 | 439.83 | 222.81 | δ13 | 906.77 | 872.02 | 413.63 | 60.83 |
| ν14 | 741.98 | 729.25 | 54.97 | 42.22 | ν14 | 740.03 | 726.50 | 57.15 | 42.42 |
| δ15 | 732.33 | 718.64 | 39.27 | 35.20 | δ15 | 733.84 | 712.18 | 37.46 | 36.40 |
| δ16 | 631.96 | 623.56 | 26.86 | 19.79 | δ16 | 631.72 | 626.48 | 29.20 | 20.27 |
| δ17 | 598.59 | 588.79 | 3.82 | 3.10 | δ17 | 597.84 | 586.43 | 4.45 | 3.89 |
| δ18 | 564.67 | 542.90 | 61.33 | 56.70 | δ18 | 564.56 | 556.78 | 58.43 | 54.81 |
| δ19 | 487.27 | 477.39 | 24.56 | 25.41 | δ19 | 487.14 | 488.24 | 23.42 | 33.70 |
| δ20 | 413.62 | 395.02 | 56.91 | 57.58 | δ20 | 413.62 | 416.23 | 52.75 | 35.03 |
| δ21 | 209.74 | 209.03 | 8.23 | 6.83 | δ21 | 209.84 | 209.34 | 8.62 | 7.43 |
| δ22 | 193.28 | 50.74 | 10.90 | 4.78 | δ22 | 191.45 | 21.92 | 10.26 | 2.17 |
| δ23 | 100.36 | 100.99 | 1.34 | 4.69 | δ23 | 99.99 | 110.42 | 1.51 | 7.42 |
| δ24 | 84.13 | 80.17 | 12.72 | 11.32 | δ24 | 83.21 | 99.81 | 12.01 | 5.84 |
| | | **fp1c** | | | | | **fp1c / $N_2$** | | |
| Mode | $E_{harm}$ | $E_{anharm}$ | $I_{harm}$ | $I_{anharm}$ | Mode | $E_{harm}$ | $E_{anharm}$ | $I_{harm}$ | $I_{anharm}$ |
| ν1 | 3568.96 | 3394.33 | 67.68 | 48.81 | ν1 | 3561.60 | 3391.84 | 70.50 | 53.37 |
| ν2 | 3183.08 | 3047.22 | 3.71 | 5.21 | ν2 | 3182.73 | 3052.68 | 3.58 | 4.77 |
| ν3 | 3130.22 | 3006.51 | 26.28 | 19.75 | ν3 | 3136.61 | 3010.59 | 22.65 | 15.85 |
| ν4 | 2669.13 | 2552.68 | 1.55 | 1.57 | ν4 | 2669.64 | 2559.98 | 0.93 | 1.01 |
| ν5 | 2223.06 | 2176.83 | 1452.90 | 959.64 | ν5 | 2209.45 | 2162.89 | 1433.99 | 835.22 |
| ν6 | 1658.10 | 1610.02 | 15.37 | 10.44 | ν6 | 1655.88 | 1612.39 | 15.37 | 9.90 |

| | | | | | | | | | |
|---|---|---|---|---|---|---|---|---|---|
| ν7 | 1415.87 | 1403.39 | 6.77 | 4.12 | ν7 | 1413.31 | 1401.82 | 6.11 | 3.60 |
| δ8 | 1351.76 | 1321.70 | 9.60 | 6.94 | δ8 | 1352.65 | 1327.20 | 9.08 | 7.17 |
| δ9 | 1276.57 | 1256.62 | 7.68 | 7.96 | δ9 | 1276.83 | 1249.41 | 7.63 | 7.61 |
| ν10 | 1044.11 | 1020.17 | 15.21 | 11.50 | ν10 | 1042.73 | 1021.62 | 14.54 | 13.00 |
| δ11 | 943.05 | 925.33 | 28.52 | 43.15 | δ11 | 941.48 | 931.92 | 31.25 | 37.60 |
| δ12 | 935.69 | 916.37 | 45.82 | 44.73 | δ12 | 937.11 | 910.87 | 44.52 | 38.65 |
| δ13 | 920.05 | 857.08 | 472.76 | 111.61 | δ13 | 922.08 | 866.69 | 442.44 | 345.12 |
| δ14 | 833.99 | 831.92 | 1.59 | 1.98 | δ14 | 835.88 | 821.81 | 1.45 | 2.73 |
| ν15 | 801.01 | 790.06 | 44.19 | 38.45 | ν15 | 800.48 | 792.86 | 46.76 | 41.32 |
| δ16 | 643.02 | 633.39 | 24.59 | 21.52 | δ16 | 642.58 | 631.48 | 27.06 | 23.60 |
| δ17 | 565.96 | 545.28 | 61.54 | 49.75 | δ17 | 566.51 | 555.88 | 58.31 | 39.72 |
| δ18 | 459.41 | 452.64 | 58.93 | 52.99 | δ18 | 459.08 | 466.11 | 54.13 | 41.22 |
| δ19 | 378.91 | 375.67 | 0.30 | 0.26 | δ19 | 378.79 | 372.18 | 0.29 | 0.20 |
| δ20 | 305.50 | 298.37 | 4.81 | 0.30 | δ20 | 305.44 | 306.46 | 4.66 | 1.08 |
| δ21 | 280.10 | 322.62 | 9.35 | 14.65 | δ21 | 282.43 | 325.72 | 8.74 | 11.94 |
| δ22 | 223.82 | 191.63 | 8.72 | 5.98 | δ22 | 227.96 | 173.03 | 8.38 | 6.04 |
| δ23 | 109.77 | 115.51 | 3.19 | 3.43 | δ23 | 109.21 | 115.81 | 3.40 | 3.99 |
| δ24 | 84.85 | 115.97 | 0.97 | 0.48 | δ24 | 85.31 | 91.12 | 0.96 | 0.55 |
| δ11 + δ9 | - | 2178.34 | - | 197.57 | δ11+ δ9 | - | - | - | <100 |
| ν15 + ν7 | - | 2186.69 | - | 202.03 | ν15 + ν7 | - | 2191.03 | - | 421.48 |
| δ24 + δ14 | - | 954.58 | - | 398.34 | δ24 + δ14 | - | - | - | <100 |

| | **fp1d** | | | | | **fp1d / $N_2$** | | | |
|---|---|---|---|---|---|---|---|---|---|
| Mode | $E_{harm}$ | $E_{anharm}$ | $I_{harm}$ | $I_{anharm}$ | Mode | $E_{harm}$ | $E_{anharm}$ | $I_{harm}$ | $I_{anharm}$ |
| ν1 | 3577.43 | 3402.05 | 81.46 | 55.02 | ν1 | 3569.57 | 3384.06 | 82.59 | 59.00 |
| ν2 | 3182.35 | 3049.20 | 5.64 | 8.31 | ν2 | 3184.56 | 3052.10 | 4.96 | 7.37 |
| ν3 | 3136.74 | 3011.50 | 17.59 | 13.36 | ν3 | 3139.83 | 3013.67 | 16.11 | 13.93 |
| ν4 | 2668.85 | 2533.59 | 0.65 | 1.37 | ν4 | 2672.53 | 2560.58 | 0.21 | 0.31 |
| ν5 | 2224.45 | 2176.38 | 1509.86 | 1112.84 | ν5 | 2210.06 | 2162.60 | 1480.55 | 1243.36 |
| ν6 | 1658.80 | 1606.14 | 37.28 | 28.45 | ν6 | 1657.22 | 1616.29 | 33.45 | 7.47 |
| ν7 | 1416.72 | 1402.36 | 10.63 | 8.43 | ν7 | 1413.81 | 1398.74 | 9.80 | 7.50 |
| δ8 | 1349.14 | 1321.83 | 4.26 | 3.83 | δ8 | 1350.49 | 1320.27 | 4.53 | 5.14 |
| δ9 | 1254.87 | 1233.81 | 19.41 | 15.59 | δ9 | 1254.76 | 1230.26 | 18.78 | 8.06 |
| ν10 | 1034.61 | 1007.40 | 22.98 | 8.61 | ν10 | 1034.07 | 1007.84 | 21.92 | 6.98 |
| δ11 | 950.57 | 935.89 | 52.24 | 56.41 | δ11 | 949.28 | 933.62 | 50.40 | 24.48 |
| δ12 | 935.25 | 901.99 | 6.41 | 35.91 | δ12 | 934.29 | 892.90 | 9.79 | 17.41 |
| δ13 | 906.02 | 826.01 | 478.65 | 262.50 | δ13 | 908.54 | 858.50 | 446.36 | 302.10 |
| δ14 | 847.58 | 836.92 | 5.54 | 16.33 | δ14 | 845.99 | 833.12 | 6.53 | 30.84 |
| ν15 | 824.65 | 806.78 | 30.27 | 14.42 | ν15 | 824.87 | 791.54 | 33.83 | 6.00 |
| δ16 | 643.81 | 631.35 | 26.69 | 24.25 | δ16 | 643.53 | 635.86 | 29.04 | 24.00 |
| δ17 | 562.57 | 541.76 | 57.56 | 38.12 | δ17 | 563.13 | 548.31 | 55.62 | 24.16 |
| δ18 | 458.28 | 435.07 | 64.63 | 47.75 | δ18 | 459.34 | 418.17 | 58.71 | 60.35 |
| δ19 | 378.26 | 373.27 | 2.03 | 2.29 | δ19 | 377.77 | 369.59 | 1.63 | 4.03 |
| δ20 | 308.35 | 293.85 | 15.68 | 5.37 | δ20 | 306.95 | 295.16 | 13.98 | 9.53 |
| δ21 | 250.77 | 228.27 | 16.13 | 19.09 | δ21 | 252.94 | 258.49 | 14.79 | 16.32 |
| δ22 | 180.89 | 18.70 | 48.76 | 14.22 | δ22 | 168.01 | 24.16 | 43.08 | 33.78 |

| | | | | | | | | | |
|---|---|---|---|---|---|---|---|---|---|
| δ23 | 106.27 | 89.21 | 7.42 | 7.20 | δ23 | 105.01 | 127.80 | 9.08 | 24.81 |
| δ24 | 84.20 | 57.66 | 3.46 | 24.45 | δ24 | 84.22 | 50.84 | 3.55 | 0.19 |
| δ17 + ν6 | - | 2150.88 | - | 183.40 | δ17 + ν6 | - | - | - | <100 |
| | | **fp2a** | | | | | **fp2a / $N_2$** | | |
| Mode | $E_{harm}$ | $E_{anharm}$ | $I_{harm}$ | $I_{anharm}$ | Mode | $E_{harm}$ | $E_{anharm}$ | $I_{harm}$ | $I_{anharm}$ |
| ν1 | 3584.00 | 3412.74 | 86.51 | 68.02 | ν1 | 3575.85 | 3402.95 | 84.25 | 66.55 |
| ν2 | 3088.76 | 2956.74 | 13.46 | 29.12 | ν2 | 3093.03 | 2955.14 | 12.36 | 27.49 |
| ν3 | 3067.20 | 2914.11 | 3.09 | 1.40 | ν3 | 3071.00 | 2920.02 | 3.10 | 0.74 |
| ν4 | 2995.21 | 2855.77 | 19.39 | 10.06 | ν4 | 2997.78 | 2865.00 | 17.80 | 9.47 |
| ν5 | 2230.36 | 2182.91 | 1148.28 | 659.58 | ν5 | 2217.78 | 2171.90 | 1095.36 | 950.79 |
| δ6 | 1465.88 | 1412.02 | 23.96 | 24.57 | δ6 | 1461.05 | 1416.21 | 23.34 | 25.15 |
| ν7 | 1415.99 | 1389.60 | 10.86 | 13.83 | ν7 | 1415.15 | 1384.88 | 9.78 | 18.42 |
| δ8 | 1394.28 | 1363.31 | 11.44 | 5.28 | δ8 | 1392.97 | 1364.54 | 10.75 | 8.28 |
| δ9 | 1312.51 | 1278.16 | 80.88 | 69.06 | δ9 | 1311.62 | 1279.74 | 77.96 | 70.19 |
| δ10 | 1228.59 | 1204.28 | 0.10 | 0.21 | δ10 | 1230.49 | 1208.84 | 0.15 | 0.73 |
| ν11 | 1146.94 | 1123.32 | 37.64 | 46.57 | ν11 | 1144.88 | 1118.53 | 38.07 | 47.36 |
| δ12 | 1031.60 | 1002.15 | 28.09 | 20.30 | δ12 | 1030.10 | 1002.48 | 27.35 | 15.55 |
| ν13 | 968.36 | 954.11 | 12.45 | 20.63 | ν13 | 968.68 | 949.06 | 11.07 | 16.95 |
| δ14 | 932.57 | 868.83 | 276.78 | 87.14 | δ14 | 936.18 | 894.58 | 274.77 | 59.47 |
| ν15 | 920.25 | 890.94 | 53.52 | 804.08 | ν15 | 919.36 | 885.11 | 33.18 | 109.98 |
| δ16 | 727.02 | 688.90 | 7.93 | 5.83 | δ16 | 724.11 | 682.73 | 7.65 | 7.28 |
| δ17 | 650.99 | 636.91 | 23.36 | 26.15 | δ17 | 650.74 | 638.46 | 24.07 | 22.67 |
| δ18 | 582.50 | 550.83 | 58.66 | 55.75 | δ18 | 582.01 | 550.24 | 55.42 | 53.69 |
| δ19 | 480.14 | 455.49 | 66.39 | 66.68 | δ19 | 479.94 | 437.34 | 61.27 | 50.92 |
| δ20 | 374.21 | 363.07 | 1.72 | 1.13 | δ20 | 373.60 | 362.95 | 1.60 | 1.16 |
| δ21 | 314.80 | 303.96 | 28.01 | 27.24 | δ21 | 313.49 | 302.22 | 26.78 | 26.76 |
| δ22 | 172.60 | 138.67 | 7.95 | 11.32 | δ22 | 170.35 | 159.13 | 8.00 | 11.87 |
| δ23 | 85.81 | 55.37 | 7.14 | 4.27 | δ23 | 84.68 | 44.08 | 7.10 | 4.09 |
| δ24 | 21.61 | 12.47 | 3.37 | 6.28 | δ24 | 21.82 | 7.32 | 3.21 | 3.12 |
| ν15 + δ9 | - | 2168.01 | - | 384.98 | ν15 + δ9 | - | - | - | <100 |
| | | **fp2b** | | | | | **fp2b / $N_2$** | | |
| Mode | $E_{harm}$ | $E_{anharm}$ | $I_{harm}$ | $I_{anharm}$ | Mode | $E_{harm}$ | $E_{anharm}$ | $I_{harm}$ | $I_{anharm}$ |
| ν1 | 3595.54 | 3421.62 | 87.36 | 70.07 | ν1 | 3583.89 | 3411.77 | 78.50 | 65.20 |
| ν2 | 3059.95 | 2915.42 | 35.60 | 52.94 | ν2 | 3078.08 | 2940.67 | 23.73 | 30.91 |
| ν3 | 3026.93 | 2884.99 | 8.69 | 10.99 | ν3 | 3035.45 | 2897.39 | 8.07 | 15.80 |
| ν4 | 2975.65 | 2812.39 | 21.44 | 9.47 | ν4 | 2978.75 | 2833.27 | 16.50 | 11.53 |
| ν5 | 2229.76 | 2188.24 | 927.97 | 637.99 | ν5 | 2209.41 | 2176.79 | 860.36 | 347.09 |
| δ6 | 1463.24 | 1426.22 | 8.67 | 3.17 | δ6 | 1458.55 | 1422.94 | 7.53 | 3.61 |
| ν7 | 1429.54 | 1404.90 | 12.59 | 6.10 | ν7 | 1422.96 | 1382.57 | 9.15 | 9.97 |
| δ8 | 1398.37 | 1363.52 | 42.74 | 28.08 | δ8 | 1394.37 | 1359.01 | 36.32 | 20.00 |
| δ9 | 1323.42 | 1287.58 | 19.44 | 12.65 | δ9 | 1322.63 | 1287.47 | 19.75 | 17.07 |
| δ10 | 1253.72 | 1222.85 | 6.11 | 6.30 | δ10 | 1251.22 | 1217.94 | 5.54 | 5.59 |
| ν11 | 1149.91 | 1122.94 | 37.78 | 37.06 | ν11 | 1144.00 | 1118.38 | 42.32 | 38.28 |
| δ12 | 997.27 | 973.35 | 19.87 | 17.50 | δ12 | 996.91 | 975.48 | 10.27 | 4.27 |
| ν13 | 962.42 | 943.49 | 23.14 | 10.95 | ν13 | 963.85 | 943.63 | 48.98 | 36.89 |

| | | | | | | | | | |
|---|---|---|---|---|---|---|---|---|---|
| δ14 | 905.00 | 870.26 | 234.20 | 101.27 | δ14 | 924.59 | 854.07 | 266.14 | 181.01 |
| ν15 | 790.75 | 769.23 | 14.91 | 18.65 | ν15 | 792.95 | 776.16 | 7.87 | 8.47 |
| δ16 | 725.30 | 713.44 | 10.02 | 15.88 | δ16 | 732.99 | 720.13 | 11.10 | 13.05 |
| δ17 | 690.84 | 673.83 | 13.17 | 2.69 | δ17 | 693.00 | 674.03 | 40.97 | 32.46 |
| δ18 | 594.66 | 573.91 | 23.02 | 39.05 | δ18 | 605.26 | 602.51 | 17.46 | 13.32 |
| δ19 | 527.07 | 510.86 | 43.63 | 21.71 | δ19 | 528.95 | 522.50 | 18.43 | 17.65 |
| δ20 | 432.88 | 412.90 | 88.13 | 55.82 | δ20 | 446.60 | 463.52 | 62.99 | 56.75 |
| δ21 | 250.68 | 265.95 | 13.41 | 10.37 | δ21 | 252.83 | 258.93 | 5.71 | 5.70 |
| δ22 | 179.64 | 164.83 | 5.30 | 9.39 | δ22 | 194.02 | 189.75 | 11.81 | 16.78 |
| δ23 | 121.46 | 108.40 | 2.58 | 0.45 | δ23 | 130.15 | 120.65 | 8.57 | 5.47 |
| δ24 | 47.58 | 32.68 | 11.45 | 15.16 | δ24 | 71.24 | 73.58 | 9.93 | 5.27 |
| | | **fp3** | | | | | **fp3 / $N_2$** | | |
| Mode | $E_{harm}$ | $E_{anharm}$ | $I_{harm}$ | $I_{anharm}$ | Mode | $E_{harm}$ | $E_{anharm}$ | $I_{harm}$ | $I_{anharm}$ |
| ν1 | 3585.02 | 3412.02 | 92.58 | 67.09 | ν1 | 3577.44 | 3410.87 | 92.05 | 78.67 |
| ν2 | 3208.03 | 3058.57 | 1.12 | 2.08 | ν2 | 3211.31 | 3061.39 | 0.76 | 1.59 |
| ν3 | 3128.84 | 2988.29 | 8.52 | 6.37 | ν3 | 3134.18 | 2997.32 | 6.95 | 4.10 |
| ν4 | 3115.71 | 2998.88 | 9.80 | 17.23 | ν4 | 3118.31 | 2997.28 | 8.52 | 12.00 |
| ν5 | 2224.53 | 2173.41 | 1258.86 | 817.87 | ν5 | 2210.05 | 2165.40 | 1222.10 | 592.98 |
| δ6 | 1479.26 | 1437.37 | 29.04 | 20.26 | δ6 | 1476.95 | 1432.93 | 26.80 | 16.76 |
| ν7 | 1432.08 | 1404.73 | 74.95 | 55.68 | ν7 | 1429.51 | 1403.65 | 73.28 | 49.80 |
| δ8 | 1354.61 | 1330.49 | 37.55 | 42.80 | δ8 | 1354.23 | 1330.72 | 34.43 | 42.17 |
| ν9 | 1171.32 | 1136.36 | 15.71 | 10.99 | ν9 | 1171.59 | 1135.24 | 15.27 | 10.65 |
| δ10 | 1135.64 | 1110.03 | 6.87 | 5.90 | δ10 | 1135.41 | 1108.28 | 7.41 | 6.53 |
| δ11 | 1075.93 | 1053.75 | 7.78 | 3.18 | δ11 | 1075.91 | 1052.95 | 7.32 | 5.34 |
| δ12 | 993.38 | 971.25 | 12.57 | 11.89 | δ12 | 992.95 | 972.22 | 12.75 | 12.12 |
| δ13 | 924.29 | 910.17 | 3.39 | 83.08 | δ13 | 923.97 | 907.29 | 3.81 | 23.99 |
| δ14 | 910.43 | 846.15 | 348.93 | 153.19 | δ14 | 911.34 | 864.67 | 330.11 | 207.37 |
| δ15 | 848.45 | 826.74 | 16.78 | 20.75 | δ15 | 849.22 | 825.34 | 13.76 | 15.75 |
| ν16 | 677.89 | 661.04 | 25.12 | 27.22 | ν16 | 675.57 | 660.34 | 26.02 | 30.39 |
| ν17 | 641.12 | 625.62 | 50.72 | 54.04 | ν17 | 637.39 | 626.44 | 47.88 | 48.75 |
| ν18 | 613.60 | 600.12 | 36.40 | 22.04 | ν18 | 611.25 | 597.10 | 35.65 | 3.98 |
| δ19 | 558.66 | 541.58 | 85.47 | 81.92 | δ19 | 554.78 | 540.69 | 90.66 | 93.81 |
| δ20 | 462.97 | 450.97 | 51.08 | 23.01 | δ20 | 464.05 | 469.11 | 45.70 | 44.16 |
| δ21 | 415.03 | 409.75 | 14.22 | 14.29 | δ21 | 414.29 | 407.65 | 14.52 | 9.09 |
| δ22 | 305.07 | 298.90 | 21.89 | 24.83 | δ22 | 304.70 | 303.60 | 19.68 | 17.78 |
| δ23 | 148.87 | 144.20 | 4.05 | 4.05 | δ23 | 149.82 | 143.36 | 4.17 | 4.16 |
| δ24 | 41.96 | 35.69 | 5.46 | 5.91 | δ24 | 45.34 | 44.67 | 5.01 | 4.87 |
| δ14 + δ8 | - | 2191.21 | - | 121.76 | - | - | - | - | - |
| δ15 + ν7 | - | 2227.84 | - | 126.76 | - | - | - | - | - |
| | | | | | δ15 + δ8 | - | 2149.36 | - | 239.49 |
| | | **fp4** | | | | | **fp4 / $N_2$** | | |
| Mode | $E_{harm}$ | $E_{anharm}$ | $I_{harm}$ | $I_{anharm}$ | Mode | $E_{harm}$ | $E_{anharm}$ | $I_{harm}$ | $I_{anharm}$ |
| ν1 | 3627.79 | 3460.13 | 171.26 | 127.31 | ν1 | 3624.30 | 3451.85 | 175.38 | 116.78 |
| ν2 | 3158.97 | 3016.57 | 4.04 | 5.51 | ν2 | 3159.78 | 3015.65 | 4.12 | 4.51 |
| ν3 | 3074.11 | 2930.69 | 4.06 | 5.31 | ν3 | 3074.68 | 2931.43 | 3.43 | 4.27 |

| | | | | | | | | | |
|---|---|---|---|---|---|---|---|---|---|
| ν4 | 3024.69 | 2918.12 | 3.96 | 3.29 | ν4 | 3025.19 | 2915.46 | 3.14 | 2.61 |
| ν5 | 2216.75 | 2180.92 | 1271.11 | 965.03 | ν5 | 2203.37 | 2170.09 | 1243.43 | 911.54 |
| δ6 | 1477.05 | 1431.20 | 3.01 | 7.47 | δ6 | 1473.62 | 1427.96 | 2.36 | 3.64 |
| δ7 | 1473.66 | 1423.62 | 6.90 | 7.48 | δ7 | 1469.66 | 1417.05 | 7.43 | 12.83 |
| ν8 | 1442.32 | 1417.83 | 210.96 | 99.33 | ν8 | 1441.53 | 1414.06 | 219.62 | 113.74 |
| δ9 | 1398.68 | 1364.43 | 8.88 | 8.54 | δ9 | 1396.48 | 1363.37 | 7.89 | 8.00 |
| ν10 | 1238.12 | 1212.71 | 197.52 | 164.05 | ν10 | 1230.41 | 1206.74 | 208.08 | 96.64 |
| ν11 | 1158.58 | 1123.91 | 61.03 | 40.30 | ν11 | 1159.09 | 1126.53 | 59.81 | 44.86 |
| δ12 | 1028.47 | 1002.98 | 0.29 | 0.17 | δ12 | 1026.84 | 1001.06 | 0.33 | 0.30 |
| δ13 | 946.78 | 926.52 | 92.20 | 78.74 | δ13 | 946.88 | 927.38 | 91.09 | 78.34 |
| δ14 | 821.27 | 772.51 | 299.83 | 139.69 | δ14 | 808.55 | 755.22 | 279.71 | 166.86 |
| ν15 | 714.74 | 701.10 | 37.75 | 49.70 | ν15 | 712.78 | 700.58 | 44.51 | 94.80 |
| δ16 | 613.27 | 602.26 | 13.41 | 15.68 | δ16 | 613.03 | 602.92 | 12.21 | 13.89 |
| δ17 | 557.84 | 548.43 | 132.60 | 133.75 | δ17 | 554.10 | 552.43 | 129.36 | 175.43 |
| δ18 | 515.48 | 504.43 | 42.02 | 0.98 | δ18 | 514.37 | 498.20 | 58.76 | 2.95 |
| δ19 | 444.24 | 446.07 | 12.33 | 0.50 | δ19 | 443.04 | 439.50 | 11.46 | 2.52 |
| δ20 | 362.82 | 356.83 | 1.25 | 1.06 | δ20 | 363.06 | 358.94 | 1.09 | 3.87 |
| δ21 | 356.69 | 310.79 | 56.18 | 55.63 | δ21 | 352.88 | 330.48 | 51.64 | 47.92 |
| δ22 | 136.48 | 171.25 | 0.53 | 15.01 | δ22 | 135.46 | 173.37 | 0.53 | 15.07 |
| δ23 | 118.64 | 79.05 | 4.22 | 1.09 | δ23 | 118.68 | 74.88 | 4.02 | 0.52 |
| δ24 | 70.82 | 46.59 | 5.94 | 3.33 | δ24 | 66.94 | 37.63 | 6.24 | 2.42 |

| | **fp14a** | | | | | **fp14a / $N_2$** | | | |
|---|---|---|---|---|---|---|---|---|---|
| Mode | $E_{harm}$ | $E_{anharm}$ | $I_{harm}$ | $I_{anharm}$ | Mode | $E_{harm}$ | $E_{anharm}$ | $I_{harm}$ | $I_{anharm}$ |
| ν1 | 3709.37 | 3509.24 | 53.16 | 146.33 | ν1 | 3713.26 | 3503.22 | 56.88 | 203.35 |
| ν2 | 3582.19 | 3408.44 | 81.48 | 110.86 | ν2 | 3585.01 | 3403.04 | 89.38 | 141.69 |
| ν3 | 3476.33 | 3344.89 | 118.82 | 97.88 | ν3 | 3470.86 | 3339.62 | 116.53 | 119.15 |
| ν4 | 2619.00 | 2487.12 | 47.44 | 37.61 | ν4 | 2623.63 | 2495.27 | 45.61 | 51.45 |
| ν5 | 2190.69 | 2130.52 | 164.96 | 46.36 | ν5 | 2185.08 | 2161.99 | 185.82 | 114.72 |
| ν6 | 1662.79 | 1622.90 | 613.51 | 567.06 | ν6 | 1652.25 | 1614.57 | 594.13 | 706.56 |
| δ7 | 1613.68 | 1580.36 | 80.71 | 24.19 | δ7 | 1609.14 | 1558.83 | 140.11 | 39.53 |
| ν8 | 1319.33 | 1293.79 | 154.34 | 104.79 | ν8 | 1322.41 | 1292.16 | 148.81 | 156.26 |
| ν9 | 1230.69 | 1206.42 | 20.01 | 13.95 | ν9 | 1228.80 | 1200.72 | 28.44 | 31.21 |
| δ10 | 972.26 | 950.28 | 42.20 | 20.51 | δ10 | 968.74 | 942.75 | 40.89 | 12.66 |
| δ11 | 950.04 | 924.90 | 21.11 | 7.39 | δ11 | 949.41 | 899.30 | 19.69 | 25.06 |
| δ12 | 641.09 | 651.29 | 20.82 | 7.04 | δ12 | 640.21 | 645.32 | 18.91 | 13.13 |
| δ13 | 624.03 | 601.81 | 7.22 | 5.10 | δ13 | 624.07 | 611.34 | 4.75 | 18.93 |
| ν14 | 618.55 | 611.99 | 10.23 | 16.52 | ν14 | 618.70 | 606.49 | 9.99 | 22.95 |
| δ15 | 567.59 | 543.90 | 45.03 | 52.03 | δ15 | 566.59 | 544.93 | 43.02 | 65.80 |
| δ16 | 558.15 | 527.01 | 57.62 | 47.72 | δ16 | 552.05 | 519.13 | 49.00 | 92.33 |
| δ17 | 512.49 | 635.38 | 3.65 | 14.08 | δ17 | 511.41 | 660.70 | 7.51 | 23.89 |
| δ18 | 464.40 | 300.52 | 30.94 | 588.12 | δ18 | 467.15 | 286.84 | 21.26 | 546.61 |
| δ19 | 413.11 | 413.90 | 0.37 | 39.75 | δ19 | 413.79 | 405.55 | 0.35 | 7.08 |
| δ20 | 366.32 | 366.32 | 13.68 | 13.68 | δ20 | 366.92 | 366.92 | 9.21 | 11.45 |
| δ21 | 325.21 | 325.21 | 140.65 | 140.65 | δ21 | 285.92 | 285.92 | 59.27 | 73.70 |
| δ22 | 238.76 | 238.76 | 91.20 | 91.20 | δ22 | 216.18 | 216.18 | 156.67 | 194.80 |

| | | | | | | | | | |
|---|---|---|---|---|---|---|---|---|---|
| δ23 | 161.33 | 161.33 | 1.61 | 1.61 | δ23 | 156.16 | 156.16 | 1.56 | 1.94 |
| δ24 | 145.06 | 145.06 | 0.24 | 0.24 | δ24 | 145.08 | 145.08 | 0.65 | 0.81 |
| | | **fp14b** | | | | | **fp14b / $N_2$** | | |
| Mode | $E_{harm}$ | $E_{anharm}$ | $I_{harm}$ | $I_{anharm}$ | Mode | $E_{harm}$ | $E_{anharm}$ | $I_{harm}$ | $I_{anharm}$ |
| ν1 | 3687.09 | 3533.73 | 36.87 | 32.18 | ν1 | 3695.75 | 3545.03 | 43.36 | 40.79 |
| ν2 | 3562.87 | 3418.44 | 54.01 | 49.52 | ν2 | 3569.11 | 3424.93 | 63.33 | 59.56 |
| ν3 | 3478.35 | 3350.46 | 112.20 | 97.93 | ν3 | 3473.14 | 3344.78 | 111.00 | 98.10 |
| ν4 | 2677.83 | 2563.23 | 1.15 | 2.47 | ν4 | 2679.84 | 2566.14 | 0.72 | 1.26 |
| ν5 | 2197.36 | 2170.59 | 145.08 | 79.40 | ν5 | 2191.07 | 2127.64 | 170.68 | 61.81 |
| ν6 | 1671.74 | 1633.33 | 573.22 | 547.60 | ν6 | 1658.34 | 1621.72 | 581.79 | 459.65 |
| δ7 | 1620.96 | 1581.08 | 57.69 | 35.52 | δ7 | 1617.40 | 1580.46 | 98.18 | 164.04 |
| ν8 | 1312.66 | 1293.15 | 149.87 | 117.99 | ν8 | 1318.46 | 1298.23 | 147.07 | 117.33 |
| δ9 | 1225.39 | 1193.01 | 8.89 | 6.45 | δ9 | 1224.25 | 1195.70 | 15.94 | 4.36 |
| ν10 | 982.52 | 950.83 | 56.17 | 21.85 | ν10 | 980.44 | 954.72 | 52.82 | 9.36 |
| δ11 | 936.62 | 925.44 | 15.02 | 9.07 | δ11 | 939.11 | 894.53 | 18.41 | 6.00 |
| ν12 | 636.20 | 619.95 | 26.76 | 28.16 | ν12 | 635.80 | 625.65 | 25.99 | 33.58 |
| δ13 | 626.81 | 571.24 | 45.33 | 79.82 | δ13 | 624.34 | 593.10 | 22.02 | 16.60 |
| δ14 | 613.41 | 600.44 | 3.33 | 16.43 | δ14 | 614.89 | 603.32 | 1.96 | 8.67 |
| δ15 | 578.60 | 560.66 | 65.21 | 61.92 | δ15 | 567.83 | 544.74 | 54.62 | 26.70 |
| δ16 | 564.68 | 548.65 | 42.82 | 47.22 | δ16 | 563.08 | 563.05 | 43.65 | 35.44 |
| δ17 | 526.33 | 515.66 | 38.47 | 0.84 | δ17 | 518.49 | 530.59 | 6.24 | 14.08 |
| δ18 | 481.93 | 350.62 | 118.06 | 67.85 | δ18 | 465.26 | 308.15 | 96.45 | 53.42 |
| δ19 | 412.93 | 408.17 | 1.06 | 1.70 | δ19 | 414.71 | 411.64 | 0.86 | 4.11 |
| δ20 | 380.21 | 287.82 | 112.18 | 286.52 | δ20 | 365.92 | 367.48 | 14.94 | 2.35 |
| δ21 | 364.83 | 357.85 | 0.74 | 6.50 | δ21 | 359.25 | 158.65 | 149.33 | 255.46 |
| δ22 | 232.49 | 218.00 | 13.49 | 16.73 | δ22 | 220.14 | 275.24 | 14.68 | 0.95 |
| δ23 | 158.63 | 123.21 | 11.77 | 10.12 | δ23 | 154.89 | 99.24 | 10.54 | 6.55 |
| δ24 | 117.79 | 111.87 | 2.41 | 3.07 | δ24 | 119.92 | 121.47 | 2.09 | 3.61 |
| | | **fp14c** | | | | | **fp14c / $N_2$** | | |
| Mode | $E_{harm}$ | $E_{anharm}$ | $I_{harm}$ | $I_{anharm}$ | Mode | $E_{harm}$ | $E_{anharm}$ | $I_{harm}$ | $I_{anharm}$ |
| ν1 | 3689.21 | 3534.50 | 56.60 | 48.36 | ν1 | 3697.13 | 3521.69 | 63.51 | 111.43 |
| ν2 | 3541.08 | 3390.47 | 34.64 | 34.46 | ν2 | 3549.84 | 3379.15 | 38.88 | 130.80 |
| ν3 | 3474.94 | 3346.68 | 119.39 | 101.83 | ν3 | 3469.66 | 3337.89 | 116.77 | 125.28 |
| ν4 | 2676.02 | 2551.45 | 1.08 | 2.24 | ν4 | 2679.63 | 2599.18 | 0.50 | 0.56 |
| ν5 | 2185.24 | 2163.18 | 182.02 | 95.35 | ν5 | 2180.12 | 2134.56 | 203.42 | 208.16 |
| ν6 | 1657.74 | 1609.92 | 401.62 | 286.20 | ν6 | 1648.32 | 1641.48 | 360.12 | 514.26 |
| δ7 | 1608.79 | 1566.25 | 241.31 | 154.84 | δ7 | 1600.29 | 1615.63 | 316.79 | 191.26 |
| ν8 | 1323.41 | 1300.70 | 139.61 | 54.11 | ν8 | 1328.51 | 1287.51 | 149.30 | 74.78 |
| δ9 | 1180.79 | 1162.32 | 3.88 | 1.75 | δ9 | 1177.96 | 1260.27 | 2.32 | 0.91 |
| ν10 | 999.25 | 979.33 | 0.65 | 3.51 | ν10 | 997.48 | 986.53 | 1.15 | 5.78 |
| δ11 | 954.10 | 922.24 | 19.28 | 15.06 | δ11 | 951.42 | 1060.91 | 21.96 | 18.46 |
| ν12 | 796.68 | 775.85 | 30.90 | 22.48 | ν12 | 795.82 | 785.79 | 31.39 | 17.51 |
| δ13 | 669.94 | 656.93 | 3.02 | 0.73 | δ13 | 669.98 | 666.72 | 1.09 | 35.62 |
| δ14 | 572.85 | 552.42 | 64.78 | 87.74 | δ14 | 568.05 | 568.87 | 56.58 | 104.24 |
| δ15 | 568.39 | 562.88 | 65.83 | 73.31 | δ15 | 563.90 | 527.96 | 60.31 | 162.15 |

| | | | | | | | | | |
|---|---|---|---|---|---|---|---|---|---|
| δ16 | 531.07 | 504.00 | 6.16 | 22.10 | δ16 | 532.64 | 496.39 | 5.64 | 21.02 |
| δ17 | 495.07 | 368.31 | 16.67 | 46.10 | δ17 | 491.61 | 384.29 | 0.32 | 545.22 |
| δ18 | 481.87 | 504.45 | 25.13 | 12.30 | δ18 | 475.12 | 617.78 | 18.11 | 180.07 |
| δ19 | 433.70 | 429.16 | 6.90 | 12.27 | δ19 | 432.97 | 544.41 | 3.87 | 150.73 |
| δ20 | 354.88 | 126.11 | 145.11 | 93.46 | δ20 | 329.12 | 329.12 | 2.15 | 2.67 |
| δ21 | 331.19 | 325.06 | 11.66 | 1.95 | δ21 | 302.59 | 302.59 | 166.32 | 206.80 |
| δ22 | 198.26 | 26.02 | 15.28 | 5.27 | δ22 | 175.55 | 175.55 | 9.15 | 11.37 |
| δ23 | 145.21 | 141.40 | 1.09 | 4.19 | δ23 | 143.30 | 143.30 | 1.30 | 1.61 |
| δ24 | 131.75 | 100.38 | 4.42 | 12.16 | δ24 | 118.08 | 118.08 | 7.68 | 9.55 |
| | | **fp14d** | | | | | **fp14d / $N_2$** | | |
| Mode | $E_{harm}$ | $E_{anharm}$ | $I_{harm}$ | $I_{anharm}$ | Mode | $E_{harm}$ | $E_{anharm}$ | $I_{harm}$ | $I_{anharm}$ |
| ν1 | 3704.81 | 3481.85 | 75.44 | 170.52 | ν1 | 3706.72 | 3441.31 | 79.11 | 866.96 |
| ν2 | 3562.26 | 3366.85 | 46.92 | 211.14 | ν2 | 3567.23 | 3329.35 | 51.51 | 1057.05 |
| ν3 | 3475.01 | 3342.81 | 119.37 | 104.56 | ν3 | 3469.53 | 3342.79 | 116.32 | 297.74 |
| ν4 | 2698.63 | 2578.36 | 6.78 | 5.56 | ν4 | 2698.72 | 2602.67 | 6.33 | 6.61 |
| ν5 | 2184.03 | 2120.58 | 189.83 | 68.19 | ν5 | 2178.49 | 2131.55 | 206.17 | 100.90 |
| ν6 | 1654.32 | 1614.39 | 385.44 | 570.77 | ν6 | 1646.67 | 1627.03 | 338.66 | 197.31 |
| δ7 | 1601.79 | 1564.78 | 265.48 | 111.80 | δ7 | 1594.86 | 1565.27 | 331.62 | 383.34 |
| ν8 | 1325.17 | 1285.48 | 158.28 | 81.34 | ν8 | 1326.59 | 1284.22 | 164.32 | 172.42 |
| ν9 | 1172.47 | 1174.13 | 4.11 | 1.88 | ν9 | 1171.02 | 1192.14 | 3.11 | 8.73 |
| ν10 | 994.38 | 986.75 | 1.52 | 6.56 | ν10 | 992.56 | 995.41 | 2.18 | 20.20 |
| δ11 | 911.02 | 894.82 | 23.04 | 12.95 | δ11 | 911.73 | 881.75 | 23.20 | 22.45 |
| δ12 | 796.38 | 791.94 | 32.46 | 29.10 | δ12 | 795.62 | 805.25 | 32.10 | 33.58 |
| δ13 | 668.01 | 656.47 | 0.45 | 32.13 | δ13 | 669.11 | 653.15 | 0.25 | 134.42 |
| δ14 | 571.73 | 562.76 | 63.95 | 58.42 | δ14 | 570.09 | 545.23 | 59.79 | 48.17 |
| δ15 | 563.46 | 605.96 | 65.77 | 158.94 | δ15 | 557.47 | 531.85 | 59.70 | 109.62 |
| δ16 | 528.99 | 514.42 | 4.98 | 11.04 | δ16 | 529.70 | 503.30 | 4.97 | 16.49 |
| δ17 | 493.91 | 350.76 | 7.69 | 644.18 | δ17 | 495.11 | 173.33 | 5.99 | 1635.74 |
| δ18 | 488.47 | 540.93 | 1.60 | 204.28 | δ18 | 490.05 | 744.20 | 0.42 | 1527.96 |
| δ19 | 422.68 | 419.72 | 2.10 | 2.29 | δ19 | 423.76 | 423.59 | 1.95 | 3.93 |
| δ20 | 327.90 | 327.90 | 4.88 | 4.88 | δ20 | 328.34 | 328.34 | 4.92 | 6.12 |
| δ21 | 250.72 | 250.72 | 21.21 | 21.21 | δ21 | 227.49 | 227.49 | 0.89 | 1.11 |
| δ22 | 159.77 | 159.77 | 60.88 | 60.88 | δ22 | 145.05 | 145.05 | 10.85 | 13.49 |
| δ23 | 146.21 | 146.21 | 0.61 | 0.61 | δ23 | 144.80 | 144.80 | 0.59 | 0.73 |
| δ24 | 113.33 | 113.33 | 98.74 | 98.74 | δ24 | 54.02 | 54.02 | 155.24 | 193.02 |
| | | **fp15** | | | | | **fp15 / $N_2$** | | |
| Mode | $E_{harm}$ | $E_{anharm}$ | $I_{harm}$ | $I_{anharm}$ | Mode | $E_{harm}$ | $E_{anharm}$ | $I_{harm}$ | $I_{anharm}$ |
| ν1 | 3715.22 | 3519.56 | 61.13 | 23.04 | ν1 | 3710.22 | 3523.42 | 61.21 | 70.02 |
| ν2 | 3576.11 | 3404.43 | 76.67 | 48.31 | ν2 | 3573.43 | 3402.09 | 83.12 | 97.70 |
| ν3 | 3244.18 | 3102.77 | 3.59 | 1.71 | ν3 | 3243.48 | 3099.74 | 3.87 | 3.21 |
| ν4 | 3156.12 | 3030.83 | 16.98 | 12.63 | ν4 | 3154.62 | 3026.48 | 17.14 | 15.89 |
| ν5 | 2124.76 | 2093.63 | 689.12 | 202.24 | ν5 | 2117.92 | 2067.96 | 680.26 | 5.54 |
| δ6 | 1624.74 | 1584.17 | 221.87 | 34.19 | δ6 | 1624.35 | 1557.41 | 209.83 | 204.37 |
| δ7 | 1450.17 | 1394.56 | 10.96 | 1.85 | δ7 | 1447.89 | 1400.11 | 14.83 | 19.00 |
| ν8 | 1384.58 | 1347.77 | 287.20 | 212.47 | ν8 | 1386.92 | 1339.91 | 288.30 | 156.39 |

| | | | | | | | | | |
|---|---|---|---|---|---|---|---|---|---|
| ν9 | 1304.38 | 1286.64 | 231.89 | 85.32 | ν9 | 1301.34 | 1261.51 | 241.68 | 159.62 |
| ν10 | 1218.12 | 1199.76 | 5.08 | 1.08 | ν10 | 1216.97 | 1198.15 | 7.34 | 16.07 |
| δ11 | 985.67 | 965.30 | 1.43 | 14.75 | δ11 | 983.47 | 969.18 | 1.77 | 2.05 |
| δ12 | 921.50 | 878.89 | 82.67 | 22.52 | δ12 | 917.10 | 881.37 | 87.54 | 51.25 |
| δ13 | 742.37 | 721.70 | 52.79 | 9.75 | δ13 | 744.67 | 783.67 | 55.17 | 14.30 |
| ν14 | 731.75 | 709.24 | 23.87 | 66.07 | ν14 | 731.31 | 660.30 | 18.27 | 72.33 |
| δ15 | 673.36 | 666.59 | 1.50 | 4.43 | δ15 | 671.98 | 681.09 | 2.07 | 10.30 |
| δ16 | 625.48 | 608.04 | 16.02 | 14.01 | δ16 | 625.96 | 632.10 | 14.69 | 25.86 |
| δ17 | 580.82 | 555.30 | 3.21 | 3.07 | δ17 | 581.48 | 535.25 | 2.61 | 11.83 |
| δ18 | 508.06 | 496.08 | 0.56 | 1.05 | δ18 | 510.07 | 447.88 | 0.59 | 71.41 |
| δ19 | 404.10 | 397.81 | 1.23 | 14.53 | δ19 | 404.28 | 341.95 | 1.14 | 2.61 |
| δ20 | 376.06 | 374.63 | 1.11 | 18.35 | δ20 | 375.34 | 375.34 | 1.53 | 1.91 |
| δ21 | 337.62 | 356.85 | 64.41 | 59.04 | δ21 | 349.33 | 349.33 | 144.57 | 179.76 |
| δ22 | 320.87 | 410.91 | 112.25 | 31.48 | δ22 | 330.67 | 330.67 | 17.29 | 21.49 |
| δ23 | 138.75 | 133.74 | 4.77 | 2.93 | δ23 | 139.42 | 139.42 | 4.59 | 5.70 |
| δ24 | 48.65 | 46.10 | 5.69 | 6.25 | δ24 | 45.51 | 45.51 | 6.01 | 7.48 |
| δ15 + δ7 | - | 2083.71 | - | 530.78 | δ15 + δ7 | - | 2069.41 | - | 309.92 |
| δ22 + ν9 | - | 1692.89 | - | 171.51 | δ22 + ν9 | - | - | - | <100 |
| | | **fp17a** | | | | | **fp17a / $N_2$** | | |
| Mode | $E_{harm}$ | $E_{anharm}$ | $I_{harm}$ | $I_{anharm}$ | Mode | $E_{harm}$ | $E_{anharm}$ | $I_{harm}$ | $I_{anharm}$ |
| ν1 | 3560.97 | 3382.45 | 61.53 | 47.19 | ν1 | 3558.30 | 3377.42 | 60.05 | 46.70 |
| ν2 | 3547.40 | 3376.35 | 29.83 | 23.45 | ν2 | 3544.09 | 3364.49 | 29.53 | 24.27 |
| ν3 | 3473.65 | 3347.88 | 125.98 | 111.57 | ν3 | 3468.07 | 3344.03 | 118.99 | 105.57 |
| ν4 | 2659.07 | 2533.53 | 23.01 | 22.62 | ν4 | 2659.60 | 2531.97 | 22.69 | 22.51 |
| ν5 | 2232.62 | 2191.78 | 139.78 | 72.17 | ν5 | 2228.95 | 2188.73 | 137.78 | 93.90 |
| ν6 | 1661.89 | 1630.81 | 418.44 | 283.74 | ν6 | 1651.51 | 1620.55 | 427.02 | 331.14 |
| δ7 | 1438.82 | 1405.29 | 8.96 | 9.27 | δ7 | 1440.72 | 1409.29 | 7.27 | 7.09 |
| ν8 | 1318.75 | 1271.35 | 140.33 | 120.95 | ν8 | 1319.73 | 1269.63 | 135.65 | 119.50 |
| ν9 | 1185.51 | 1154.50 | 137.39 | 97.70 | ν9 | 1186.45 | 1151.39 | 136.15 | 73.54 |
| δ10 | 1035.12 | 1004.86 | 109.01 | 89.06 | δ10 | 1037.27 | 1004.40 | 103.67 | 88.88 |
| δ11 | 919.02 | 908.01 | 8.17 | 1.68 | δ11 | 918.48 | 869.98 | 8.32 | 16.03 |
| δ12 | 776.97 | 762.64 | 34.75 | 35.60 | δ12 | 775.15 | 753.86 | 32.78 | 34.23 |
| δ13 | 727.55 | 690.38 | 35.66 | 41.23 | δ13 | 727.30 | 705.94 | 33.06 | 39.09 |
| ν14 | 627.62 | 615.42 | 15.30 | 9.02 | ν14 | 628.09 | 615.49 | 14.92 | 7.17 |
| δ15 | 621.58 | 612.02 | 19.72 | 19.04 | δ15 | 620.35 | 614.40 | 17.98 | 18.51 |
| δ16 | 601.24 | 590.32 | 13.05 | 13.43 | δ16 | 601.43 | 583.69 | 12.81 | 12.67 |
| δ17 | 492.81 | 513.19 | 56.02 | 2.28 | δ17 | 493.11 | 512.45 | 51.29 | 1.24 |
| δ18 | 439.72 | 435.62 | 83.28 | 138.82 | δ18 | 437.11 | 442.55 | 79.14 | 133.48 |
| δ19 | 425.52 | 362.33 | 11.47 | 2.15 | δ19 | 428.23 | 368.83 | 11.32 | 0.07 |
| δ20 | 416.28 | 412.94 | 19.83 | 18.87 | δ20 | 416.19 | 413.20 | 19.33 | 17.67 |
| δ21 | 345.29 | 338.52 | 5.44 | 5.35 | δ21 | 346.00 | 330.97 | 5.26 | 4.76 |
| δ22 | 195.26 | 259.22 | 21.87 | 17.01 | δ22 | 197.02 | 38.38 | 22.49 | 6.21 |
| δ23 | 160.99 | 152.74 | 0.88 | 0.65 | δ23 | 160.73 | 135.43 | 0.89 | 0.72 |
| δ24 | 76.32 | 92.64 | 0.53 | 0.04 | δ24 | 77.66 | 76.04 | 0.75 | 10.59 |
| | | **fp17b** | | | | | **fp17b / $N_2$** | | |

| Mode | $E_{harm}$ | $E_{anharm}$ | $I_{harm}$ | $I_{anharm}$ |
|---|---|---|---|---|
| ν1 | 3572.65 | 3382.26 | 63.57 | 35.28 |
| ν2 | 3567.75 | 3404.66 | 26.47 | 35.20 |
| ν3 | 3475.53 | 3349.78 | 119.66 | 106.51 |
| ν4 | 2691.96 | 2565.93 | 1.43 | 1.60 |
| ν5 | 2237.10 | 2194.61 | 128.80 | 115.65 |
| ν6 | 1670.33 | 1637.61 | 413.05 | 289.14 |
| δ7 | 1439.88 | 1406.45 | 10.82 | 9.78 |
| ν8 | 1306.21 | 1258.12 | 152.06 | 101.02 |
| ν9 | 1178.37 | 1144.90 | 142.40 | 85.48 |
| ν10 | 1033.56 | 1002.14 | 123.98 | 64.61 |
| δ11 | 962.52 | 937.95 | 24.88 | 17.60 |
| δ12 | 752.21 | 736.58 | 37.23 | 22.27 |
| δ13 | 726.47 | 691.44 | 35.99 | 40.06 |
| ν14 | 632.89 | 621.55 | 8.99 | 7.26 |
| δ15 | 616.18 | 611.03 | 22.99 | 24.01 |
| δ16 | 594.81 | 583.70 | 48.57 | 36.15 |
| δ17 | 498.30 | 511.98 | 66.25 | 25.54 |
| δ18 | 445.95 | 470.57 | 45.74 | 14.16 |
| δ19 | 437.19 | 431.54 | 12.61 | 11.76 |
| δ20 | 428.37 | 359.62 | 50.85 | 97.19 |
| δ21 | 332.75 | 324.48 | 3.64 | 3.27 |
| δ22 | 243.83 | 260.88 | 1.42 | 2.88 |
| δ23 | 136.84 | 135.77 | 0.86 | 0.77 |
| δ24 | 86.10 | 93.22 | 0.49 | 0.09 |

| Mode | $E_{harm}$ | $E_{anharm}$ | $I_{harm}$ | $I_{anharm}$ |
|---|---|---|---|---|
| ν1 | 3569.54 | 3397.90 | 62.02 | 36.39 |
| ν2 | 3564.84 | 3375.94 | 27.06 | 35.94 |
| ν3 | 3469.31 | 3343.97 | 113.72 | 101.62 |
| ν4 | 2693.03 | 2578.60 | 0.83 | 1.10 |
| ν5 | 2232.72 | 2182.08 | 129.37 | 65.04 |
| ν6 | 1660.97 | 1630.96 | 419.20 | 298.94 |
| δ7 | 1441.63 | 1413.02 | 9.10 | 6.65 |
| ν8 | 1306.16 | 1274.79 | 146.84 | 100.94 |
| ν9 | 1179.60 | 1164.04 | 140.41 | 86.22 |
| ν10 | 1035.48 | 1023.01 | 118.66 | 38.20 |
| δ11 | 963.57 | 957.27 | 24.53 | 9.66 |
| δ12 | 752.32 | 745.83 | 35.19 | 17.77 |
| δ13 | 726.55 | 702.61 | 33.35 | 40.76 |
| ν14 | 633.58 | 623.72 | 8.83 | 4.51 |
| δ15 | 615.17 | 611.67 | 20.80 | 23.09 |
| δ16 | 595.37 | 590.59 | 46.76 | 34.19 |
| δ17 | 498.22 | 440.59 | 61.04 | 23.26 |
| δ18 | 444.70 | 517.31 | 33.70 | 10.01 |
| δ19 | 438.11 | 443.45 | 12.06 | 5.08 |
| δ20 | 428.58 | 361.41 | 59.95 | 94.48 |
| δ21 | 333.96 | 329.97 | 3.58 | 7.68 |
| δ22 | 249.97 | 325.87 | 1.26 | 4.28 |
| δ23 | 137.77 | 135.53 | 0.84 | 1.02 |
| δ24 | 87.48 | 93.48 | 0.38 | 0.07 |

**fp17c**

| Mode | $E_{harm}$ | $E_{anharm}$ | $I_{harm}$ | $I_{anharm}$ |
|---|---|---|---|---|
| ν1 | 3570.05 | 3394.61 | 38.56 | 27.61 |
| ν2 | 3486.27 | 3308.33 | 6.90 | 0.46 |
| ν3 | 3472.73 | 3346.64 | 125.69 | 120.79 |
| ν4 | 2672.58 | 2546.97 | 15.11 | 15.15 |
| ν5 | 2230.42 | 2189.29 | 125.28 | 93.11 |
| ν6 | 1669.34 | 1631.65 | 378.93 | 275.08 |
| δ7 | 1451.66 | 1417.26 | 11.34 | 9.25 |
| ν8 | 1286.17 | 1238.02 | 328.38 | 223.05 |
| ν9 | 1176.86 | 1158.13 | 0.82 | 1.50 |
| δ10 | 1036.30 | 1022.51 | 92.80 | 64.93 |
| δ11 | 949.16 | 920.14 | 8.15 | 10.53 |
| δ12 | 778.13 | 761.82 | 96.25 | 59.32 |
| δ13 | 727.64 | 689.18 | 36.19 | 56.42 |
| ν14 | 641.71 | 627.00 | 30.90 | 19.77 |
| δ15 | 591.00 | 583.33 | 3.96 | 2.29 |
| δ16 | 588.29 | 575.40 | 9.12 | 7.13 |
| δ17 | 498.17 | 505.35 | 47.80 | 18.29 |
| δ18 | 431.24 | 395.16 | 23.50 | 22.94 |

**fp17c / $N_2$**

| Mode | $E_{harm}$ | $E_{anharm}$ | $I_{harm}$ | $I_{anharm}$ |
|---|---|---|---|---|
| ν1 | 3565.92 | 3391.61 | 42.59 | 31.09 |
| ν2 | 3487.86 | 3318.18 | 8.48 | 4.26 |
| ν3 | 3466.67 | 3343.08 | 118.06 | 109.00 |
| ν4 | 2673.34 | 2547.63 | 16.27 | 15.75 |
| ν5 | 2226.88 | 2171.45 | 124.93 | 67.86 |
| ν6 | 1658.65 | 1633.94 | 391.69 | 272.60 |
| δ7 | 1454.46 | 1462.40 | 11.43 | 44.41 |
| ν8 | 1289.60 | 1262.70 | 312.68 | 158.85 |
| ν9 | 1179.06 | 1161.13 | 1.63 | 1.43 |
| δ10 | 1038.57 | 1063.72 | 88.33 | 57.47 |
| δ11 | 946.58 | 949.67 | 8.07 | 6.00 |
| δ12 | 777.96 | 780.06 | 90.83 | 57.68 |
| δ13 | 727.47 | 698.03 | 33.51 | 37.28 |
| ν14 | 639.97 | 646.84 | 30.71 | 16.75 |
| δ15 | 591.93 | 602.08 | 3.33 | 0.36 |
| δ16 | 589.78 | 576.66 | 8.76 | 7.21 |
| δ17 | 498.81 | 512.03 | 44.95 | 28.41 |
| δ18 | 433.16 | 408.94 | 20.59 | 53.95 |

| Mode | $E_{harm}$ | $E_{anharm}$ | $I_{harm}$ | $I_{anharm}$ | Mode | $E_{harm}$ | $E_{anharm}$ | $I_{harm}$ | $I_{anharm}$ |
|---|---|---|---|---|---|---|---|---|---|
| δ19 | 424.30 | 420.32 | 9.19 | 6.21 | δ19 | 423.24 | 439.53 | 9.20 | 5.21 |
| δ20 | 361.93 | 349.69 | 48.67 | 63.47 | δ20 | 370.05 | 452.75 | 48.43 | 38.83 |
| δ21 | 344.48 | 338.43 | 9.40 | 8.33 | δ21 | 345.03 | 341.63 | 8.95 | 7.65 |
| δ22 | 219.88 | 288.97 | 8.90 | 10.34 | δ22 | 214.94 | 493.54 | 8.58 | 13.80 |
| δ23 | 162.39 | 156.36 | 0.57 | 0.50 | δ23 | 162.09 | 142.99 | 0.58 | 0.51 |
| δ24 | 73.49 | 89.10 | 2.55 | 1.84 | δ24 | 73.73 | 222.37 | 2.89 | 30.49 |
| δ21 + δ11 | - | - | - | <100 | δ21 + δ11 | - | 1293.92 | - | 164.33 |

| | **fp17d** | | | | | **fp17d / $N_2$** | | | |
|---|---|---|---|---|---|---|---|---|---|
| Mode | $E_{harm}$ | $E_{anharm}$ | $I_{harm}$ | $I_{anharm}$ | Mode | $E_{harm}$ | $E_{anharm}$ | $I_{harm}$ | $I_{anharm}$ |
| ν1 | 3580.23 | 3397.72 | 43.27 | 31.93 | 1(1) | 3576.88 | 3408.97 | 46.75 | 33.40 |
| ν2 | 3475.07 | 3343.18 | 74.53 | 42.05 | 2(1) | 3475.10 | 3303.90 | 4.28 | 27.26 |
| ν3 | 3473.56 | 3304.58 | 52.03 | 66.95 | 3(1) | 3468.68 | 3342.08 | 116.52 | 133.23 |
| ν4 | 2694.34 | 2572.61 | 1.99 | 2.48 | 4(1) | 2694.97 | 2574.15 | 1.88 | 1.45 |
| ν5 | 2235.71 | 2191.96 | 117.34 | 76.94 | 5(1) | 2231.40 | 2176.67 | 118.91 | 73.81 |
| ν6 | 1671.05 | 1631.94 | 373.79 | 202.20 | 6(1) | 1662.17 | 1639.96 | 382.88 | 245.19 |
| δ7 | 1450.40 | 1416.11 | 12.53 | 10.76 | 7(1) | 1454.85 | 1454.36 | 13.32 | 52.52 |
| ν8 | 1289.18 | 1243.33 | 344.08 | 272.96 | 8(1) | 1291.40 | 1269.71 | 327.92 | 311.52 |
| ν9 | 1172.97 | 1154.94 | 0.61 | 0.85 | 9(1) | 1174.97 | 1155.95 | 1.42 | 0.84 |
| ν10 | 1029.15 | 1011.49 | 75.34 | 20.87 | 10(1) | 1032.97 | 1060.17 | 72.74 | 39.83 |
| δ11 | 942.93 | 924.23 | 27.08 | 12.12 | 11(1) | 940.61 | 923.66 | 26.15 | 23.11 |
| δ12 | 772.89 | 757.64 | 88.75 | 82.67 | 12(1) | 773.64 | 794.45 | 83.90 | 61.49 |
| δ13 | 726.37 | 696.18 | 36.28 | 40.36 | 13(1) | 726.20 | 706.21 | 33.63 | 39.61 |
| ν14 | 643.64 | 632.49 | 28.69 | 28.61 | 14(1) | 643.31 | 654.16 | 28.11 | 16.69 |
| δ15 | 586.75 | 581.21 | 3.48 | 3.57 | 15(1) | 587.57 | 602.32 | 2.69 | 3.03 |
| δ16 | 586.32 | 571.73 | 33.13 | 18.99 | 16(1) | 587.44 | 568.13 | 32.67 | 23.42 |
| δ17 | 503.79 | 516.21 | 54.42 | 18.22 | 17(1) | 503.44 | 501.40 | 50.65 | 14.09 |
| δ18 | 439.55 | 437.01 | 10.31 | 8.37 | 18(1) | 439.65 | 441.86 | 10.07 | 6.43 |
| δ19 | 435.84 | 392.28 | 5.67 | 0.04 | 19(1) | 437.14 | 390.61 | 5.33 | 14.12 |
| δ20 | 376.69 | 442.88 | 88.06 | 96.88 | 20(1) | 380.79 | 470.94 | 83.96 | 129.83 |
| δ21 | 327.97 | 325.12 | 5.33 | 5.11 | 21(1) | 329.15 | 335.20 | 5.19 | 6.85 |
| δ22 | 301.87 | 290.25 | 0.10 | 12.77 | 22(1) | 300.61 | 342.17 | 0.13 | 0.50 |
| δ23 | 136.80 | 139.03 | 0.02 | 0.02 | 23(1) | 137.94 | 137.99 | 0.03 | 0.05 |
| δ24 | 87.36 | 96.06 | 0.01 | 0.74 | 24(1) | 87.50 | 161.05 | 0.04 | 0.92 |
| ν14 + ν10 | - | 1644.51 | - | 114.69 | ν14 + ν10 | - | - | - | <100 |

| | **fp17e** | | | | | **fp17e / $N_2$** | | | |
|---|---|---|---|---|---|---|---|---|---|
| Mode | $E_{harm}$ | $E_{anharm}$ | $I_{harm}$ | $I_{anharm}$ | Mode | $E_{harm}$ | $E_{anharm}$ | $I_{harm}$ | $I_{anharm}$ |
| ν1 | 3615.82 | 3443.57 | 50.51 | 38.79 | 1(1) | 3615.56 | 3432.75 | 52.58 | 43.16 |
| ν2 | 3547.91 | 3370.63 | 25.99 | 20.98 | 2(1) | 3544.30 | 3369.83 | 24.69 | 20.88 |
| ν3 | 3476.78 | 3348.48 | 110.55 | 94.73 | 3(1) | 3470.83 | 3341.79 | 104.99 | 93.32 |
| ν4 | 2662.83 | 2536.60 | 0.68 | 1.46 | 4(1) | 2665.04 | 2555.99 | 0.40 | 1.08 |
| ν5 | 2243.30 | 2194.60 | 101.50 | 73.82 | 5(1) | 2238.36 | 2191.46 | 104.09 | 88.34 |
| ν6 | 1686.29 | 1654.56 | 199.83 | 120.74 | 6(1) | 1674.05 | 1630.20 | 201.89 | 97.62 |
| δ7 | 1495.92 | 1453.46 | 169.51 | 115.13 | 7(1) | 1498.85 | 1447.04 | 163.66 | 126.61 |
| δ8 | 1249.99 | 1208.66 | 233.21 | 203.56 | 8(1) | 1256.53 | 1214.53 | 226.08 | 190.57 |
| ν9 | 1146.50 | 1115.60 | 81.02 | 59.50 | 9(1) | 1148.09 | 1119.35 | 77.83 | 42.84 |

| | | | | | | | | | |
|---|---|---|---|---|---|---|---|---|---|
| ν10 | 1011.87 | 992.64 | 24.01 | 19.13 | 10(1) | 1014.35 | 997.29 | 22.53 | 15.24 |
| δ11 | 913.05 | 883.44 | 2.51 | 1.30 | 11(1) | 912.21 | 915.80 | 2.82 | 10.02 |
| δ12 | 834.25 | 825.09 | 71.37 | 73.19 | 12(1) | 837.19 | 826.46 | 70.96 | 29.15 |
| δ13 | 806.73 | 781.57 | 77.56 | 31.56 | 13(1) | 801.30 | 782.78 | 70.85 | 35.68 |
| δ14 | 711.36 | 685.35 | 31.95 | 33.14 | 14(1) | 712.25 | 671.48 | 29.91 | 27.96 |
| δ15 | 591.26 | 586.67 | 2.34 | 1.96 | 15(1) | 597.55 | 590.77 | 2.21 | 1.56 |
| δ16 | 527.33 | 514.02 | 56.49 | 29.48 | 16(1) | 524.48 | 509.47 | 52.56 | 33.59 |
| ν17 | 469.84 | 449.03 | 22.56 | 5.65 | 17(1) | 471.34 | 459.71 | 20.31 | 7.57 |
| δ18 | 447.06 | 428.64 | 4.49 | 20.16 | 18(1) | 448.24 | 429.48 | 4.56 | 18.63 |
| δ19 | 404.95 | 479.84 | 4.78 | 2.80 | 19(1) | 405.82 | 444.12 | 1.95 | 1.76 |
| δ20 | 376.09 | 259.06 | 48.50 | 44.40 | 20(1) | 367.65 | 120.63 | 48.76 | 34.07 |
| δ21 | 319.43 | 333.22 | 13.89 | 5.70 | 21(1) | 320.97 | 351.24 | 17.17 | 5.32 |
| δ22 | 199.13 | 75.85 | 19.17 | 10.74 | 22(1) | 193.11 | 206.02 | 17.70 | 10.85 |
| δ23 | 145.04 | 136.22 | 1.49 | 1.76 | 23(1) | 146.99 | 101.99 | 1.86 | 2.14 |
| δ24 | 92.96 | 93.14 | 0.71 | 0.16 | 24(1) | 92.60 | 73.73 | 0.65 | 4.89 |

| | **fp17f** | | | | | **fp17f / $N_2$** | | | |
|---|---|---|---|---|---|---|---|---|---|
| Mode | $E_{harm}$ | $E_{anharm}$ | $I_{harm}$ | $I_{anharm}$ | Mode | $E_{harm}$ | $E_{anharm}$ | $I_{harm}$ | $I_{anharm}$ |
| ν1 | 3605.20 | 3430.04 | 54.35 | 49.72 | ν1 | 3601.68 | 3421.04 | 56.27 | 63.37 |
| ν2 | 3560.33 | 3385.40 | 23.37 | 11.34 | ν2 | 3558.97 | 3357.86 | 23.34 | 17.40 |
| ν3 | 3476.88 | 3348.83 | 112.03 | 73.15 | ν3 | 3470.73 | 3342.79 | 106.50 | 106.89 |
| ν4 | 2698.34 | 2582.27 | 0.49 | 0.63 | ν4 | 2700.84 | 2569.07 | 0.93 | 0.43 |
| ν5 | 2244.61 | 2203.71 | 106.49 | 76.88 | ν5 | 2239.58 | 2192.78 | 110.70 | 105.69 |
| ν6 | 1690.81 | 1662.01 | 207.05 | 56.69 | ν6 | 1679.21 | 1646.64 | 205.25 | 93.65 |
| δ7 | 1502.48 | 1460.77 | 187.45 | 167.34 | δ7 | 1504.75 | 1459.53 | 182.35 | 199.52 |
| δ8 | 1239.49 | 1205.73 | 255.01 | 144.37 | δ8 | 1243.85 | 1207.76 | 250.78 | 177.59 |
| ν9 | 1147.64 | 1110.66 | 83.99 | 80.65 | ν9 | 1150.45 | 1103.93 | 80.60 | 87.31 |
| ν10 | 1016.67 | 991.89 | 19.61 | 15.62 | ν10 | 1018.91 | 1001.91 | 18.11 | 21.02 |
| δ11 | 908.92 | 896.18 | 12.09 | 2.78 | δ11 | 910.76 | 874.19 | 15.27 | 8.69 |
| δ12 | 835.46 | 817.44 | 84.39 | 64.72 | δ12 | 839.22 | 800.86 | 82.17 | 92.84 |
| δ13 | 782.19 | 765.92 | 76.59 | 48.02 | δ13 | 778.19 | 766.90 | 71.64 | 49.52 |
| δ14 | 710.75 | 685.74 | 32.07 | 34.85 | δ14 | 711.84 | 683.27 | 29.97 | 41.69 |
| δ15 | 599.64 | 591.88 | 1.91 | 7.77 | δ15 | 603.94 | 600.02 | 2.17 | 6.83 |
| δ16 | 527.41 | 524.78 | 56.63 | 24.29 | δ16 | 524.22 | 526.65 | 52.67 | 41.78 |
| ν17 | 477.79 | 479.00 | 20.11 | 80.12 | ν17 | 479.94 | 468.18 | 18.14 | 69.70 |
| δ18 | 446.55 | 426.54 | 6.20 | 38.29 | δ18 | 447.49 | 426.97 | 5.83 | 33.51 |
| δ19 | 415.42 | 391.06 | 1.79 | 5.09 | δ19 | 419.06 | 394.56 | 1.37 | 10.22 |
| δ20 | 373.23 | 373.23 | 55.33 | 55.33 | δ20 | 365.78 | 365.78 | 48.90 | 60.80 |
| δ21 | 311.86 | 311.86 | 7.03 | 7.03 | δ21 | 311.41 | 311.41 | 8.33 | 10.36 |
| δ22 | 185.96 | 185.96 | 20.87 | 20.87 | δ22 | 177.22 | 177.22 | 25.01 | 31.10 |
| δ23 | 148.37 | 148.37 | 1.70 | 1.70 | δ23 | 150.91 | 150.91 | 1.53 | 1.90 |
| δ24 | 92.64 | 92.64 | 1.19 | 1.19 | δ24 | 89.45 | 89.45 | 0.39 | 0.49 |

| | **fp17g** | | | | | **fp17g / $N_2$** | | | |
|---|---|---|---|---|---|---|---|---|---|
| Mode | $E_{harm}$ | $E_{anharm}$ | $I_{harm}$ | $I_{anharm}$ | Mode | $E_{harm}$ | $E_{anharm}$ | $I_{harm}$ | $I_{anharm}$ |
| ν1 | 3605.02 | 3435.31 | 42.28 | 29.89 | ν1 | 3604.84 | 3446.36 | 46.32 | 44.96 |
| ν2 | 3478.04 | 3304.34 | 30.42 | 611.38 | ν2 | 3477.01 | 3296.00 | 22.15 | 216.38 |

| | | | | | | | | | |
|---|---|---|---|---|---|---|---|---|---|
| ν3 | 3472.66 | 3342.08 | 101.21 | 32.10 | ν3 | 3467.39 | 3340.63 | 102.57 | 52.35 |
| ν4 | 2668.43 | 2546.54 | 1.72 | 2.88 | ν4 | 2672.92 | 2544.12 | 0.92 | 0.70 |
| ν5 | 2234.57 | 2186.85 | 109.83 | 93.09 | ν5 | 2231.06 | 2184.67 | 107.76 | 110.58 |
| ν6 | 1685.47 | 1655.04 | 180.14 | 91.07 | ν6 | 1672.75 | 1642.98 | 186.99 | 111.05 |
| δ7 | 1468.26 | 1428.13 | 185.84 | 132.83 | δ7 | 1473.74 | 1428.77 | 180.17 | 202.39 |
| δ8 | 1261.10 | 1219.10 | 174.32 | 129.52 | δ8 | 1266.05 | 1226.50 | 166.60 | 120.16 |
| ν9 | 1150.04 | 1126.98 | 76.76 | 40.43 | ν9 | 1152.96 | 1124.34 | 76.77 | 39.37 |
| δ10 | 1025.21 | 1004.07 | 36.02 | 51.11 | δ10 | 1028.55 | 1000.69 | 33.62 | 39.91 |
| ν11 | 920.88 | 899.14 | 30.24 | 21.26 | ν11 | 920.65 | 914.48 | 28.81 | 34.63 |
| δ12 | 835.16 | 804.13 | 55.90 | 41.09 | δ12 | 830.27 | 812.84 | 47.56 | 109.76 |
| δ13 | 829.83 | 817.55 | 49.36 | 40.94 | δ13 | 826.08 | 807.64 | 53.94 | 118.64 |
| δ14 | 722.85 | 697.95 | 30.82 | 28.60 | δ14 | 723.48 | 712.61 | 28.58 | 32.86 |
| δ15 | 592.72 | 588.83 | 14.79 | 5.58 | δ15 | 596.58 | 588.25 | 13.93 | 4.99 |
| δ16 | 512.88 | 510.24 | 59.20 | 34.82 | δ16 | 512.62 | 531.36 | 55.71 | 35.10 |
| ν17 | 491.18 | 478.45 | 12.45 | 5.50 | ν17 | 490.96 | 489.28 | 11.74 | 16.03 |
| δ18 | 443.61 | 419.10 | 7.53 | 28.49 | δ18 | 444.51 | 421.84 | 6.13 | 39.82 |
| δ19 | 419.52 | 413.01 | 17.56 | 10.81 | δ19 | 419.44 | 415.59 | 15.12 | 47.98 |
| δ20 | 343.71 | 151.04 | 40.06 | 31.65 | δ20 | 336.05 | 336.05 | 24.07 | 29.92 |
| δ21 | 318.11 | 448.90 | 36.67 | 2.71 | δ21 | 315.43 | 315.43 | 53.89 | 67.00 |
| δ22 | 184.03 | 63.02 | 7.58 | 1.82 | δ22 | 159.06 | 159.06 | 4.57 | 5.68 |
| δ23 | 145.81 | 199.47 | 1.92 | 1.41 | δ23 | 143.05 | 143.05 | 3.09 | 3.85 |
| δ24 | 93.51 | 93.55 | 0.40 | 0.84 | δ24 | 92.01 | 92.01 | 0.43 | 0.53 |

| | **fp17h** | | | | | **fp17h / $N_2$** | | | |
|---|---|---|---|---|---|---|---|---|---|
| Mode | $E_{harm}$ | $E_{anharm}$ | $I_{harm}$ | $I_{anharm}$ | Mode | $E_{harm}$ | $E_{anharm}$ | $I_{harm}$ | $I_{anharm}$ |
| ν1 | 3594.30 | 3403.72 | 48.87 | 32.75 | ν1 | 3591.17 | 3402.89 | 51.63 | 37.91 |
| ν2 | 3475.93 | 3301.91 | 44.53 | 65.84 | ν2 | 3472.01 | 3291.75 | 26.30 | 829.07 |
| ν3 | 3472.95 | 3341.07 | 87.03 | 46.81 | ν3 | 3467.09 | 3338.81 | 97.42 | 45.39 |
| ν4 | 2706.40 | 2588.32 | 7.90 | 5.71 | ν4 | 2706.20 | 2589.31 | 6.96 | 3.97 |
| ν5 | 2236.68 | 2189.43 | 123.78 | 100.24 | ν5 | 2232.68 | 2190.68 | 120.29 | 99.66 |
| ν6 | 1686.98 | 1657.11 | 177.43 | 91.60 | ν6 | 1675.21 | 1645.26 | 180.80 | 91.02 |
| δ7 | 1484.28 | 1437.57 | 212.96 | 170.08 | δ7 | 1486.67 | 1446.34 | 202.71 | 159.48 |
| δ8 | 1257.13 | 1212.89 | 197.37 | 195.36 | δ8 | 1260.58 | 1218.75 | 188.25 | 182.38 |
| ν9 | 1156.74 | 1132.85 | 72.69 | 32.13 | ν9 | 1158.25 | 1133.17 | 72.83 | 35.33 |
| ν10 | 1016.60 | 992.21 | 42.45 | 27.51 | ν10 | 1018.73 | 986.86 | 40.18 | 53.49 |
| δ11 | 877.07 | 868.26 | 29.36 | 22.24 | δ11 | 880.32 | 861.75 | 27.67 | 18.62 |
| δ12 | 830.30 | 815.92 | 58.75 | 65.16 | δ12 | 829.72 | 813.36 | 56.18 | 55.44 |
| δ13 | 818.64 | 795.76 | 52.90 | 53.99 | δ13 | 812.94 | 799.02 | 50.29 | 48.92 |
| δ14 | 723.36 | 704.20 | 31.16 | 28.00 | δ14 | 723.71 | 701.92 | 28.87 | 26.22 |
| δ15 | 597.49 | 588.24 | 9.75 | 6.92 | δ15 | 598.42 | 590.89 | 9.29 | 6.80 |
| δ16 | 508.98 | 517.30 | 58.98 | 22.56 | δ16 | 510.15 | 503.46 | 55.65 | 27.30 |
| ν17 | 499.29 | 489.06 | 7.27 | 4.39 | ν17 | 500.04 | 489.67 | 7.12 | 3.34 |
| δ18 | 438.94 | 420.34 | 8.69 | 42.67 | δ18 | 441.26 | 412.19 | 7.09 | 28.66 |
| δ19 | 428.72 | 424.12 | 18.52 | 14.53 | δ19 | 429.28 | 422.51 | 18.30 | 15.75 |
| δ20 | 345.57 | 379.73 | 23.41 | 32.63 | δ20 | 348.04 | 577.80 | 28.27 | 217.48 |
| δ21 | 306.23 | 305.27 | 5.21 | 4.76 | δ21 | 307.88 | 290.52 | 5.07 | 4.52 |

| | | | | | | | | | |
|---|---|---|---|---|---|---|---|---|---|
| δ22 | 147.32 | 277.77 | 67.99 | 9.08 | δ22 | 161.09 | 472.39 | 64.67 | 105.61 |
| δ23 | 146.67 | 146.40 | 0.22 | 0.21 | δ23 | 147.39 | 132.80 | 0.23 | 0.12 |
| δ24 | 76.84 | 104.66 | 9.24 | 5.71 | δ24 | 81.82 | 58.27 | 5.53 | 0.78 |

| | | **fp18a** | | | | | **fp18a / $N_2$** | | |
|---|---|---|---|---|---|---|---|---|---|
| Mode | $E_{harm}$ | $E_{anharm}$ | $I_{harm}$ | $I_{anharm}$ | Mode | $E_{harm}$ | $E_{anharm}$ | $I_{harm}$ | $I_{anharm}$ |
| ν1 | 3484.59 | 3314.75 | 13.10 | 9.50 | ν1 | 3483.06 | 3305.64 | 13.15 | 10.78 |
| ν2 | 3246.95 | 3104.13 | 3.22 | 1.52 | ν2 | 3246.29 | 3105.38 | 3.42 | 1.68 |
| ν3 | 3158.75 | 3032.08 | 17.11 | 13.56 | ν3 | 3157.23 | 3034.53 | 16.93 | 13.59 |
| ν4 | 2686.51 | 2570.42 | 2.85 | 1.92 | ν4 | 2686.80 | 2560.43 | 2.59 | 1.79 |
| ν5 | 2126.99 | 2075.47 | 687.98 | 263.89 | ν5 | 2120.45 | 2071.68 | 680.92 | 322.87 |
| ν6 | 1672.74 | 1632.01 | 289.15 | 216.55 | ν6 | 1664.51 | 1623.23 | 290.70 | 206.92 |
| δ7 | 1447.31 | 1390.47 | 5.29 | 9.16 | δ7 | 1443.90 | 1398.63 | 5.13 | 8.07 |
| ν8 | 1273.89 | 1237.01 | 229.77 | 156.11 | ν8 | 1273.16 | 1239.45 | 224.82 | 128.33 |
| δ9 | 1219.48 | 1185.57 | 66.50 | 87.80 | δ9 | 1218.89 | 1183.95 | 59.46 | 93.57 |
| δ10 | 990.90 | 961.60 | 1.79 | 3.34 | δ10 | 988.78 | 970.50 | 1.69 | 2.41 |
| δ11 | 964.69 | 947.78 | 51.12 | 20.72 | δ11 | 965.14 | 951.10 | 49.14 | 24.59 |
| ν12 | 873.42 | 849.24 | 89.97 | 18.84 | ν12 | 871.34 | 849.94 | 89.84 | 84.58 |
| δ13 | 844.32 | 825.09 | 82.88 | 62.75 | δ13 | 839.68 | 831.09 | 80.46 | 73.02 |
| δ14 | 735.63 | 716.99 | 79.17 | 65.82 | δ14 | 737.91 | 724.74 | 73.96 | 68.76 |
| δ15 | 695.25 | 673.24 | 18.40 | 7.48 | δ15 | 698.87 | 680.29 | 21.83 | 8.51 |
| δ16 | 625.94 | 614.39 | 10.62 | 10.57 | δ16 | 629.11 | 620.22 | 8.34 | 10.45 |
| ν17 | 580.75 | 565.72 | 7.46 | 5.20 | ν17 | 575.07 | 561.53 | 5.95 | 6.16 |
| δ18 | 511.79 | 500.54 | 0.04 | 0.13 | δ18 | 514.33 | 507.95 | 0.02 | 0.02 |
| δ19 | 415.23 | 410.75 | 13.11 | 11.04 | δ19 | 416.01 | 402.59 | 14.13 | 11.76 |
| δ20 | 367.16 | 373.56 | 2.33 | 3.84 | δ20 | 367.99 | 386.35 | 2.11 | 5.36 |
| δ21 | 326.86 | 318.28 | 20.04 | 13.21 | δ21 | 324.85 | 305.84 | 18.83 | 14.78 |
| δ22 | 299.95 | 291.56 | 0.44 | 2.32 | δ22 | 302.21 | 300.43 | 0.27 | 2.95 |
| δ23 | 137.11 | 136.07 | 1.72 | 1.61 | δ23 | 137.60 | 136.91 | 1.56 | 1.86 |
| δ24 | 17.77 | 41.55 | 0.93 | 0.10 | δ24 | 21.37 | 75.96 | 0.93 | 0.71 |
| ν12 + ν8 | - | 2083.22 | - | 136.68 | ν12 + ν8 | - | - | - | <100 |
| δ15 + δ7 | - | 2092.38 | - | 138.32 | δ15 + δ7 | - | - | - | <100 |
| δ13 + ν8 | - | - | - | <100 | δ13 + ν8 | - | 2062.47 | - | 168.64 |

| | | **fp18b** | | | | | **fp18b / $N_2$** | | |
|---|---|---|---|---|---|---|---|---|---|
| Mode | $E_{harm}$ | $E_{anharm}$ | $I_{harm}$ | $I_{anharm}$ | Mode | $E_{harm}$ | $E_{anharm}$ | $I_{harm}$ | $I_{anharm}$ |
| ν1 | 3498.40 | 3323.06 | 13.45 | 10.76 | ν1 | 3496.62 | 3320.49 | 14.56 | 11.65 |
| ν2 | 3245.49 | 3103.35 | 2.87 | 1.29 | ν2 | 3244.91 | 3102.10 | 3.13 | 1.56 |
| ν3 | 3157.79 | 3032.86 | 15.49 | 11.82 | ν3 | 3156.29 | 3030.77 | 15.51 | 12.09 |
| ν4 | 2690.62 | 2578.90 | 0.26 | 0.09 | ν4 | 2692.03 | 2580.89 | 0.39 | 0.11 |
| ν5 | 2126.96 | 2087.40 | 673.82 | 388.83 | ν5 | 2120.00 | 2085.14 | 660.72 | 542.69 |
| ν6 | 1670.19 | 1624.57 | 258.72 | 201.04 | ν6 | 1661.75 | 1619.25 | 266.15 | 219.05 |
| δ7 | 1448.31 | 1386.08 | 4.12 | 2.63 | δ7 | 1444.64 | 1395.15 | 4.33 | 3.68 |
| ν8 | 1276.87 | 1240.92 | 228.68 | 63.08 | ν8 | 1277.15 | 1247.74 | 222.40 | 95.47 |
| ν9 | 1211.82 | 1178.20 | 60.35 | 65.91 | ν9 | 1214.48 | 1177.47 | 50.48 | 81.51 |
| δ10 | 996.17 | 980.35 | 57.50 | 54.17 | δ10 | 995.57 | 998.87 | 56.12 | 37.03 |
| δ11 | 991.38 | 967.83 | 3.19 | 3.47 | δ11 | 989.16 | 967.43 | 2.30 | 1.54 |

| | | | | | | | | | |
|---|---|---|---|---|---|---|---|---|---|
| ν12 | 872.51 | 851.01 | 87.37 | 78.79 | ν12 | 872.08 | 866.13 | 84.57 | 85.91 |
| δ13 | 842.50 | 823.80 | 85.58 | 74.95 | δ13 | 841.30 | 818.10 | 83.95 | 86.46 |
| δ14 | 739.14 | 719.77 | 67.89 | 69.19 | δ14 | 740.24 | 718.55 | 66.42 | 51.82 |
| δ15 | 721.18 | 695.55 | 50.56 | 17.51 | δ15 | 720.40 | 696.43 | 46.57 | 39.85 |
| δ16 | 646.55 | 627.96 | 3.85 | 1.54 | δ16 | 646.04 | 639.65 | 3.61 | 2.96 |
| δ17 | 554.51 | 539.34 | 3.28 | 1.86 | δ17 | 555.76 | 541.23 | 3.40 | 2.47 |
| δ18 | 516.39 | 508.17 | 1.68 | 1.33 | δ18 | 516.34 | 511.42 | 1.43 | 1.48 |
| δ19 | 410.63 | 405.49 | 11.95 | 9.41 | δ19 | 410.78 | 402.92 | 11.72 | 8.39 |
| δ20 | 370.37 | 367.03 | 1.37 | 1.52 | δ20 | 370.19 | 379.04 | 1.32 | 1.49 |
| δ21 | 312.47 | 321.91 | 1.15 | 3.44 | δ21 | 313.18 | 304.80 | 1.13 | 1.86 |
| δ22 | 228.82 | 288.18 | 7.21 | 3.19 | δ22 | 223.03 | 713.22 | 7.16 | 3.67 |
| δ23 | 136.97 | 135.26 | 0.76 | 0.88 | δ23 | 136.54 | 152.67 | 0.86 | 8.07 |
| δ24 | 37.87 | 37.43 | 0.35 | 0.23 | δ24 | 39.36 | 272.31 | 0.38 | 69.95 |
| δ13 + ν8 | - | 2061.08 | - | 179.25 | δ13 + ν8 | - | - | - | <100 |

| | | **fp18c** | | | | | **fp18c / $N_2$** | | |
|---|---|---|---|---|---|---|---|---|---|
| Mode | $E_{harm}$ | $E_{anharm}$ | $I_{harm}$ | $I_{anharm}$ | Mode | $E_{harm}$ | $E_{anharm}$ | $I_{harm}$ | $I_{anharm}$ |
| ν1 | 3520.80 | 3341.61 | 12.72 | 10.72 | ν1 | 3520.14 | 3339.27 | 14.00 | 11.74 |
| ν2 | 3247.01 | 3104.43 | 3.27 | 1.63 | ν2 | 3246.11 | 3104.73 | 3.45 | 1.87 |
| ν3 | 3158.62 | 3034.10 | 17.11 | 13.56 | ν3 | 3157.00 | 3032.54 | 16.89 | 13.70 |
| ν4 | 2682.42 | 2561.40 | 0.74 | 0.91 | ν4 | 2683.85 | 2572.69 | 0.47 | 0.80 |
| ν5 | 2128.80 | 2088.71 | 747.48 | 544.82 | ν5 | 2120.07 | 2080.56 | 721.09 | 548.53 |
| ν6 | 1662.07 | 1619.94 | 204.17 | 158.53 | ν6 | 1655.63 | 1617.81 | 208.95 | 173.40 |
| δ7 | 1448.60 | 1395.03 | 9.44 | 5.06 | δ7 | 1445.06 | 1397.59 | 9.06 | 5.39 |
| ν8 | 1292.28 | 1252.35 | 250.93 | 26.41 | ν8 | 1289.92 | 1268.00 | 241.94 | 148.83 |
| δ9 | 1197.18 | 1160.95 | 113.33 | 98.44 | δ9 | 1199.20 | 1203.76 | 104.68 | 59.71 |
| δ10 | 989.63 | 965.33 | 0.53 | 1.16 | δ10 | 987.79 | 965.83 | 0.68 | 1.06 |
| δ11 | 970.92 | 949.07 | 20.01 | 6.41 | δ11 | 970.89 | 965.36 | 18.43 | 30.23 |
| ν12 | 886.10 | 855.89 | 157.12 | 117.48 | ν12 | 884.20 | 920.62 | 152.24 | 147.17 |
| δ13 | 832.14 | 821.15 | 49.67 | 7.51 | δ13 | 833.17 | 801.33 | 47.86 | 22.20 |
| δ14 | 759.69 | 728.18 | 51.48 | 32.69 | δ14 | 759.18 | 755.59 | 48.60 | 51.93 |
| δ15 | 738.76 | 718.37 | 78.30 | 66.15 | δ15 | 740.40 | 723.28 | 75.08 | 61.32 |
| δ16 | 670.06 | 654.15 | 6.62 | 3.06 | δ16 | 670.22 | 656.78 | 6.61 | 5.67 |
| ν17 | 527.32 | 517.35 | 6.59 | 5.02 | ν17 | 527.65 | 533.05 | 6.72 | 6.45 |
| δ18 | 509.08 | 500.78 | 1.38 | 3.07 | δ18 | 511.02 | 497.09 | 0.89 | 0.92 |
| δ19 | 435.77 | 427.59 | 3.82 | 3.25 | δ19 | 436.38 | 454.57 | 4.00 | 7.23 |
| δ20 | 371.33 | 369.14 | 1.60 | 1.46 | δ20 | 372.01 | 368.43 | 1.54 | 1.40 |
| δ21 | 303.58 | 311.02 | 1.86 | 0.83 | δ21 | 304.70 | 317.98 | 1.96 | 1.03 |
| δ22 | 275.46 | 179.03 | 6.73 | 6.07 | δ22 | 275.75 | 446.91 | 6.47 | 18.14 |
| δ23 | 140.60 | 139.23 | 5.29 | 6.06 | δ23 | 141.32 | 136.83 | 5.06 | 4.51 |
| δ24 | 46.45 | 50.24 | 4.55 | 4.23 | δ24 | 44.40 | 28.63 | 4.35 | 3.50 |
| δ21 + δ10 | - | 1262.66 | - | 211.75 | δ21 + δ10 | - | - | - | <100 |
| δ16 + δ7 | - | 2072.51 | - | 104.83 | δ16 + δ7 | - | - | - | <100 |

| | | **fp18d** | | | | | **fp18d / $N_2$** | | |
|---|---|---|---|---|---|---|---|---|---|
| Mode | $E_{harm}$ | $E_{anharm}$ | $I_{harm}$ | $I_{anharm}$ | Mode | $E_{harm}$ | $E_{anharm}$ | $I_{harm}$ | $I_{anharm}$ |
| ν1 | 3503.48 | 3337.43 | 14.32 | 11.41 | ν1 | 3502.17 | 3330.15 | 15.03 | 14.83 |

| | | | | | | | | | |
|---|---|---|---|---|---|---|---|---|---|
| ν2 | 3246.22 | 3103.17 | 3.15 | 1.59 | ν2 | 3245.52 | 3101.04 | 3.30 | 2.70 |
| ν3 | 3158.02 | 3032.71 | 15.88 | 12.30 | ν3 | 3156.55 | 3026.18 | 15.71 | 14.83 |
| ν4 | 2685.90 | 2554.27 | 1.08 | 0.69 | ν4 | 2686.34 | 2544.62 | 1.22 | 1.21 |
| ν5 | 2126.07 | 2085.38 | 694.40 | 521.92 | ν5 | 2118.76 | 2080.90 | 673.37 | 615.58 |
| ν6 | 1662.69 | 1615.53 | 212.95 | 44.87 | ν6 | 1655.89 | 1606.91 | 221.63 | 213.48 |
| δ7 | 1448.41 | 1395.98 | 7.72 | 5.03 | δ7 | 1445.34 | 1396.65 | 7.60 | 10.32 |
| ν8 | 1296.24 | 1261.89 | 234.56 | 150.95 | ν8 | 1294.86 | 1258.21 | 223.34 | 147.14 |
| δ9 | 1200.00 | 1168.91 | 94.92 | 101.04 | δ9 | 1201.56 | 1147.81 | 88.19 | 107.69 |
| δ10 | 990.19 | 968.33 | 0.64 | 0.78 | δ10 | 988.60 | 964.43 | 0.78 | 2.96 |
| δ11 | 965.71 | 941.18 | 52.36 | 39.98 | δ11 | 965.35 | 917.83 | 47.48 | 83.52 |
| ν12 | 858.25 | 858.33 | 68.78 | 78.87 | ν12 | 858.27 | 845.45 | 74.59 | 92.03 |
| δ13 | 844.06 | 794.86 | 67.25 | 20.80 | δ13 | 843.29 | 790.93 | 58.78 | 15.45 |
| δ14 | 762.11 | 747.72 | 50.96 | 27.70 | δ14 | 760.63 | 747.76 | 48.30 | 68.12 |
| δ15 | 741.96 | 720.40 | 78.39 | 60.41 | δ15 | 743.07 | 704.57 | 75.27 | 28.18 |
| δ16 | 672.86 | 658.88 | 4.91 | 4.02 | δ16 | 672.54 | 657.79 | 4.86 | 5.12 |
| ν17 | 523.61 | 512.44 | 7.74 | 6.67 | ν17 | 526.33 | 515.48 | 6.78 | 10.72 |
| δ18 | 513.56 | 507.22 | 1.27 | 0.78 | δ18 | 513.93 | 500.22 | 1.73 | 1.53 |
| δ19 | 416.30 | 409.41 | 4.42 | 3.92 | δ19 | 417.16 | 405.90 | 4.57 | 2.73 |
| δ20 | 373.06 | 368.91 | 1.15 | 0.78 | δ20 | 373.55 | 373.55 | 1.11 | 1.38 |
| δ21 | 313.08 | 312.26 | 3.69 | 5.24 | δ21 | 313.79 | 313.79 | 3.66 | 4.55 |
| δ22 | 220.42 | 29.45 | 23.77 | 3.02 | δ22 | 217.67 | 217.67 | 23.12 | 28.74 |
| δ23 | 140.60 | 179.69 | 2.63 | 12.94 | δ23 | 140.36 | 140.36 | 2.30 | 2.86 |
| δ24 | 50.75 | 48.73 | 3.15 | 3.14 | δ24 | 48.04 | 48.04 | 3.14 | 3.91 |
| δ16 + δ10 | - | 1623.06 | - | 148.17 | δ16 + δ10 | - | - | - | <100 |

| | | **fp23** | | | | | **fp23 / $N_2$** | | |
|---|---|---|---|---|---|---|---|---|---|
| Mode | $E_{harm}$ | $E_{anharm}$ | $I_{harm}$ | $I_{anharm}$ | Mode | $E_{harm}$ | $E_{anharm}$ | $I_{harm}$ | $I_{anharm}$ |
| ν1 | 3510.65 | 3380.42 | 0.00 | 0.00 | ν1 | 3505.22 | 3374.19 | 0.00 | 0.00 |
| ν2 | 3408.59 | 3288.47 | 91.12 | 85.42 | ν2 | 3402.71 | 3281.71 | 86.75 | 82.59 |
| ν3 | 2063.67 | 2033.48 | 0.00 | 0.00 | ν3 | 2060.27 | 2030.45 | 0.00 | 0.00 |
| δ4 | 761.90 | 751.58 | 97.83 | 98.89 | δ4 | 763.64 | 749.55 | 89.16 | 90.54 |
| δ5 | 761.90 | 751.58 | 97.83 | 98.89 | δ5 | 763.64 | 749.55 | 89.16 | 90.54 |
| δ6 | 654.73 | 661.43 | 0.00 | 0.00 | δ6 | 658.79 | 660.97 | 0.00 | 0.00 |
| δ7 | 654.73 | 661.43 | 0.00 | 0.00 | δ7 | 658.79 | 660.97 | 0.00 | 0.00 |

| | | **fp26** | | | | | **fp26 / $N_2$** | | |
|---|---|---|---|---|---|---|---|---|---|
| Mode | $E_{harm}$ | $E_{anharm}$ | $I_{harm}$ | $I_{anharm}$ | Mode | $E_{harm}$ | $E_{anharm}$ | $I_{harm}$ | $I_{anharm}$ |
| ν1 | 3557.26 | 3379.69 | 65.77 | 50.99 | ν1 | 3550.80 | 3374.33 | 68.55 | 53.78 |
| ν2 | 2678.44 | 2551.88 | 0.13 | 0.41 | ν2 | 2679.41 | 2556.10 | 0.10 | 0.24 |
| ν3 | 2211.25 | 2161.46 | 898.05 | 786.46 | ν3 | 2200.15 | 2152.18 | 894.12 | 796.96 |
| ν4 | 1354.27 | 1367.26 | 0.04 | 0.31 | ν4 | 1353.53 | 1365.81 | 0.03 | 0.24 |
| δ5 | 1026.82 | 1003.80 | 16.05 | 14.67 | δ5 | 1026.13 | 1000.82 | 16.45 | 13.76 |
| δ6 | 943.95 | 884.13 | 267.26 | 209.56 | δ6 | 945.47 | 889.59 | 252.84 | 169.21 |
| ν7 | 678.05 | 668.13 | 19.58 | 16.14 | ν7 | 677.57 | 666.60 | 20.56 | 17.93 |
| δ8 | 602.57 | 606.06 | 60.39 | 14.70 | δ8 | 601.77 | 603.73 | 58.89 | 18.17 |
| δ9 | 589.57 | 558.30 | 21.17 | 56.30 | δ9 | 588.87 | 564.00 | 17.05 | 47.55 |
| δ10 | 464.38 | 459.49 | 44.25 | 46.38 | δ10 | 464.35 | 454.14 | 41.39 | 46.49 |

| | | | | | | | | | |
|---|---|---|---|---|---|---|---|---|---|
| δ11 | 200.75 | 129.74 | 17.88 | 14.46 | δ11 | 199.67 | 180.49 | 16.83 | 19.16 |
| δ12 | 175.48 | 175.09 | 5.31 | 6.65 | δ12 | 175.26 | 171.14 | 5.59 | 8.72 |
| | | **fp27** | | | | | **fp27 / $N_2$** | | |
| Mode | $E_{harm}$ | $E_{anharm}$ | $I_{harm}$ | $I_{anharm}$ | Mode | $E_{harm}$ | $E_{anharm}$ | $I_{harm}$ | $I_{anharm}$ |
| ν1 | 3637.81 | 3473.48 | 65.00 | 59.20 | ν1 | 3630.32 | 3470.35 | 65.79 | 60.86 |
| ν2 | 3547.79 | 3397.58 | 39.88 | 34.20 | ν2 | 3539.54 | 3392.36 | 45.60 | 40.33 |
| ν3 | 2351.75 | 2317.15 | 116.63 | 113.36 | ν3 | 2343.69 | 2309.74 | 132.04 | 128.19 |
| δ4 | 1628.74 | 1590.07 | 39.50 | 31.51 | δ4 | 1623.78 | 1581.53 | 38.94 | 25.89 |
| δ5 | 1190.57 | 1154.68 | 0.03 | 0.19 | δ5 | 1191.14 | 1151.93 | 0.09 | 0.31 |
| ν6 | 1092.90 | 1088.26 | 9.80 | 14.44 | ν6 | 1099.27 | 1095.84 | 9.33 | 11.37 |
| δ7 | 566.99 | 261.86 | 147.84 | 167.26 | δ7 | 562.36 | 293.04 | 120.22 | 173.71 |
| δ8 | 489.71 | 599.26 | 75.44 | 6.76 | δ8 | 494.07 | 603.42 | 87.87 | 3.48 |
| δ9 | 409.99 | 416.72 | 0.24 | 0.10 | δ9 | 418.26 | 430.32 | 0.27 | 0.20 |
| | | **fp28** | | | | | **fp28 / $N_2$** | | |
| Mode | $E_{harm}$ | $E_{anharm}$ | $I_{harm}$ | $I_{anharm}$ | Mode | $E_{harm}$ | $E_{anharm}$ | $I_{harm}$ | $I_{anharm}$ |
| ν1 | 3330.70 | 3194.36 | 6.60 | 3.59 | ν1 | 3331.19 | 3193.14 | 7.74 | 4.76 |
| ν2 | 3275.59 | 3148.82 | 24.58 | 19.47 | ν2 | 3275.59 | 3146.88 | 26.66 | 21.28 |
| ν3 | 1747.51 | 1711.11 | 18.37 | 15.18 | ν3 | 1748.22 | 1710.28 | 17.76 | 14.11 |
| δ4 | 945.35 | 921.37 | 44.50 | 45.48 | δ4 | 943.94 | 883.64 | 42.00 | 40.85 |
| δ5 | 911.66 | 950.83 | 0.47 | 35.98 | δ5 | 910.28 | 826.73 | 0.64 | 15.60 |
| δ6 | 772.81 | 759.72 | 0.00 | 0.00 | δ6 | 773.86 | 768.55 | 0.00 | 0.00 |
| ν7 | 663.67 | 645.36 | 8.40 | 8.82 | ν7 | 657.97 | 639.46 | 9.10 | 9.77 |
| δ8 | 590.61 | 579.76 | 71.43 | 73.42 | δ8 | 597.19 | 596.03 | 66.59 | 68.15 |
| ν9 | 455.01 | 437.63 | 0.70 | 0.66 | ν9 | 457.10 | 438.30 | 0.50 | 0.53 |
| | | **fp29** | | | | | **fp29 / $N_2$** | | |
| Mode | $E_{harm}$ | $E_{anharm}$ | $I_{harm}$ | $I_{anharm}$ | Mode | $E_{harm}$ | $E_{anharm}$ | $I_{harm}$ | $I_{anharm}$ |
| ν1 | 3597.94 | 3435.05 | 24.79 | 37.67 | ν1 | 3589.57 | 3424.82 | 24.34 | 20.02 |
| ν2 | 3595.06 | 3432.91 | 145.40 | 138.60 | ν2 | 3585.79 | 3421.92 | 144.45 | 143.98 |
| ν3 | 2218.79 | 2203.63 | 701.04 | 393.35 | ν3 | 2205.83 | 2198.35 | 684.08 | 366.35 |
| ν4 | 1287.61 | 1275.25 | 0.00 | 0.04 | ν4 | 1287.38 | 1274.97 | 0.01 | 0.01 |
| δ5 | 916.11 | 867.08 | 25.68 | 15.45 | δ5 | 920.87 | 881.95 | 12.71 | 14.72 |
| δ6 | 914.69 | 876.97 | 427.31 | 420.68 | δ6 | 912.62 | 883.66 | 415.53 | 408.51 |
| δ7 | 717.06 | 674.06 | 90.75 | 93.29 | δ7 | 716.72 | 682.92 | 81.65 | 84.46 |
| δ8 | 547.85 | 554.35 | 73.87 | 80.82 | δ8 | 546.21 | 567.60 | 71.82 | 70.95 |
| δ9 | 544.49 | 549.95 | 0.36 | 0.53 | δ9 | 543.45 | 554.81 | 0.29 | 1.00 |
| δ6 + ν4 | - | 2126.08 | - | 174.57 | δ6 + ν4 | - | 2123.32 | - | 289.68 |
| | | **fp30** | | | | | **fp30 / $N_2$** | | |
| Mode | $E_{harm}$ | $E_{anharm}$ | $I_{harm}$ | $I_{anharm}$ | Mode | $E_{harm}$ | $E_{anharm}$ | $I_{harm}$ | $I_{anharm}$ |
| ν1 | 3468.36 | 3339.33 | 89.00 | 79.64 | ν1 | 3462.28 | 3333.40 | 85.65 | 78.03 |
| ν2 | 2641.51 | 2525.36 | 0.82 | 1.23 | ν2 | 2643.40 | 2529.24 | 0.82 | 0.93 |
| ν3 | 2147.60 | 2112.40 | 18.96 | 19.75 | ν3 | 2143.98 | 2109.30 | 20.25 | 21.05 |
| δ4 | 976.20 | 953.61 | 12.46 | 11.36 | δ4 | 975.31 | 950.50 | 12.03 | 10.86 |
| δ5 | 726.02 | 713.10 | 32.15 | 36.65 | δ5 | 727.28 | 713.96 | 29.65 | 32.91 |
| ν6 | 713.42 | 701.37 | 1.98 | 4.72 | ν6 | 712.14 | 700.98 | 1.56 | 3.36 |
| δ7 | 578.68 | 558.93 | 56.46 | 58.31 | δ7 | 581.06 | 553.74 | 51.82 | 53.58 |

| | | | | | | | | | |
|---|---|---|---|---|---|---|---|---|---|
| δ8 | 366.73 | 371.08 | 10.22 | 8.89 | δ8 | 367.15 | 373.61 | 9.55 | 8.90 |
| δ9 | 302.26 | 307.90 | 8.54 | 8.26 | δ9 | 302.86 | 309.73 | 8.03 | 7.81 |

| | | **fp33a** | | | | | **fp33a / $N_2$** | | |
|---|---|---|---|---|---|---|---|---|---|
| Mode | $E_{harm}$ | $E_{anharm}$ | $I_{harm}$ | $I_{anharm}$ | Mode | $E_{harm}$ | $E_{anharm}$ | $I_{harm}$ | $I_{anharm}$ |
| ν1 | 3506.14 | 3335.66 | 134.05 | 120.26 | ν1 | 3500.77 | 3336.85 | 138.59 | 123.32 |
| ν2 | 3095.98 | 2960.62 | 12.47 | 18.02 | ν2 | 3103.35 | 2968.25 | 10.01 | 15.15 |
| ν3 | 3004.89 | 2856.49 | 7.91 | 13.54 | ν3 | 3012.49 | 2859.85 | 5.82 | 7.30 |
| ν4 | 2974.34 | 2821.31 | 23.42 | 5.82 | ν4 | 2982.19 | 2824.95 | 21.64 | 6.46 |
| ν5 | 2334.31 | 2289.50 | 237.25 | 124.66 | ν5 | 2327.61 | 2281.20 | 258.43 | 154.42 |
| δ6 | 1479.96 | 1449.78 | 100.30 | 62.42 | δ6 | 1476.37 | 1444.80 | 99.04 | 83.63 |
| δ7 | 1451.55 | 1405.29 | 48.69 | 4.09 | δ7 | 1448.12 | 1400.54 | 46.25 | 11.27 |
| δ8 | 1389.72 | 1353.31 | 44.27 | 40.41 | δ8 | 1389.09 | 1351.44 | 43.90 | 35.24 |
| δ9 | 1331.33 | 1298.72 | 38.85 | 46.66 | δ9 | 1332.36 | 1299.22 | 40.47 | 56.53 |
| δ10 | 1252.06 | 1217.33 | 3.86 | 4.73 | δ10 | 1253.01 | 1221.27 | 3.30 | 3.60 |
| ν11 | 1210.89 | 1186.19 | 11.68 | 5.79 | ν11 | 1209.91 | 1188.71 | 10.97 | 2.78 |
| ν12 | 1138.72 | 1122.95 | 72.83 | 62.17 | ν12 | 1138.89 | 1122.54 | 73.94 | 59.75 |
| ν13 | 1069.12 | 1053.17 | 14.18 | 5.81 | ν13 | 1066.72 | 1051.39 | 15.91 | 19.13 |
| δ14 | 996.44 | 970.27 | 9.50 | 9.23 | δ14 | 996.75 | 968.05 | 8.80 | 8.45 |
| ν15 | 867.06 | 854.04 | 4.25 | 4.09 | ν15 | 867.05 | 853.28 | 3.73 | 2.86 |
| δ16 | 734.97 | 720.94 | 6.80 | 3.41 | δ16 | 735.33 | 714.89 | 6.56 | 5.13 |
| δ17 | 614.08 | 598.26 | 4.48 | 5.07 | δ17 | 613.40 | 594.98 | 4.46 | 9.00 |
| δ18 | 554.21 | 537.99 | 2.17 | 3.79 | δ18 | 555.28 | 531.71 | 1.69 | 13.78 |
| δ19 | 524.62 | 510.22 | 10.18 | 7.86 | δ19 | 528.55 | 517.35 | 9.23 | 7.13 |
| δ20 | 360.21 | 123.39 | 64.06 | 12.00 | δ20 | 339.44 | 25.10 | 65.47 | 2.36 |
| δ21 | 236.28 | 232.48 | 2.67 | 10.09 | δ21 | 236.61 | 226.00 | 3.07 | 35.29 |
| δ22 | 204.15 | 170.22 | 3.39 | 14.43 | δ22 | 202.76 | 162.84 | 4.35 | 28.96 |
| δ23 | 134.37 | 135.52 | 14.44 | 16.44 | δ23 | 132.48 | 118.26 | 14.35 | 20.15 |
| δ24 | 54.68 | 23.75 | 4.04 | 0.48 | δ24 | 52.74 | 24.22 | 3.90 | 0.36 |

| | | **fp33b** | | | | | **fp33b / $N_2$** | | |
|---|---|---|---|---|---|---|---|---|---|
| Mode | $E_{harm}$ | $E_{anharm}$ | $I_{harm}$ | $I_{anharm}$ | Mode | $E_{harm}$ | $E_{anharm}$ | $I_{harm}$ | $I_{anharm}$ |
| ν1 | 3577.43 | 3412.83 | 42.71 | 22.39 | ν1 | 3580.73 | 3407.07 | 47.63 | 39.04 |
| ν2 | 3088.10 | 2983.10 | 15.69 | 14.77 | ν2 | 3093.03 | 2990.36 | 14.43 | 12.68 |
| ν3 | 3075.65 | 2897.17 | 2.12 | 13.90 | ν3 | 3081.10 | 2897.10 | 1.99 | 11.11 |
| ν4 | 3031.29 | 2918.07 | 9.15 | 6.23 | ν4 | 3035.08 | 2915.75 | 8.64 | 6.55 |
| ν5 | 2335.52 | 2308.62 | 197.16 | 124.65 | ν5 | 2329.58 | 2301.44 | 220.03 | 148.69 |
| δ6 | 1478.68 | 1442.19 | 46.85 | 29.23 | δ6 | 1481.33 | 1422.23 | 42.17 | 36.30 |
| δ7 | 1475.95 | 1428.63 | 8.07 | 18.04 | δ7 | 1469.85 | 1421.76 | 12.25 | 7.44 |
| δ8 | 1413.92 | 1376.27 | 5.19 | 4.27 | δ8 | 1413.01 | 1375.91 | 4.42 | 5.75 |
| δ9 | 1325.08 | 1290.47 | 34.23 | 28.41 | δ9 | 1324.08 | 1297.46 | 34.27 | 22.85 |
| δ10 | 1245.85 | 1224.13 | 4.82 | 3.03 | δ10 | 1249.88 | 1228.85 | 3.64 | 5.84 |
| ν11 | 1168.66 | 1147.48 | 74.71 | 74.16 | ν11 | 1173.82 | 1143.52 | 71.65 | 50.75 |
| ν12 | 1152.69 | 1131.21 | 26.13 | 6.74 | ν12 | 1150.02 | 1118.78 | 27.54 | 25.60 |
| δ13 | 1058.37 | 1040.45 | 43.50 | 45.68 | δ13 | 1056.86 | 1036.33 | 44.54 | 33.33 |
| ν14 | 1001.21 | 980.00 | 3.96 | 0.85 | ν14 | 997.90 | 969.97 | 3.59 | 1.80 |
| ν15 | 923.46 | 904.93 | 1.35 | 1.14 | ν15 | 925.19 | 896.95 | 1.25 | 1.51 |

| | | | | | | | | | |
|---|---|---|---|---|---|---|---|---|---|
| δ16 | 782.51 | 747.90 | 14.98 | 8.33 | δ16 | 776.57 | 741.79 | 14.35 | 7.47 |
| δ17 | 614.63 | 580.68 | 19.79 | 5.30 | δ17 | 612.43 | 583.36 | 14.60 | 5.46 |
| δ18 | 530.90 | 524.92 | 21.61 | 4.49 | δ18 | 530.79 | 526.63 | 16.57 | 8.89 |
| δ19 | 442.11 | 406.59 | 64.86 | 23.88 | δ19 | 432.38 | 421.70 | 68.36 | 8.08 |
| δ20 | 366.87 | 320.20 | 7.29 | 58.37 | δ20 | 364.69 | 327.39 | 11.81 | 34.23 |
| δ21 | 335.96 | 317.34 | 2.57 | 1.36 | δ21 | 332.59 | 316.18 | 2.30 | 11.53 |
| δ22 | 178.15 | 176.23 | 3.44 | 4.86 | δ22 | 181.18 | 183.15 | 3.19 | 4.33 |
| δ23 | 53.59 | 46.48 | 10.97 | 11.56 | δ23 | 54.69 | 50.36 | 10.77 | 9.11 |
| δ24 | 41.11 | 27.18 | 10.15 | 6.95 | δ24 | 36.06 | 75.59 | 9.26 | 5.06 |
| | | **fp36** | | | | | **fp36 / $N_2$** | | |
| Mode | $E_{harm}$ | $E_{anharm}$ | $I_{harm}$ | $I_{anharm}$ | Mode | $E_{harm}$ | $E_{anharm}$ | $I_{harm}$ | $I_{anharm}$ |
| ν1 | 3677.79 | 3514.08 | 242.02 | 219.73 | ν1 | 3669.78 | 3506.88 | 249.78 | 229.49 |
| ν2 | 2038.38 | 2009.95 | 778.54 | 741.41 | ν2 | 2030.05 | 2002.54 | 786.84 | 749.73 |
| ν3 | 880.95 | 855.26 | 2.78 | 4.77 | ν3 | 877.85 | 852.14 | 3.21 | 4.81 |
| δ4 | 649.92 | 602.23 | 333.77 | 250.19 | δ4 | 632.40 | 603.10 | 319.47 | 253.50 |
| δ5 | 462.44 | 481.64 | 82.08 | 137.38 | δ5 | 459.57 | 492.18 | 91.38 | 120.95 |
| δ6 | 494.06 | 525.32 | 3.20 | 3.51 | δ6 | 492.86 | 524.62 | 3.04 | 3.32 |
| | | **fp39** | | | | | **fp39 / $N_2$** | | |
| Mode | $E_{harm}$ | $E_{anharm}$ | $I_{harm}$ | $I_{anharm}$ | Mode | $E_{harm}$ | $E_{anharm}$ | $I_{harm}$ | $I_{anharm}$ |
| ν1 | 3478.65 | 3295.76 | 22.17 | 15.42 | ν1 | 3474.06 | 3293.89 | 25.30 | 19.14 |
| ν2 | 3248.03 | 3105.84 | 1.19 | 0.50 | ν2 | 3244.91 | 3103.34 | 1.34 | 0.62 |
| ν3 | 3160.57 | 3035.94 | 9.41 | 5.84 | ν3 | 3156.56 | 3032.09 | 9.46 | 5.86 |
| ν4 | 2113.85 | 2072.05 | 436.14 | 365.29 | ν4 | 2105.09 | 2065.89 | 433.99 | 352.36 |
| δ5 | 1437.90 | 1440.97 | 4.84 | 0.04 | δ5 | 1434.49 | 1437.20 | 4.39 | 0.06 |
| ν6 | 1164.11 | 1147.37 | 18.52 | 15.87 | ν6 | 1163.33 | 1146.21 | 19.19 | 15.42 |
| δ7 | 1023.74 | 983.62 | 208.91 | 209.68 | δ7 | 1025.93 | 981.07 | 195.51 | 198.82 |
| δ8 | 999.52 | 982.57 | 0.01 | 0.01 | δ8 | 998.48 | 983.59 | 0.03 | 0.01 |
| δ9 | 896.27 | 867.15 | 52.60 | 45.13 | δ9 | 894.67 | 868.00 | 47.94 | 44.47 |
| δ10 | 718.32 | 698.49 | 87.38 | 86.53 | δ10 | 718.12 | 696.63 | 81.53 | 80.68 |
| δ11 | 483.55 | 486.31 | 21.99 | 22.85 | δ11 | 483.70 | 484.19 | 20.79 | 22.47 |
| δ12 | 420.82 | 423.55 | 0.43 | 0.49 | δ12 | 421.54 | 425.89 | 0.39 | 0.45 |
| | | **fp49** | | | | | **fp49 / $N_2$** | | |
| Mode | $E_{harm}$ | $E_{anharm}$ | $I_{harm}$ | $I_{anharm}$ | Mode | $E_{harm}$ | $E_{anharm}$ | $I_{harm}$ | $I_{anharm}$ |
| ν1 | 3478.01 | 3330.23 | 362.74 | 319.76 | ν1 | 3463.72 | 3320.03 | 360.80 | 330.20 |
| ν2 | 2136.25 | 2090.20 | 340.45 | 331.18 | ν2 | 2137.10 | 2092.84 | 322.01 | 313.25 |
| ν3 | 764.34 | 786.66 | 54.06 | 1.27 | ν3 | 750.71 | 710.63 | 55.13 | 95.36 |
| δ4 | 432.87 | 431.56 | 6.61 | 4.99 | δ4 | 432.74 | 438.51 | 0.00 | 12.96 |
| δ5 | 432.87 | 431.56 | 6.61 | 4.99 | δ5 | 432.74 | 438.51 | 0.00 | 12.96 |
| δ6 | 354.42 | 375.44 | 57.44 | 61.67 | δ6 | 402.20 | 486.09 | 54.96 | 60.00 |
| δ7 | 354.42 | 375.44 | 57.44 | 61.67 | δ7 | 402.20 | 486.09 | 54.96 | 60.00 |

**Table S2.** Anharmonic and harmonic wavenumbers (in $cm^{-1}$) calculated for ACA complexes at the B3LYP-D3/6-311++G(3df,3pd) level of theory. The IR calculated intensities expressed in km $mol^{-1}$. Assignment: ν – stretching modes, δ – bending modes of all kinds.

| | | fp 27-28a | | | | | fp 27-28b | | |
|---|---|---|---|---|---|---|---|---|---|
| Mode | $E_{harm}$ | $E_{anharm}$ | $I_{harm}$ | $I_{anharm}$ | Mode | $E_{harm}$ | $E_{anharm}$ | $I_{harm}$ | $I_{anharm}$ |
| ν1 | 3604.68 | 3450.91 | 90.34 | 59.25 | ν1 | 3600.47 | 3427.90 | 96.47 | 38.22 |
| ν2 | 3347.93 | 3172.23 | 457.94 | 171.74 | ν2 | 3335.85 | 3252.70 | 174.64 | 9.06 |
| ν3 | 3332.70 | 3204.56 | 14.32 | 111.81 | ν3 | 3331.57 | 3070.06 | 319.78 | 376.71 |
| ν4 | 3276.18 | 3142.71 | 42.99 | 39.26 | ν4 | 3278.02 | 3149.88 | 41.36 | 65.40 |
| ν5 | 2335.92 | 2250.56 | 122.66 | 110.36 | ν5 | 2336.42 | 2304.23 | 124.43 | 111.88 |
| ν6 | 1752.60 | 1713.94 | 12.26 | 10.20 | ν6 | 1752.63 | 1714.08 | 11.34 | 9.61 |
| δ7 | 1617.05 | 1568.77 | 30.93 | 35.54 | δ7 | 1622.64 | 1573.69 | 39.39 | 41.00 |
| δ8 | 1219.42 | 1173.98 | 8.78 | 13.42 | δ8 | 1222.15 | 1180.30 | 7.14 | 11.12 |
| ν9 | 1104.61 | 1105.81 | 9.13 | 13.23 | ν9 | 1102.38 | 1103.62 | 8.61 | 12.39 |
| δ10 | 952.49 | 927.39 | 38.17 | 33.91 | δ10 | 951.57 | 923.15 | 39.75 | 33.71 |
| δ11 | 918.83 | 894.82 | 2.52 | 0.93 | δ11 | 918.51 | 891.81 | 1.98 | 1.44 |
| δ12 | 775.20 | 767.63 | 2.52 | 3.17 | δ12 | 773.75 | 757.30 | 1.53 | 3.82 |
| ν13 | 653.44 | 643.33 | 14.40 | 4.21 | δ13 | 666.85 | 531.04 | 128.36 | 60.20 |
| δ14 | 648.28 | 638.08 | 133.82 | 67.97 | ν14 | 652.69 | 634.67 | 11.81 | 11.08 |
| δ15 | 617.00 | 607.12 | 91.81 | 55.24 | δ15 | 615.90 | 585.59 | 81.37 | 61.46 |
| δ16 | 529.56 | 523.11 | 5.91 | 10.50 | δ16 | 526.23 | 521.43 | 4.88 | 5.79 |
| ν17 | 471.22 | 455.85 | 0.49 | 0.38 | ν17 | 472.90 | 456.27 | 0.55 | 0.37 |
| δ18 | 431.21 | 427.72 | 0.85 | 1.19 | δ18 | 436.01 | 433.08 | 0.98 | 1.68 |
| δ19 | 284.22 | 237.90 | 58.32 | 60.71 | δ19 | 281.53 | 219.31 | 59.25 | 57.07 |
| δ20 | 146.82 | 138.80 | 20.50 | 65.98 | δ20 | 151.53 | 139.77 | 29.24 | 31.04 |
| δ21 | 143.87 | 122.43 | 11.59 | 0.81 | δ21 | 139.29 | 86.01 | 7.38 | 11.91 |
| δ22 | 78.50 | 73.13 | 10.31 | 2.78 | δ22 | 78.17 | 68.01 | 12.51 | 4.73 |
| δ23 | 59.29 | 33.58 | 0.92 | 2.28 | δ23 | 54.23 | 13.48 | 2.13 | 1.51 |
| δ24 | 51.10 | 87.38 | 10.81 | 16.95 | δ24 | 49.74 | 29.63 | 13.08 | 11.58 |
| | | **fp 27-30a** | | | | | **fp 27-30b** | | |
| Mode | $E_{harm}$ | $E_{anharm}$ | $I_{harm}$ | $I_{anharm}$ | Mode | $E_{harm}$ | $E_{anharm}$ | $I_{harm}$ | $I_{anharm}$ |
| ν1 | 3615.70 | 3462.82 | 113.27 | 63.97 | ν1 | 3624.53 | 3456.42 | 122.26 | 65.09 |
| ν2 | 3495.88 | 3348.41 | 105.03 | 110.45 | ν2 | 3502.73 | 3343.39 | 87.97 | 96.40 |
| ν3 | 3461.58 | 3312.54 | 96.63 | 84.32 | ν3 | 3462.18 | 3330.65 | 93.74 | 81.55 |
| ν4 | 2597.99 | 2462.19 | 73.16 | 73.45 | ν4 | 2609.65 | 2465.58 | 59.49 | 62.76 |
| ν5 | 2340.95 | 2253.91 | 111.17 | 105.31 | ν5 | 2343.26 | 2309.33 | 114.42 | 109.89 |
| ν6 | 2132.36 | 2097.71 | 30.70 | 31.88 | ν6 | 2131.58 | 2091.98 | 33.97 | 34.84 |
| δ7 | 1617.15 | 1573.70 | 39.70 | 38.54 | δ7 | 1617.28 | 1570.13 | 42.71 | 42.59 |
| δ8 | 1206.34 | 1173.70 | 4.19 | 3.48 | δ8 | 1200.44 | 1167.73 | 4.00 | 3.06 |
| ν9 | 1103.04 | 1103.45 | 6.64 | 7.92 | ν9 | 1103.96 | 1100.20 | 6.49 | 9.26 |
| δ10 | 1002.59 | 969.74 | 4.66 | 3.79 | δ10 | 998.79 | 970.90 | 4.62 | 3.46 |
| δ11 | 715.34 | 674.02 | 30.42 | 33.12 | δ11 | 714.81 | 710.75 | 10.13 | 1.26 |
| ν12 | 713.01 | 710.92 | 13.05 | 0.30 | ν12 | 712.72 | 671.43 | 33.42 | 36.75 |
| δ13 | 609.39 | 599.95 | 149.73 | 106.22 | δ13 | 571.59 | 374.81 | 99.56 | 62.95 |
| δ14 | 563.36 | 533.79 | 52.70 | 61.62 | δ14 | 556.95 | 533.73 | 78.36 | 223.69 |
| δ15 | 514.10 | 511.45 | 27.54 | 10.32 | δ15 | 502.80 | 483.40 | 61.07 | 57.93 |

| Mode | $E_{harm}$ | $E_{anharm}$ | $I_{harm}$ | $I_{anharm}$ | Mode | $E_{harm}$ | $E_{anharm}$ | $I_{harm}$ | $I_{anharm}$ |
|---|---|---|---|---|---|---|---|---|---|
| δ16 | 421.79 | 421.37 | 0.09 | 5.80 | δ16 | 425.57 | 423.28 | 0.31 | 5.54 |
| δ17 | 386.91 | 380.74 | 12.59 | 8.37 | δ17 | 386.83 | 379.36 | 14.30 | 10.31 |
| δ18 | 323.03 | 317.31 | 12.74 | 10.50 | δ18 | 322.38 | 327.13 | 13.19 | 10.40 |
| δ19 | 264.55 | 202.78 | 22.65 | 23.58 | δ19 | 250.95 | 156.05 | 10.13 | 50.44 |
| δ20 | 219.84 | 184.25 | 39.38 | 33.09 | δ20 | 190.45 | 214.59 | 48.51 | 14.87 |
| δ21 | 119.02 | 130.58 | 8.68 | 13.29 | δ21 | 118.04 | 133.54 | 11.91 | 25.77 |
| δ22 | 103.48 | 94.65 | 10.79 | 1.38 | δ22 | 101.85 | 107.67 | 10.65 | 0.27 |
| δ23 | 61.74 | 65.62 | 10.21 | 16.40 | δ23 | 59.56 | 63.66 | 10.53 | 5.14 |
| δ24 | 41.70 | 31.18 | 11.21 | 8.65 | δ24 | 33.91 | 55.01 | 9.18 | 4.98 |
| | | **fp 27-30c** | | | | | **fp 28-29** | | |
| Mode | $E_{harm}$ | $E_{anharm}$ | $I_{harm}$ | $I_{anharm}$ | Mode | $E_{harm}$ | $E_{anharm}$ | $I_{harm}$ | $I_{anharm}$ |
| ν1 | 3640.58 | 3469.38 | 71.24 | 23.23 | ν1 | 3583.07 | 3410.26 | 69.84 | 62.26 |
| ν2 | 3548.08 | 3393.51 | 52.80 | 74.01 | ν2 | 3406.59 | 3260.17 | 473.26 | 235.17 |
| ν3 | 3387.60 | 3262.71 | 445.95 | 343.88 | ν3 | 3330.63 | 3193.32 | 8.97 | 138.37 |
| ν4 | 2637.89 | 2538.48 | 1.46 | 6.11 | ν4 | 3274.18 | 3147.07 | 38.46 | 31.56 |
| ν5 | 2358.15 | 2322.37 | 166.08 | 159.49 | ν5 | 2211.80 | 2130.12 | 625.50 | 367.65 |
| ν6 | 2138.71 | 2113.40 | 0.34 | 0.62 | ν6 | 1752.63 | 1715.13 | 12.65 | 10.72 |
| δ7 | 1627.20 | 1587.28 | 41.14 | 116.63 | ν7 | 1284.30 | 1275.44 | 1.72 | 1.58 |
| δ8 | 1187.96 | 1146.78 | 0.11 | 1.01 | δ8 | 974.08 | 958.41 | 226.23 | 261.53 |
| ν9 | 1103.61 | 1093.95 | 10.01 | 43.35 | δ9 | 955.66 | 911.11 | 156.82 | 34.64 |
| δ10 | 974.94 | 950.87 | 15.68 | 42.87 | δ10 | 950.47 | 939.47 | 24.90 | 169.26 |
| δ11 | 812.94 | 746.20 | 26.93 | 21.63 | δ11 | 919.32 | 891.15 | 3.23 | 2.37 |
| ν12 | 713.87 | 705.97 | 0.84 | 4.11 | δ12 | 792.24 | 733.46 | 55.49 | 59.46 |
| δ13 | 699.67 | 647.12 | 46.49 | 38.05 | δ13 | 776.61 | 774.67 | 2.38 | 3.94 |
| δ14 | 550.84 | 429.70 | 110.38 | 1943.46 | ν14 | 656.35 | 638.84 | 9.10 | 9.58 |
| δ15 | 483.75 | 377.03 | 127.97 | 2854.61 | δ15 | 615.80 | 605.93 | 93.97 | 81.90 |
| δ16 | 414.76 | 417.76 | 0.15 | 5.34 | δ16 | 565.45 | 562.08 | 19.76 | 32.38 |
| δ17 | 369.25 | 369.25 | 6.73 | 6.73 | δ17 | 550.75 | 540.30 | 22.32 | 13.47 |
| δ18 | 308.63 | 308.63 | 6.85 | 6.85 | ν18 | 469.02 | 450.86 | 0.33 | 0.19 |
| δ19 | 73.30 | 73.30 | 2.72 | 2.72 | δ19 | 290.57 | 260.63 | 131.09 | 114.97 |
| δ20 | 68.94 | 68.94 | 0.57 | 0.57 | δ20 | 128.72 | 95.55 | 1.22 | 0.20 |
| δ21 | 61.60 | 61.60 | 1.65 | 1.65 | δ21 | 113.79 | 139.37 | 21.06 | 0.88 |
| δ22 | 23.47 | 23.47 | 25.86 | 25.86 | δ22 | 70.21 | 75.67 | 4.28 | 14.58 |
| δ23 | 14.45 | 14.45 | 18.81 | 18.81 | δ23 | 64.48 | 46.20 | 4.46 | 2.54 |
| δ24 | 11.63 | 11.63 | 13.78 | 13.78 | δ24 | 42.11 | 31.08 | 1.62 | 1.87 |
| | | **fp 29-30a** | | | | | **fp 29-30b** | | |
| Mode | $E_{harm}$ | $E_{anharm}$ | $I_{harm}$ | $I_{anharm}$ | Mode | $E_{harm}$ | $E_{anharm}$ | $I_{harm}$ | $I_{anharm}$ |
| ν1 | 3589.41 | 3418.75 | 61.62 | 136.61 | ν1 | 3584.50 | 3416.02 | 62.34 | 113.82 |
| ν2 | 3567.56 | 3414.80 | 184.12 | 100.94 | ν2 | 3552.80 | 3378.30 | 212.26 | 142.75 |
| ν3 | 3465.51 | 3316.92 | 82.79 | 80.73 | ν3 | 3463.87 | 3334.87 | 94.43 | 84.36 |
| ν4 | 2639.63 | 2514.36 | 0.63 | 0.39 | ν4 | 2633.70 | 2510.86 | 16.78 | 11.69 |
| ν5 | 2215.89 | 2134.02 | 599.35 | 340.04 | ν5 | 2216.55 | 2209.33 | 570.17 | 286.91 |
| ν6 | 2151.11 | 2113.18 | 8.33 | 10.51 | ν6 | 2141.04 | 2110.41 | 9.78 | 10.82 |
| ν7 | 1286.40 | 1275.57 | 0.35 | 0.08 | ν7 | 1286.58 | 1276.29 | 0.36 | 0.31 |
| δ8 | 975.98 | 953.34 | 18.28 | 11.07 | δ8 | 995.29 | 969.48 | 14.31 | 15.61 |

| | | | | | | | | | |
|---|---|---|---|---|---|---|---|---|---|
| δ9 | 936.02 | 910.69 | 204.48 | 296.64 | δ9 | 944.17 | 897.70 | 231.14 | 338.70 |
| δ10 | 925.82 | 877.37 | 159.45 | 49.80 | δ10 | 936.79 | 887.74 | 135.55 | 115.51 |
| δ11 | 743.50 | 971.23 | 75.29 | 24.82 | δ11 | 742.81 | 771.08 | 78.93 | 90.06 |
| δ12 | 734.74 | 412.35 | 24.50 | 93.21 | δ12 | 722.03 | 587.49 | 16.80 | 32.10 |
| ν13 | 713.09 | 709.42 | 1.15 | 1.87 | ν13 | 712.48 | 721.03 | 1.59 | 1.27 |
| δ14 | 593.63 | 559.53 | 63.46 | 87.80 | δ14 | 581.04 | 527.81 | 63.02 | 125.47 |
| δ15 | 549.78 | 530.22 | 52.26 | 221.73 | δ15 | 560.13 | 570.64 | 29.25 | 44.09 |
| δ16 | 546.45 | 551.07 | 8.10 | 35.75 | δ16 | 546.83 | 543.27 | 19.46 | 55.58 |
| δ17 | 367.51 | 367.51 | 11.86 | 11.86 | δ17 | 377.64 | 377.64 | 10.84 | 10.84 |
| δ18 | 300.51 | 300.51 | 7.19 | 7.19 | δ18 | 313.36 | 313.36 | 7.59 | 7.59 |
| δ19 | 190.47 | 190.47 | 106.52 | 106.52 | δ19 | 222.78 | 222.78 | 13.77 | 13.77 |
| δ20 | 110.23 | 110.23 | 0.07 | 0.07 | δ20 | 168.22 | 168.22 | 162.53 | 162.53 |
| δ21 | 86.80 | 86.80 | 19.49 | 19.49 | δ21 | 101.45 | 101.45 | 1.19 | 1.19 |
| δ22 | 56.68 | 56.68 | 1.83 | 1.83 | δ22 | 73.88 | 73.88 | 0.41 | 0.41 |
| δ23 | 28.48 | 28.48 | 0.34 | 0.34 | δ23 | 43.74 | 43.74 | 5.11 | 5.11 |
| δ24 | 26.18 | 26.18 | 0.91 | 0.91 | δ24 | 27.85 | 27.85 | 1.43 | 1.43 |

| | **fp 23-26a** | | | | | **fp 23-26b** | | | |
|---|---|---|---|---|---|---|---|---|---|
| Mode | $E_{harm}$ | $E_{anharm}$ | $I_{harm}$ | $I_{anharm}$ | Mode | $E_{harm}$ | $E_{anharm}$ | $I_{harm}$ | $I_{anharm}$ |
| ν1 | 3539.09 | 3367.04 | 71.96 | 45.74 | ν1 | 3555.95 | 3372.78 | 70.77 | 52.29 |
| ν2 | 3496.89 | 3371.17 | 1.74 | 4.57 | ν2 | 3497.43 | 3367.07 | 1.58 | 3.99 |
| ν3 | 3389.23 | 3243.00 | 129.50 | 95.71 | ν3 | 3388.92 | 3268.98 | 148.76 | 112.76 |
| ν4 | 2677.78 | 2542.29 | 0.15 | 0.07 | ν4 | 2650.65 | 2536.20 | 35.81 | 27.96 |
| ν5 | 2208.25 | 2126.29 | 785.44 | 693.26 | ν5 | 2209.71 | 2159.09 | 979.05 | 837.99 |
| ν6 | 2058.20 | 2037.13 | 1.40 | 2.67 | ν6 | 2057.98 | 2031.24 | 0.67 | 1.13 |
| ν7 | 1352.26 | 1365.15 | 0.35 | 0.43 | ν7 | 1353.12 | 1301.85 | 0.15 | 0.09 |
| δ8 | 1024.18 | 996.39 | 17.41 | 13.65 | δ8 | 1049.53 | 1025.92 | 5.08 | 9.23 |
| δ9 | 960.02 | 945.14 | 235.73 | 198.22 | δ9 | 942.67 | 888.73 | 284.70 | 238.49 |
| δ10 | 794.36 | 781.13 | 118.81 | 67.85 | δ10 | 786.62 | 779.64 | 129.84 | 154.67 |
| δ11 | 774.38 | 743.87 | 82.49 | 92.66 | δ11 | 782.08 | 754.95 | 84.80 | 87.59 |
| δ12 | 680.93 | 651.61 | 17.43 | 11.16 | δ12 | 683.31 | 703.65 | 27.81 | 32.18 |
| δ13 | 673.49 | 702.70 | 1.38 | 23.76 | δ13 | 671.21 | 654.26 | 6.46 | 35.51 |
| δ14 | 662.19 | 632.82 | 6.03 | 30.51 | δ14 | 666.30 | 661.17 | 4.55 | 24.01 |
| δ15 | 626.83 | 596.42 | 57.87 | 28.13 | δ15 | 604.39 | 627.09 | 72.42 | 0.13 |
| ν16 | 593.50 | 579.33 | 6.15 | 16.36 | ν16 | 588.08 | 523.83 | 9.01 | 66.58 |
| δ17 | 464.86 | 457.66 | 40.38 | 26.87 | δ17 | 466.17 | 464.80 | 41.56 | 100.54 |
| δ18 | 232.82 | 232.82 | 14.50 | 14.50 | δ18 | 251.69 | 251.69 | 9.72 | 9.72 |
| δ19 | 177.12 | 177.12 | 4.08 | 4.08 | δ19 | 190.69 | 190.69 | 11.42 | 11.42 |
| δ20 | 133.03 | 133.03 | 0.13 | 0.13 | δ20 | 132.41 | 132.41 | 0.99 | 0.99 |
| δ21 | 88.49 | 88.49 | 5.40 | 5.40 | δ21 | 75.08 | 75.08 | 4.63 | 4.63 |
| δ22 | 68.10 | 68.10 | 0.42 | 0.42 | δ22 | 69.94 | 69.94 | 0.50 | 0.50 |
| δ23 | 49.98 | 49.98 | 0.45 | 0.45 | δ23 | 47.53 | 47.53 | 3.41 | 3.41 |
| δ24 | 43.48 | 43.48 | 0.09 | 0.09 | δ24 | 45.19 | 45.19 | 1.52 | 1.52 |

| | **fp 36-39 A** | | | | | **fp 36-39 B** | | | |
|---|---|---|---|---|---|---|---|---|---|
| Mode | $E_{harm}$ | $E_{anharm}$ | $I_{harm}$ | $I_{anharm}$ | Mode | $E_{harm}$ | $E_{anharm}$ | $I_{harm}$ | $I_{anharm}$ |
| ν1 | 3473.85 | 3332.12 | 33.55 | 34.93 | ν1 | 3506.23 | 3355.20 | 930.56 | 634.78 |

| | | | | | | | | | |
|---|---|---|---|---|---|---|---|---|---|
| ν2 | 3319.98 | 3177.83 | 2430.65 | 1194.20 | ν2 | 3477.65 | 3269.34 | 15.25 | 131.40 |
| ν3 | 3248.31 | 3099.64 | 4.33 | 2.32 | ν3 | 3246.83 | 3105.33 | 2.58 | 1.13 |
| ν4 | 3159.77 | 2982.66 | 23.85 | 42.13 | ν4 | 3156.74 | 3027.19 | 12.74 | 9.42 |
| ν5 | 2113.48 | 2036.98 | 469.94 | 405.62 | ν5 | 2122.20 | 2031.80 | 418.78 | 270.69 |
| ν6 | 2073.11 | 1996.03 | 685.72 | 627.72 | ν6 | 2053.05 | 1965.29 | 590.35 | 546.67 |
| δ7 | 1435.32 | 1411.10 | 3.42 | 8.90 | δ7 | 1437.77 | 1422.83 | 7.10 | 1.68 |
| ν8 | 1173.88 | 1125.00 | 63.62 | 26.06 | ν8 | 1162.55 | 1148.39 | 12.18 | 13.10 |
| δ9 | 1085.39 | 1068.54 | 145.40 | 232.50 | δ9 | 1004.36 | 995.95 | 0.03 | 0.18 |
| δ10 | 992.75 | 1055.94 | 0.11 | 0.20 | δ10 | 991.10 | 975.86 | 170.45 | 120.95 |
| δ11 | 898.30 | 826.50 | 54.91 | 82.65 | δ11 | 896.74 | 865.94 | 43.14 | 3.74 |
| ν12 | 869.76 | 847.90 | 12.58 | 12.68 | ν12 | 868.15 | 855.78 | 9.36 | 4.37 |
| δ13 | 798.30 | 786.56 | 326.01 | 259.22 | δ13 | 727.76 | 717.09 | 279.41 | 204.54 |
| δ14 | 755.72 | 805.73 | 64.45 | 70.67 | δ14 | 696.88 | 685.54 | 126.94 | 99.33 |
| δ15 | 622.62 | 579.27 | 74.68 | 21.27 | δ15 | 514.08 | 502.62 | 25.00 | 19.02 |
| δ16 | 517.72 | 498.38 | 17.52 | 0.76 | δ16 | 507.89 | 495.12 | 9.53 | 5.40 |
| δ17 | 480.80 | 447.24 | 10.79 | 76.07 | δ17 | 488.70 | 483.37 | 8.96 | 12.66 |
| δ18 | 463.63 | 468.82 | 12.96 | 196.37 | δ18 | 420.17 | 428.34 | 0.70 | 0.23 |
| δ19 | 417.63 | 435.63 | 3.72 | 0.76 | δ19 | 386.15 | 335.68 | 36.42 | 32.42 |
| δ20 | 166.79 | 166.79 | 21.08 | 21.08 | δ20 | 166.50 | 200.78 | 49.20 | 52.78 |
| δ21 | 129.60 | 129.60 | 11.67 | 11.67 | δ21 | 120.75 | 116.90 | 5.41 | 4.68 |
| δ22 | 90.97 | 90.97 | 2.45 | 2.45 | δ22 | 96.85 | 93.21 | 1.87 | 1.76 |
| δ23 | 20.89 | 20.89 | 0.42 | 0.42 | δ23 | 58.35 | 49.79 | 1.91 | 2.65 |
| δ24 | 5.01 | 5.01 | 3.86 | 3.86 | δ24 | 28.15 | 37.72 | 0.72 | 0.94 |

| | **fp 36-39 C** | | | | | **fp 39-49 D** | | | |
|---|---|---|---|---|---|---|---|---|---|
| Mode | $E_{harm}$ | $E_{anharm}$ | $I_{harm}$ | $I_{anharm}$ | Mode | $E_{harm}$ | $E_{anharm}$ | $I_{harm}$ | $I_{anharm}$ |
| ν1 | 3641.64 | 3485.67 | 219.25 | 142.60 | ν1 | 3472.71 | 3306.32 | 32.26 | 26.05 |
| ν2 | 3406.15 | 3260.10 | 343.79 | 228.46 | ν2 | 3248.25 | 3105.42 | 3.99 | 2.20 |
| ν3 | 3248.00 | 3114.80 | 0.64 | 0.25 | ν3 | 3231.40 | 3092.70 | 1743.12 | 1271.40 |
| ν4 | 3160.77 | 3051.98 | 8.82 | 7.56 | ν4 | 3159.79 | 3039.17 | 28.53 | 143.67 |
| ν5 | 2117.29 | 2026.96 | 463.93 | 354.44 | ν5 | 2123.31 | 2045.95 | 242.40 | 196.93 |
| ν6 | 2012.96 | 1926.19 | 827.92 | 761.01 | ν6 | 2110.17 | 2032.33 | 387.31 | 251.19 |
| δ7 | 1438.77 | 1420.42 | 5.45 | 2.28 | δ7 | 1435.40 | 1385.78 | 3.60 | 10.22 |
| ν8 | 1165.44 | 1170.40 | 18.91 | 4.11 | ν8 | 1169.62 | 1162.07 | 50.71 | 20.18 |
| δ9 | 1066.67 | 1050.57 | 183.31 | 215.66 | δ9 | 1073.32 | 1056.77 | 164.52 | 208.24 |
| δ10 | 1001.68 | 992.93 | 0.01 | 8.04 | δ10 | 993.61 | 973.65 | 0.07 | 0.25 |
| δ11 | 936.23 | 907.98 | 43.30 | 49.83 | δ11 | 899.77 | 874.32 | 52.76 | 71.06 |
| ν12 | 892.56 | 863.54 | 5.36 | 192.23 | δ12 | 751.22 | 737.81 | 64.06 | 177.38 |
| δ13 | 722.43 | 573.60 | 424.07 | 343.52 | ν13 | 748.64 | 771.91 | 129.52 | 12.96 |
| δ14 | 704.86 | 690.18 | 56.44 | 8.75 | δ14 | 663.68 | 591.99 | 33.40 | 35.83 |
| δ15 | 514.25 | 521.96 | 10.50 | 118.79 | δ15 | 632.48 | 564.32 | 43.41 | 28.19 |
| δ16 | 494.59 | 473.31 | 2.56 | 205.73 | δ16 | 482.59 | 490.63 | 6.64 | 26.09 |
| δ17 | 471.40 | 458.12 | 53.18 | 1254.02 | δ17 | 433.90 | 438.85 | 3.69 | 4.40 |
| δ18 | 424.24 | 438.38 | 0.68 | 205.09 | δ18 | 433.87 | 439.93 | 2.55 | 2.74 |
| δ19 | 201.90 | 201.90 | 102.03 | 102.03 | δ19 | 419.68 | 410.72 | 1.31 | 0.01 |
| δ20 | 108.73 | 108.73 | 0.34 | 0.34 | δ20 | 140.18 | 140.18 | 23.07 | 23.07 |

| | | | | | | | | | |
|---|---|---|---|---|---|---|---|---|---|
| δ21 | 93.05 | 93.05 | 17.12 | 17.12 | δ21 | 132.61 | 132.61 | 8.57 | 8.57 |
| δ22 | 75.86 | 75.86 | 0.75 | 0.75 | δ22 | 77.79 | 77.79 | 1.06 | 1.06 |
| δ23 | 12.82 | 12.82 | 8.30 | 8.30 | δ23 | 49.64 | 49.64 | 0.45 | 0.45 |
| δ24 | 9.58 | 9.58 | 1.22 | 1.22 | δ24 | 19.68 | 19.68 | 0.17 | 0.17 |

| | **fp 39-49 E** | | | | | **fp 39-49 F** | | | |
|---|---|---|---|---|---|---|---|---|---|
| Mode | $E_{harm}$ | $E_{anharm}$ | $I_{harm}$ | $I_{anharm}$ | Mode | $E_{harm}$ | $E_{anharm}$ | $I_{harm}$ | $I_{anharm}$ |
| ν1 | 3474.58 | 3325.32 | 336.67 | 197.36 | ν1 | 3473.56 | 3324.97 | 349.85 | 289.42 |
| ν2 | 3422.06 | 3275.24 | 229.49 | 198.77 | ν2 | 3399.78 | 3254.44 | 276.82 | 216.96 |
| ν3 | 3246.46 | 3108.35 | 0.65 | 0.10 | ν3 | 3248.08 | 3105.12 | 0.84 | 0.33 |
| ν4 | 3158.20 | 3044.05 | 8.31 | 5.59 | ν4 | 3160.76 | 3030.00 | 11.17 | 8.98 |
| ν5 | 2148.46 | 2069.65 | 318.41 | 367.56 | ν5 | 2152.81 | 2073.99 | 251.11 | 185.64 |
| ν6 | 2118.69 | 2041.49 | 252.39 | 157.77 | ν6 | 2117.04 | 2039.79 | 515.73 | 459.76 |
| δ7 | 1438.65 | 1414.24 | 6.35 | 2.78 | δ7 | 1438.56 | 1441.25 | 5.16 | 0.06 |
| ν8 | 1163.04 | 1159.45 | 10.98 | 5.38 | ν8 | 1167.00 | 1147.14 | 24.80 | 0.05 |
| δ9 | 1017.41 | 1001.46 | 160.82 | 147.19 | δ9 | 1063.68 | 1047.21 | 367.14 | 372.14 |
| δ10 | 1004.78 | 973.05 | 0.08 | 0.61 | δ10 | 1000.94 | 993.97 | 0.01 | 1.10 |
| δ11 | 915.09 | 881.64 | 33.84 | 39.51 | δ11 | 923.91 | 885.15 | 32.81 | 42.02 |
| ν12 | 748.02 | 725.88 | 36.41 | 67.06 | ν12 | 752.07 | 724.85 | 48.16 | 74.09 |
| δ13 | 691.31 | 624.84 | 98.37 | 140.09 | δ13 | 709.47 | 696.10 | 82.18 | 159.36 |
| δ14 | 516.75 | 489.57 | 12.85 | 20.94 | δ14 | 510.24 | 500.60 | 20.85 | 36.91 |
| δ15 | 440.99 | 429.49 | 10.59 | 37.97 | δ15 | 444.15 | 451.35 | 24.58 | 1.36 |
| δ16 | 429.88 | 392.20 | 0.39 | 24.01 | δ16 | 430.44 | 405.87 | 0.01 | 11.42 |
| δ17 | 422.73 | 419.47 | 0.15 | 2.74 | δ17 | 422.89 | 432.10 | 0.08 | 3.29 |
| δ18 | 407.36 | 466.78 | 50.25 | 73.49 | δ18 | 417.41 | 394.39 | 47.67 | 86.50 |
| δ19 | 399.93 | 399.93 | 73.13 | 73.13 | δ19 | 404.28 | 417.49 | 59.46 | 61.30 |
| δ20 | 158.95 | 158.95 | 23.06 | 23.06 | δ20 | 140.52 | 140.52 | 19.46 | 19.46 |
| δ21 | 106.39 | 106.39 | 0.78 | 0.78 | δ21 | 89.37 | 89.37 | 2.16 | 2.16 |
| δ22 | 84.20 | 84.20 | 2.46 | 2.46 | δ22 | 81.87 | 81.87 | 0.59 | 0.59 |
| δ23 | 56.30 | 56.30 | 12.70 | 12.70 | δ23 | 34.83 | 34.83 | 16.30 | 16.30 |
| δ24 | 33.28 | 33.28 | 0.39 | 0.39 | δ24 | 16.21 | 16.21 | 2.59 | 2.59 |

| | **fp 27-28a** | | | | | **fp 27-28b** | | | |
|---|---|---|---|---|---|---|---|---|---|
| Mode (quanta) | $E_{harm}$ | $E_{anharm}$ | $I_{harm}$ | $I_{anharm}$ | Mode (quanta) | $E_{harm}$ | $E_{anharm}$ | $I_{harm}$ | $I_{anharm}$ |
| 1(1) | 3604.68 | 3450.91 | 90.34 | 59.25 | 1(1) | 3600.47 | 3427.90 | 96.47 | 38.22 |
| 2(1) | 3347.93 | 3172.23 | 457.94 | 171.74 | 2(1) | 3335.85 | 3252.70 | 174.64 | 9.06 |
| 3(1) | 3332.70 | 3204.56 | 14.32 | 111.81 | 3(1) | 3331.57 | 3070.06 | 319.78 | 376.71 |
| 4(1) | 3276.18 | 3142.71 | 42.99 | 39.26 | 4(1) | 3278.02 | 3149.88 | 41.36 | 65.40 |
| 5(1) | 2335.92 | 2250.56 | 122.66 | 110.36 | 5(1) | 2336.42 | 2304.23 | 124.43 | 111.88 |
| 6(1) | 1752.60 | 1713.94 | 12.26 | 10.20 | 6(1) | 1752.63 | 1714.08 | 11.34 | 9.61 |
| 7(1) | 1617.05 | 1568.77 | 30.93 | 35.54 | 7(1) | 1622.64 | 1573.69 | 39.39 | 41.00 |
| 8(1) | 1219.42 | 1173.98 | 8.78 | 13.42 | 8(1) | 1222.15 | 1180.30 | 7.14 | 11.12 |
| 9(1) | 1104.61 | 1105.81 | 9.13 | 13.23 | 9(1) | 1102.38 | 1103.62 | 8.61 | 12.39 |
| 10(1) | 952.49 | 927.39 | 38.17 | 33.91 | 10(1) | 951.57 | 923.15 | 39.75 | 33.71 |
| 11(1) | 918.83 | 894.82 | 2.52 | 0.93 | 11(1) | 918.51 | 891.81 | 1.98 | 1.44 |
| 12(1) | 775.20 | 767.63 | 2.52 | 3.17 | 12(1) | 773.75 | 757.30 | 1.53 | 3.82 |
| 13(1) | 653.44 | 643.33 | 14.40 | 4.21 | 13(1) | 666.85 | 531.04 | 128.36 | 60.20 |

| | | | | | | | | | |
|---|---|---|---|---|---|---|---|---|---|
| 14(1) | 648.28 | 638.08 | 133.82 | 67.97 | 14(1) | 652.69 | 634.67 | 11.81 | 11.08 |
| 15(1) | 617.00 | 607.12 | 91.81 | 55.24 | 15(1) | 615.90 | 585.59 | 81.37 | 61.46 |
| 16(1) | 529.56 | 523.11 | 5.91 | 10.50 | 16(1) | 526.23 | 521.43 | 4.88 | 5.79 |
| 17(1) | 471.22 | 455.85 | 0.49 | 0.38 | 17(1) | 472.90 | 456.27 | 0.55 | 0.37 |
| 18(1) | 431.21 | 427.72 | 0.85 | 1.19 | 18(1) | 436.01 | 433.08 | 0.98 | 1.68 |
| 19(1) | 284.22 | 237.90 | 58.32 | 60.71 | 19(1) | 281.53 | 219.31 | 59.25 | 57.07 |
| 20(1) | 146.82 | 138.80 | 20.50 | 65.98 | 20(1) | 151.53 | 139.77 | 29.24 | 31.04 |
| 21(1) | 143.87 | 122.43 | 11.59 | 0.81 | 21(1) | 139.29 | 86.01 | 7.38 | 11.91 |
| 22(1) | 78.50 | 73.13 | 10.31 | 2.78 | 22(1) | 78.17 | 68.01 | 12.51 | 4.73 |
| 23(1) | 59.29 | 33.58 | 0.92 | 2.28 | 23(1) | 54.23 | 13.48 | 2.13 | 1.51 |
| 24(1) | 51.10 | 87.38 | 10.81 | 16.95 | 24(1) | 49.74 | 29.63 | 13.08 | 11.58 |

| | | **fp 27-30a** | | | | | **fp 27-30b** | | |
|---|---|---|---|---|---|---|---|---|---|
| Mode (quanta) | $E_{harm}$ | $E_{anharm}$ | $I_{harm}$ | $I_{anharm}$ | Mode (quanta) | $E_{harm}$ | $E_{anharm}$ | $I_{harm}$ | $I_{anharm}$ |
| 1(1) | 3615.70 | 3462.82 | 113.27 | 63.97 | 1(1) | 3624.53 | 3456.42 | 122.26 | 65.09 |
| 2(1) | 3495.88 | 3348.41 | 105.03 | 110.45 | 2(1) | 3502.73 | 3343.39 | 87.97 | 96.40 |
| 3(1) | 3461.58 | 3312.54 | 96.63 | 84.32 | 3(1) | 3462.18 | 3330.65 | 93.74 | 81.55 |
| 4(1) | 2597.99 | 2462.19 | 73.16 | 73.45 | 4(1) | 2609.65 | 2465.58 | 59.49 | 62.76 |
| 5(1) | 2340.95 | 2253.91 | 111.17 | 105.31 | 5(1) | 2343.26 | 2309.33 | 114.42 | 109.89 |
| 6(1) | 2132.36 | 2097.71 | 30.70 | 31.88 | 6(1) | 2131.58 | 2091.98 | 33.97 | 34.84 |
| 7(1) | 1617.15 | 1573.70 | 39.70 | 38.54 | 7(1) | 1617.28 | 1570.13 | 42.71 | 42.59 |
| 8(1) | 1206.34 | 1173.70 | 4.19 | 3.48 | 8(1) | 1200.44 | 1167.73 | 4.00 | 3.06 |
| 9(1) | 1103.04 | 1103.45 | 6.64 | 7.92 | 9(1) | 1103.96 | 1100.20 | 6.49 | 9.26 |
| 10(1) | 1002.59 | 969.74 | 4.66 | 3.79 | 10(1) | 998.79 | 970.90 | 4.62 | 3.46 |
| 11(1) | 715.34 | 674.02 | 30.42 | 33.12 | 11(1) | 714.81 | 710.75 | 10.13 | 1.26 |
| 12(1) | 713.01 | 710.92 | 13.05 | 0.30 | 12(1) | 712.72 | 671.43 | 33.42 | 36.75 |
| 13(1) | 609.39 | 599.95 | 149.73 | 106.22 | 13(1) | 571.59 | 374.81 | 99.56 | 62.95 |
| 14(1) | 563.36 | 533.79 | 52.70 | 61.62 | 14(1) | 556.95 | 533.73 | 78.36 | 223.69 |
| 15(1) | 514.10 | 511.45 | 27.54 | 10.32 | 15(1) | 502.80 | 483.40 | 61.07 | 57.93 |
| 16(1) | 421.79 | 421.37 | 0.09 | 5.80 | 16(1) | 425.57 | 423.28 | 0.31 | 5.54 |
| 17(1) | 386.91 | 380.74 | 12.59 | 8.37 | 17(1) | 386.83 | 379.36 | 14.30 | 10.31 |
| 18(1) | 323.03 | 317.31 | 12.74 | 10.50 | 18(1) | 322.38 | 327.13 | 13.19 | 10.40 |
| 19(1) | 264.55 | 202.78 | 22.65 | 23.58 | 19(1) | 250.95 | 156.05 | 10.13 | 50.44 |
| 20(1) | 219.84 | 184.25 | 39.38 | 33.09 | 20(1) | 190.45 | 214.59 | 48.51 | 14.87 |
| 21(1) | 119.02 | 130.58 | 8.68 | 13.29 | 21(1) | 118.04 | 133.54 | 11.91 | 25.77 |
| 22(1) | 103.48 | 94.65 | 10.79 | 1.38 | 22(1) | 101.85 | 107.67 | 10.65 | 0.27 |
| 23(1) | 61.74 | 65.62 | 10.21 | 16.40 | 23(1) | 59.56 | 63.66 | 10.53 | 5.14 |
| 24(1) | 41.70 | 31.18 | 11.21 | 8.65 | 24(1) | 33.91 | 55.01 | 9.18 | 4.98 |

| | | **fp 27-30c** | | | | | **fp 28-29** | | |
|---|---|---|---|---|---|---|---|---|---|
| Mode (quanta) | $E_{harm}$ | $E_{anharm}$ | $I_{harm}$ | $I_{anharm}$ | Mode (quanta) | $E_{harm}$ | $E_{anharm}$ | $I_{harm}$ | $I_{anharm}$ |
| 1(1) | 3640.58 | 3469.38 | 71.24 | 23.23 | 1(1) | 3583.07 | 3410.26 | 69.84 | 62.26 |
| 2(1) | 3548.08 | 3393.51 | 52.80 | 74.01 | 2(1) | 3406.59 | 3260.17 | 473.26 | 235.17 |
| 3(1) | 3387.60 | 3262.71 | 445.95 | 343.88 | 3(1) | 3330.63 | 3193.32 | 8.97 | 138.37 |
| 4(1) | 2637.89 | 2538.48 | 1.46 | 6.11 | 4(1) | 3274.18 | 3147.07 | 38.46 | 31.56 |
| 5(1) | 2358.15 | 2322.37 | 166.08 | 159.49 | 5(1) | 2211.80 | 2130.12 | 625.50 | 367.65 |

| Mode (quanta) | $E_{harm}$ | $E_{anharm}$ | $I_{harm}$ | $I_{anharm}$ | Mode (quanta) | $E_{harm}$ | $E_{anharm}$ | $I_{harm}$ | $I_{anharm}$ |
|---|---|---|---|---|---|---|---|---|---|
| 6(1) | 2138.71 | 2113.40 | 0.34 | 0.62 | 6(1) | 1752.63 | 1715.13 | 12.65 | 10.72 |
| 7(1) | 1627.20 | 1587.28 | 41.14 | 116.63 | 7(1) | 1284.30 | 1275.44 | 1.72 | 1.58 |
| 8(1) | 1187.96 | 1146.78 | 0.11 | 1.01 | 8(1) | 974.08 | 958.41 | 226.23 | 261.53 |
| 9(1) | 1103.61 | 1093.95 | 10.01 | 43.35 | 9(1) | 955.66 | 911.11 | 156.82 | 34.64 |
| 10(1) | 974.94 | 950.87 | 15.68 | 42.87 | 10(1) | 950.47 | 939.47 | 24.90 | 169.26 |
| 11(1) | 812.94 | 746.20 | 26.93 | 21.63 | 11(1) | 919.32 | 891.15 | 3.23 | 2.37 |
| 12(1) | 713.87 | 705.97 | 0.84 | 4.11 | 12(1) | 792.24 | 733.46 | 55.49 | 59.46 |
| 13(1) | 699.67 | 647.12 | 46.49 | 38.05 | 13(1) | 776.61 | 774.67 | 2.38 | 3.94 |
| 14(1) | 550.84 | 429.70 | 110.38 | 1943.46 | 14(1) | 656.35 | 638.84 | 9.10 | 9.58 |
| 15(1) | 483.75 | 377.03 | 127.97 | 2854.61 | 15(1) | 615.80 | 605.93 | 93.97 | 81.90 |
| 16(1) | 414.76 | 417.76 | 0.15 | 5.34 | 16(1) | 565.45 | 562.08 | 19.76 | 32.38 |
| 17(1) | 369.25 | 369.25 | 6.73 | 6.73 | 17(1) | 550.75 | 540.30 | 22.32 | 13.47 |
| 18(1) | 308.63 | 308.63 | 6.85 | 6.85 | 18(1) | 469.02 | 450.86 | 0.33 | 0.19 |
| 19(1) | 73.30 | 73.30 | 2.72 | 2.72 | 19(1) | 290.57 | 260.63 | 131.09 | 114.97 |
| 20(1) | 68.94 | 68.94 | 0.57 | 0.57 | 20(1) | 128.72 | 95.55 | 1.22 | 0.20 |
| 21(1) | 61.60 | 61.60 | 1.65 | 1.65 | 21(1) | 113.79 | 139.37 | 21.06 | 0.88 |
| 22(1) | 23.47 | 23.47 | 25.86 | 25.86 | 22(1) | 70.21 | 75.67 | 4.28 | 14.58 |
| 23(1) | 14.45 | 14.45 | 18.81 | 18.81 | 23(1) | 64.48 | 46.20 | 4.46 | 2.54 |
| 24(1) | 11.63 | 11.63 | 13.78 | 13.78 | 24(1) | 42.11 | 31.08 | 1.62 | 1.87 |

| | **fp 29-30a** | | | | | **fp 29-30b** | | | |
|---|---|---|---|---|---|---|---|---|---|
| Mode (quanta) | $E_{harm}$ | $E_{anharm}$ | $I_{harm}$ | $I_{anharm}$ | Mode (quanta) | $E_{harm}$ | $E_{anharm}$ | $I_{harm}$ | $I_{anharm}$ |
| 1(1) | 3589.41 | 3418.75 | 61.62 | 136.61 | 1(1) | 3584.50 | 3416.02 | 62.34 | 113.82 |
| 2(1) | 3567.56 | 3414.80 | 184.12 | 100.94 | 2(1) | 3552.80 | 3378.30 | 212.26 | 142.75 |
| 3(1) | 3465.51 | 3316.92 | 82.79 | 80.73 | 3(1) | 3463.87 | 3334.87 | 94.43 | 84.36 |
| 4(1) | 2639.63 | 2514.36 | 0.63 | 0.39 | 4(1) | 2633.70 | 2510.86 | 16.78 | 11.69 |
| 5(1) | 2215.89 | 2134.02 | 599.35 | 340.04 | 5(1) | 2216.55 | 2209.33 | 570.17 | 286.91 |
| 6(1) | 2151.11 | 2113.18 | 8.33 | 10.51 | 6(1) | 2141.04 | 2110.41 | 9.78 | 10.82 |
| 7(1) | 1286.40 | 1275.57 | 0.35 | 0.08 | 7(1) | 1286.58 | 1276.29 | 0.36 | 0.31 |
| 8(1) | 975.98 | 953.34 | 18.28 | 11.07 | 8(1) | 995.29 | 969.48 | 14.31 | 15.61 |
| 9(1) | 936.02 | 910.69 | 204.48 | 296.64 | 9(1) | 944.17 | 897.70 | 231.14 | 338.70 |
| 10(1) | 925.82 | 877.37 | 159.45 | 49.80 | 10(1) | 936.79 | 887.74 | 135.55 | 115.51 |
| 11(1) | 743.50 | 971.23 | 75.29 | 24.82 | 11(1) | 742.81 | 771.08 | 78.93 | 90.06 |
| 12(1) | 734.74 | 412.35 | 24.50 | 93.21 | 12(1) | 722.03 | 587.49 | 16.80 | 32.10 |
| 13(1) | 713.09 | 709.42 | 1.15 | 1.87 | 13(1) | 712.48 | 721.03 | 1.59 | 1.27 |
| 14(1) | 593.63 | 559.53 | 63.46 | 87.80 | 14(1) | 581.04 | 527.81 | 63.02 | 125.47 |
| 15(1) | 549.78 | 530.22 | 52.26 | 221.73 | 15(1) | 560.13 | 570.64 | 29.25 | 44.09 |
| 16(1) | 546.45 | 551.07 | 8.10 | 35.75 | 16(1) | 546.83 | 543.27 | 19.46 | 55.58 |
| 17(1) | 367.51 | 367.51 | 11.86 | 11.86 | 17(1) | 377.64 | 377.64 | 10.84 | 10.84 |
| 18(1) | 300.51 | 300.51 | 7.19 | 7.19 | 18(1) | 313.36 | 313.36 | 7.59 | 7.59 |
| 19(1) | 190.47 | 190.47 | 106.52 | 106.52 | 19(1) | 222.78 | 222.78 | 13.77 | 13.77 |
| 20(1) | 110.23 | 110.23 | 0.07 | 0.07 | 20(1) | 168.22 | 168.22 | 162.53 | 162.53 |
| 21(1) | 86.80 | 86.80 | 19.49 | 19.49 | 21(1) | 101.45 | 101.45 | 1.19 | 1.19 |
| 22(1) | 56.68 | 56.68 | 1.83 | 1.83 | 22(1) | 73.88 | 73.88 | 0.41 | 0.41 |
| 23(1) | 28.48 | 28.48 | 0.34 | 0.34 | 23(1) | 43.74 | 43.74 | 5.11 | 5.11 |
| 24(1) | 26.18 | 26.18 | 0.91 | 0.91 | 24(1) | 27.85 | 27.85 | 1.43 | 1.43 |

| | fp 23-26a | | | | | fp 23-26b | | | |
|---|---|---|---|---|---|---|---|---|---|
| Mode (quanta) | $E_{harm}$ | $E_{anharm}$ | $I_{harm}$ | $I_{anharm}$ | Mode (quanta) | $E_{harm}$ | $E_{anharm}$ | $I_{harm}$ | $I_{anharm}$ |
| 1(1) | 3539.09 | 3367.04 | 71.96 | 45.74 | 1(1) | 3555.95 | 3372.78 | 70.77 | 52.29 |
| 2(1) | 3496.89 | 3371.17 | 1.74 | 4.57 | 2(1) | 3497.43 | 3367.07 | 1.58 | 3.99 |
| 3(1) | 3389.23 | 3243.00 | 129.50 | 95.71 | 3(1) | 3388.92 | 3268.98 | 148.76 | 112.76 |
| 4(1) | 2677.78 | 2542.29 | 0.15 | 0.07 | 4(1) | 2650.65 | 2536.20 | 35.81 | 27.96 |
| 5(1) | 2208.25 | 2126.29 | 785.44 | 693.26 | 5(1) | 2209.71 | 2159.09 | 979.05 | 837.99 |
| 6(1) | 2058.20 | 2037.13 | 1.40 | 2.67 | 6(1) | 2057.98 | 2031.24 | 0.67 | 1.13 |
| 7(1) | 1352.26 | 1365.15 | 0.35 | 0.43 | 7(1) | 1353.12 | 1301.85 | 0.15 | 0.09 |
| 8(1) | 1024.18 | 996.39 | 17.41 | 13.65 | 8(1) | 1049.53 | 1025.92 | 5.08 | 9.23 |
| 9(1) | 960.02 | 945.14 | 235.73 | 198.22 | 9(1) | 942.67 | 888.73 | 284.70 | 238.49 |
| 10(1) | 794.36 | 781.13 | 118.81 | 67.85 | 10(1) | 786.62 | 779.64 | 129.84 | 154.67 |
| 11(1) | 774.38 | 743.87 | 82.49 | 92.66 | 11(1) | 782.08 | 754.95 | 84.80 | 87.59 |
| 12(1) | 680.93 | 651.61 | 17.43 | 11.16 | 12(1) | 683.31 | 703.65 | 27.81 | 32.18 |
| 13(1) | 673.49 | 702.70 | 1.38 | 23.76 | 13(1) | 671.21 | 654.26 | 6.46 | 35.51 |
| 14(1) | 662.19 | 632.82 | 6.03 | 30.51 | 14(1) | 666.30 | 661.17 | 4.55 | 24.01 |
| 15(1) | 626.83 | 596.42 | 57.87 | 28.13 | 15(1) | 604.39 | 627.09 | 72.42 | 0.13 |
| 16(1) | 593.50 | 579.33 | 6.15 | 16.36 | 16(1) | 588.08 | 523.83 | 9.01 | 66.58 |
| 17(1) | 464.86 | 457.66 | 40.38 | 26.87 | 17(1) | 466.17 | 464.80 | 41.56 | 100.54 |
| 18(1) | 232.82 | 232.82 | 14.50 | 14.50 | 18(1) | 251.69 | 251.69 | 9.72 | 9.72 |
| 19(1) | 177.12 | 177.12 | 4.08 | 4.08 | 19(1) | 190.69 | 190.69 | 11.42 | 11.42 |
| 20(1) | 133.03 | 133.03 | 0.13 | 0.13 | 20(1) | 132.41 | 132.41 | 0.99 | 0.99 |
| 21(1) | 88.49 | 88.49 | 5.40 | 5.40 | 21(1) | 75.08 | 75.08 | 4.63 | 4.63 |
| 22(1) | 68.10 | 68.10 | 0.42 | 0.42 | 22(1) | 69.94 | 69.94 | 0.50 | 0.50 |
| 23(1) | 49.98 | 49.98 | 0.45 | 0.45 | 23(1) | 47.53 | 47.53 | 3.41 | 3.41 |
| 24(1) | 43.48 | 43.48 | 0.09 | 0.09 | 24(1) | 45.19 | 45.19 | 1.52 | 1.52 |

| | fp 36-39 A | | | | | fp 36-39 B | | | |
|---|---|---|---|---|---|---|---|---|---|
| Mode (quanta) | $E_{harm}$ | $E_{anharm}$ | $I_{harm}$ | $I_{anharm}$ | Mode (quanta) | $E_{harm}$ | $E_{anharm}$ | $I_{harm}$ | $I_{anharm}$ |
| 1(1) | 3473.85 | 3332.12 | 33.55 | 34.93 | 1(1) | 3506.23 | 3355.20 | 930.56 | 634.78 |
| 2(1) | 3319.98 | 3177.83 | 2430.65 | 1194.20 | 2(1) | 3477.65 | 3269.34 | 15.25 | 131.40 |
| 3(1) | 3248.31 | 3099.64 | 4.33 | 2.32 | 3(1) | 3246.83 | 3105.33 | 2.58 | 1.13 |
| 4(1) | 3159.77 | 2982.66 | 23.85 | 42.13 | 4(1) | 3156.74 | 3027.19 | 12.74 | 9.42 |
| 5(1) | 2113.48 | 2036.98 | 469.94 | 405.62 | 5(1) | 2122.20 | 2031.80 | 418.78 | 270.69 |
| 6(1) | 2073.11 | 1996.03 | 685.72 | 627.72 | 6(1) | 2053.05 | 1965.29 | 590.35 | 546.67 |
| 7(1) | 1435.32 | 1411.10 | 3.42 | 8.90 | 7(1) | 1437.77 | 1422.83 | 7.10 | 1.68 |
| 8(1) | 1173.88 | 1125.00 | 63.62 | 26.06 | 8(1) | 1162.55 | 1148.39 | 12.18 | 13.10 |
| 9(1) | 1085.39 | 1068.54 | 145.40 | 232.50 | 9(1) | 1004.36 | 995.95 | 0.03 | 0.18 |
| 10(1) | 992.75 | 1055.94 | 0.11 | 0.20 | 10(1) | 991.10 | 975.86 | 170.45 | 120.95 |
| 11(1) | 898.30 | 826.50 | 54.91 | 82.65 | 11(1) | 896.74 | 865.94 | 43.14 | 3.74 |
| 12(1) | 869.76 | 847.90 | 12.58 | 12.68 | 12(1) | 868.15 | 855.78 | 9.36 | 4.37 |
| 13(1) | 798.30 | 786.56 | 326.01 | 259.22 | 13(1) | 727.76 | 717.09 | 279.41 | 204.54 |
| 14(1) | 755.72 | 805.73 | 64.45 | 70.67 | 14(1) | 696.88 | 685.54 | 126.94 | 99.33 |
| 15(1) | 622.62 | 579.27 | 74.68 | 21.27 | 15(1) | 514.08 | 502.62 | 25.00 | 19.02 |
| 16(1) | 517.72 | 498.38 | 17.52 | 0.76 | 16(1) | 507.89 | 495.12 | 9.53 | 5.40 |

| | | | | | | | | | |
|---|---|---|---|---|---|---|---|---|---|
| 17(1) | 480.80 | 447.24 | 10.79 | 76.07 | 17(1) | 488.70 | 483.37 | 8.96 | 12.66 |
| 18(1) | 463.63 | 468.82 | 12.96 | 196.37 | 18(1) | 420.17 | 428.34 | 0.70 | 0.23 |
| 19(1) | 417.63 | 435.63 | 3.72 | 0.76 | 19(1) | 386.15 | 335.68 | 36.42 | 32.42 |
| 20(1) | 166.79 | 166.79 | 21.08 | 21.08 | 20(1) | 166.50 | 200.78 | 49.20 | 52.78 |
| 21(1) | 129.60 | 129.60 | 11.67 | 11.67 | 21(1) | 120.75 | 116.90 | 5.41 | 4.68 |
| 22(1) | 90.97 | 90.97 | 2.45 | 2.45 | 22(1) | 96.85 | 93.21 | 1.87 | 1.76 |
| 23(1) | 20.89 | 20.89 | 0.42 | 0.42 | 23(1) | 58.35 | 49.79 | 1.91 | 2.65 |
| 24(1) | 5.01 | 5.01 | 3.86 | 3.86 | 24(1) | 28.15 | 37.72 | 0.72 | 0.94 |

| | **fp 36-39 C** | | | | | **fp 39-49 D** | | | |
|---|---|---|---|---|---|---|---|---|---|
| Mode (quanta) | $E_{harm}$ | $E_{anharm}$ | $I_{harm}$ | $I_{anharm}$ | Mode (quanta) | $E_{harm}$ | $E_{anharm}$ | $I_{harm}$ | $I_{anharm}$ |
| 1(1) | 3641.64 | 3485.67 | 219.25 | 142.60 | 1(1) | 3472.71 | 3306.32 | 32.26 | 26.05 |
| 2(1) | 3406.15 | 3260.10 | 343.79 | 228.46 | 2(1) | 3248.25 | 3105.42 | 3.99 | 2.20 |
| 3(1) | 3248.00 | 3114.80 | 0.64 | 0.25 | 3(1) | 3231.40 | 3092.70 | 1743.12 | 1271.40 |
| 4(1) | 3160.77 | 3051.98 | 8.82 | 7.56 | 4(1) | 3159.79 | 3039.17 | 28.53 | 143.67 |
| 5(1) | 2117.29 | 2026.96 | 463.93 | 354.44 | 5(1) | 2123.31 | 2045.95 | 242.40 | 196.93 |
| 6(1) | 2012.96 | 1926.19 | 827.92 | 761.01 | 6(1) | 2110.17 | 2032.33 | 387.31 | 251.19 |
| 7(1) | 1438.77 | 1420.42 | 5.45 | 2.28 | 7(1) | 1435.40 | 1385.78 | 3.60 | 10.22 |
| 8(1) | 1165.44 | 1170.40 | 18.91 | 4.11 | 8(1) | 1169.62 | 1162.07 | 50.71 | 20.18 |
| 9(1) | 1066.67 | 1050.57 | 183.31 | 215.66 | 9(1) | 1073.32 | 1056.77 | 164.52 | 208.24 |
| 10(1) | 1001.68 | 992.93 | 0.01 | 8.04 | 10(1) | 993.61 | 973.65 | 0.07 | 0.25 |
| 11(1) | 936.23 | 907.98 | 43.30 | 49.83 | 11(1) | 899.77 | 874.32 | 52.76 | 71.06 |
| 12(1) | 892.56 | 863.54 | 5.36 | 192.23 | 12(1) | 751.22 | 737.81 | 64.06 | 177.38 |
| 13(1) | 722.43 | 573.60 | 424.07 | 343.52 | 13(1) | 748.64 | 771.91 | 129.52 | 12.96 |
| 14(1) | 704.86 | 690.18 | 56.44 | 8.75 | 14(1) | 663.68 | 591.99 | 33.40 | 35.83 |
| 15(1) | 514.25 | 521.96 | 10.50 | 118.79 | 15(1) | 632.48 | 564.32 | 43.41 | 28.19 |
| 16(1) | 494.59 | 473.31 | 2.56 | 205.73 | 16(1) | 482.59 | 490.63 | 6.64 | 26.09 |
| 17(1) | 471.40 | 458.12 | 53.18 | 1254.02 | 17(1) | 433.90 | 438.85 | 3.69 | 4.40 |
| 18(1) | 424.24 | 438.38 | 0.68 | 205.09 | 18(1) | 433.87 | 439.93 | 2.55 | 2.74 |
| 19(1) | 201.90 | 201.90 | 102.03 | 102.03 | 19(1) | 419.68 | 410.72 | 1.31 | 0.01 |
| 20(1) | 108.73 | 108.73 | 0.34 | 0.34 | 20(1) | 140.18 | 140.18 | 23.07 | 23.07 |
| 21(1) | 93.05 | 93.05 | 17.12 | 17.12 | 21(1) | 132.61 | 132.61 | 8.57 | 8.57 |
| 22(1) | 75.86 | 75.86 | 0.75 | 0.75 | 22(1) | 77.79 | 77.79 | 1.06 | 1.06 |
| 23(1) | 12.82 | 12.82 | 8.30 | 8.30 | 23(1) | 49.64 | 49.64 | 0.45 | 0.45 |
| 24(1) | 9.58 | 9.58 | 1.22 | 1.22 | 24(1) | 19.68 | 19.68 | 0.17 | 0.17 |

| | **fp 39-49 E** | | | | | **fp 39-49 F** | | | |
|---|---|---|---|---|---|---|---|---|---|
| Mode (quanta) | $E_{harm}$ | $E_{anharm}$ | $I_{harm}$ | $I_{anharm}$ | Mode (quanta) | $E_{harm}$ | $E_{anharm}$ | $I_{harm}$ | $I_{anharm}$ |
| 1(1) | 3474.58 | 3325.32 | 336.67 | 197.36 | 1(1) | 3473.56 | 3324.97 | 349.85 | 289.42 |
| 2(1) | 3422.06 | 3275.24 | 229.49 | 198.77 | 2(1) | 3399.78 | 3254.44 | 276.82 | 216.96 |
| 3(1) | 3246.46 | 3108.35 | 0.65 | 0.10 | 3(1) | 3248.08 | 3105.12 | 0.84 | 0.33 |
| 4(1) | 3158.20 | 3044.05 | 8.31 | 5.59 | 4(1) | 3160.76 | 3030.00 | 11.17 | 8.98 |
| 5(1) | 2148.46 | 2069.65 | 318.41 | 367.56 | 5(1) | 2152.81 | 2073.99 | 251.11 | 185.64 |
| 6(1) | 2118.69 | 2041.49 | 252.39 | 157.77 | 6(1) | 2117.04 | 2039.79 | 515.73 | 459.76 |
| 7(1) | 1438.65 | 1414.24 | 6.35 | 2.78 | 7(1) | 1438.56 | 1441.25 | 5.16 | 0.06 |
| 8(1) | 1163.04 | 1159.45 | 10.98 | 5.38 | 8(1) | 1167.00 | 1147.14 | 24.80 | 0.05 |

| | | | | | | | | | |
|---|---|---|---|---|---|---|---|---|---|
| 9(1) | 1017.41 | 1001.46 | 160.82 | 147.19 | 9(1) | 1063.68 | 1047.21 | 367.14 | 372.14 |
| 10(1) | 1004.78 | 973.05 | 0.08 | 0.61 | 10(1) | 1000.94 | 993.97 | 0.01 | 1.10 |
| 11(1) | 915.09 | 881.64 | 33.84 | 39.51 | 11(1) | 923.91 | 885.15 | 32.81 | 42.02 |
| 12(1) | 748.02 | 725.88 | 36.41 | 67.06 | 12(1) | 752.07 | 724.85 | 48.16 | 74.09 |
| 13(1) | 691.31 | 624.84 | 98.37 | 140.09 | 13(1) | 709.47 | 696.10 | 82.18 | 159.36 |
| 14(1) | 516.75 | 489.57 | 12.85 | 20.94 | 14(1) | 510.24 | 500.60 | 20.85 | 36.91 |
| 15(1) | 440.99 | 429.49 | 10.59 | 37.97 | 15(1) | 444.15 | 451.35 | 24.58 | 1.36 |
| 16(1) | 429.88 | 392.20 | 0.39 | 24.01 | 16(1) | 430.44 | 405.87 | 0.01 | 11.42 |
| 17(1) | 422.73 | 419.47 | 0.15 | 2.74 | 17(1) | 422.89 | 432.10 | 0.08 | 3.29 |
| 18(1) | 407.36 | 466.78 | 50.25 | 73.49 | 18(1) | 417.41 | 394.39 | 47.67 | 86.50 |
| 19(1) | 399.93 | 399.93 | 73.13 | 73.13 | 19(1) | 404.28 | 417.49 | 59.46 | 61.30 |
| 20(1) | 158.95 | 158.95 | 23.06 | 23.06 | 20(1) | 140.52 | 140.52 | 19.46 | 19.46 |
| 21(1) | 106.39 | 106.39 | 0.78 | 0.78 | 21(1) | 89.37 | 89.37 | 2.16 | 2.16 |
| 22(1) | 84.20 | 84.20 | 2.46 | 2.46 | 22(1) | 81.87 | 81.87 | 0.59 | 0.59 |
| 23(1) | 56.30 | 56.30 | 12.70 | 12.70 | 23(1) | 34.83 | 34.83 | 16.30 | 16.30 |
| 24(1) | 33.28 | 33.28 | 0.39 | 0.39 | 24(1) | 16.21 | 16.21 | 2.59 | 2.59 |

**Table S3.** Optimi□ed structures (B3LYP-D3/6-311++G(3df,3pd)) and geometrical parameters of the identified ACA photoproduct molecules. Bond lengths (R) are given in Ångstroms, valence (A) and dihedral (D) angles are given in degrees.

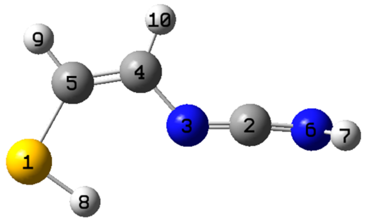


**fp1a**

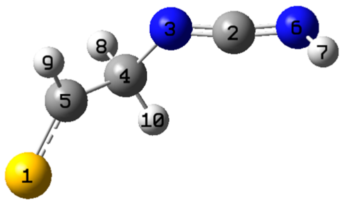


**fp2a**

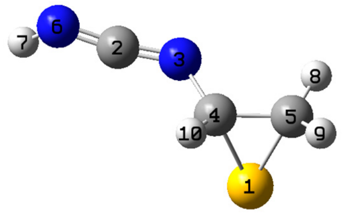


**fp3**

| Name | Definition | Value | Name | Definition | Value | Name | Definition | Value |
|---|---|---|---|---|---|---|---|---|
| R1 | R(2,3) | 1.22 | R1 | R(2,3) | 1.21 | R1 | R(2,3) | 1.22 |
| R2 | R(2,6) | 1.22 | R2 | R(2,6) | 1.22 | R2 | R(2,6) | 1.22 |
| R3 | R(3,4) | 1.39 | R3 | R(3,4) | 1.45 | R3 | R(3,4) | 1.42 |
| R4 | R(4,10) | 1.08 | R4 | R(5,9) | 1.09 | R4 | R(4,10) | 1.08 |
| R5 | R(4,5) | 1.34 | R5 | R(5,1) | 1.61 | R5 | R(4,1) | 1.84 |
| R6 | R(5,9) | 1.08 | R6 | R(5,4) | 1.50 | R6 | R(4,5) | 1.47 |
| R7 | R(5,1) | 1.75 | R7 | R(6,7) | 1.01 | R7 | R(1,5) | 1.83 |
| R8 | R(1,8) | 1.34 | R8 | R(4,8) | 1.10 | R8 | R(6,7) | 1.01 |
| R9 | R(6,7) | 1.01 | R9 | R(4,10) | 1.09 | R9 | R(5,9) | 1.08 |
| A1 | A(2,3,4) | 130.9 | A1 | A(2,3,4) | 127.6 | R10 | R(5,8) | 1.08 |
| A2 | A(3,4,10) | 118.0 | A2 | A(9,5,1) | 121.1 | A1 | A(2,3,4) | 127.7 |
| A3 | A(3,4,5) | 122.2 | A3 | A(9,5,4) | 113.7 | A2 | A(3,4,10) | 115.1 |
| A4 | A(10,4,5) | 119.8 | A4 | A(1,5,4) | 125.2 | A3 | A(3,4,1) | 118.2 |
| A5 | A(4,5,9) | 118.7 | A5 | A(2,6,7) | 119.5 | A4 | A(3,4,5) | 117.8 |
| A6 | A(4,5,1) | 128.0 | A6 | A(3,4,5) | 110.8 | A5 | A(10,4,1) | 113.2 |
| A7 | A(9,5,1) | 113.3 | A7 | A(3,4,8) | 107.8 | A6 | A(10,4,5) | 117.7 |
| A8 | A(5,1,8) | 96.6 | A8 | A(3,4,10) | 113.2 | A7 | A(2,6,7) | 120.3 |
| A9 | A(2,6,7) | 120.5 | A9 | A(5,4,8) | 106.8 | A8 | A(4,5,9) | 118.3 |
| D1 | D(4,3,6,7) | 94.3 | A10 | A(5,4,10) | 110.5 | A9 | A(4,5,8) | 116.5 |
| D2 | D(2,3,4,10) | 2.1 | A11 | A(8,4,10) | 107.5 | A10 | A(1,5,9) | 115.6 |
| D3 | D(2,3,4,5) | -178.2 | D1 | D(4,3,6,7) | -93.0 | A11 | A(1,5,8) | 115.2 |
| D4 | D(3,4,5,9) | -179.7 | D2 | D(2,3,4,5) | 127.4 | A12 | A(9,5,8) | 115.5 |
| D5 | D(3,4,5,1) | 0.2 | D3 | D(2,3,4,8) | -116.0 | D1 | D(4,3,6,7) | -93.9 |
| D6 | D(10,4,5,9) | -0.1 | D4 | D(2,3,4,10) | 2.8 | D2 | D(2,3,4,10) | -31.6 |
| D7 | D(10,4,5,1) | 179.9 | D5 | D(9,5,4,3) | 32.5 | D3 | D(2,3,4,1) | 106.6 |
| D8 | D(4,5,1,8) | -0.5 | D6 | D(9,5,4,8) | -84.7 | D4 | D(2,3,4,5) | -177.5 |
| D9 | D(9,5,1,8) | 179.4 | D7 | D(9,5,4,10) | 158.7 | D5 | D(3,4,5,9) | 141.9 |
| | | | D8 | D(1,5,4,3) | -149.1 | D6 | D(3,4,5,8) | -2.7 |
| | | | D9 | D(1,5,4,8) | 93.7 | D7 | D(10,4,5,9) | -3.1 |
| | | | D10 | D(1,5,4,10) | -22.9 | D8 | D(10,4,5,8) | -147.7 |

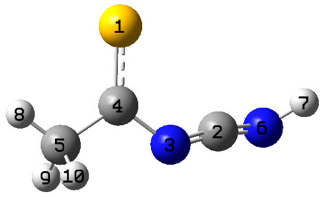


**fp4**

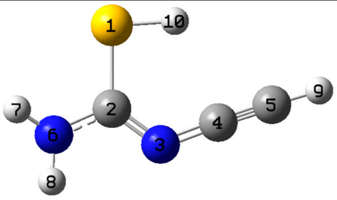


**fp14a**

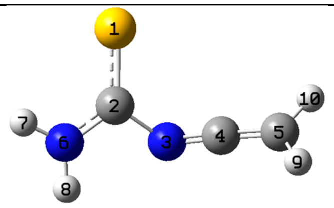


**fp15**

| | | | | | | | | |
|---|---|---|---|---|---|---|---|---|
| R1 | R(2,3) | 1.22 | R1 | R(4,3) | 1.32 | R1 | R(4,5) | 1.30 |
| R2 | R(2,6) | 1.21 | R2 | R(4,5) | 1.21 | R2 | R(4,3) | 1.22 |
| R3 | R(3,4) | 1.39 | R3 | R(2,3) | 1.29 | R3 | R(2,1) | 1.65 |
| R4 | R(6,7) | 1.01 | R4 | R(2,1) | 1.78 | R4 | R(2,6) | 1.34 |
| R5 | R(4,1) | 1.64 | R5 | R(2,6) | 1.36 | R5 | R(2,3) | 1.41 |
| R6 | R(4,5) | 1.50 | R6 | R(5,9) | 1.06 | R6 | R(6,8) | 1.01 |
| R7 | R(5,8) | 1.08 | R7 | R(1,10) | 1.35 | R7 | R(6,7) | 1.00 |
| R8 | R(5,9) | 1.09 | R8 | R(6,8) | 1.01 | R8 | R(5,10) | 1.08 |
| R9 | R(5,10) | 1.09 | R9 | R(6,7) | 1.00 | R9 | R(5,9) | 1.08 |
| A1 | A(2,3,4) | 129.3 | A1 | A(3,2,1) | 126.4 | A1 | A(1,2,6) | 124.9 |
| A2 | A(2,6,7) | 124.8 | A2 | A(3,2,6) | 119.6 | A2 | A(1,2,3) | 124.3 |
| A3 | A(3,4,1) | 124.0 | A3 | A(1,2,6) | 114.0 | A3 | A(6,2,3) | 110.7 |
| A4 | A(3,4,5) | 111.9 | A4 | A(4,3,2) | 123.1 | A4 | A(2,6,8) | 120.5 |
| A5 | A(1,4,5) | 124.1 | A5 | A(2,1,10) | 93.8 | A5 | A(2,6,7) | 119.2 |
| A6 | A(4,5,8) | 111.4 | A6 | A(2,6,8) | 116.6 | A6 | A(8,6,7) | 120.0 |
| A7 | A(4,5,9) | 109.6 | A7 | A(2,6,7) | 120.4 | A7 | A(4,5,10) | 119.8 |
| A8 | A(4,5,10) | 109.3 | A8 | A(8,6,7) | 117.7 | A8 | A(4,5,9) | 120.7 |
| A9 | A(8,5,9) | 109.9 | D1 | D(1,2,3,4) | -0.9 | A9 | A(10,5,9) | 119.5 |
| A10 | A(8,5,10) | 109.9 | D2 | D(6,2,3,4) | 177.2 | A10 | A(4,3,2) | 125.6 |
| A11 | A(9,5,10) | 106.7 | D3 | D(3,2,1,10) | -3.0 | D1 | D(4,5,10,9) | 178.4 |
| D1 | D(4,3,6,7) | 78.2 | D4 | D(6,2,1,10) | 178.8 | D2 | D(10,5,3,2) | -85.0 |
| D2 | D(2,3,4,1) | -21.2 | D5 | D(3,2,6,8) | 9.6 | D3 | D(9,5,3,2) | 95.6 |
| D3 | D(2,3,4,5) | 160.7 | D6 | D(3,2,6,7) | 163.3 | D4 | D(1,2,6,8) | 174.4 |
| D4 | D(3,4,5,8) | 180.0 | D7 | D(1,2,6,8) | -172.1 | D5 | D(1,2,6,7) | 0.5 |
| D5 | D(3,4,5,9) | -58.1 | D8 | D(1,2,6,7) | -18.4 | D6 | D(3,2,6,8) | -2.6 |
| D6 | D(3,4,5,10) | 58.4 | | | | D7 | D(3,2,6,7) | -176.5 |
| D7 | D(1,4,5,8) | 1.9 | | | | D8 | D(1,2,3,4) | 46.2 |
| D8 | D(1,4,5,9) | 123.8 | | | | D9 | D(6,2,3,4) | -136.8 |
| D9 | D(1,4,5,10) | -119.7 | | | | | | |

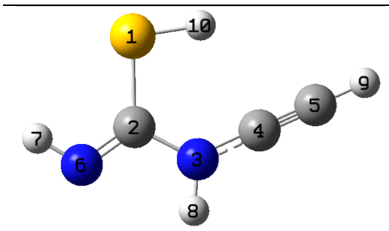


**fp17a**

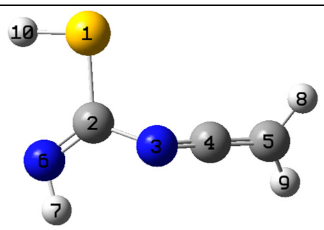


**fp18a**

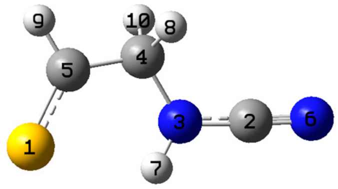


**fp33a**

| | | | | | | | | |
|---|---|---|---|---|---|---|---|---|
| R1 | R(4,5) | 1.20 | R1 | R(4,5) | 1.30 | R1 | R(2,6) | 1.16 |
| R2 | R(4,3) | 1.34 | R2 | R(4,3) | 1.22 | R2 | R(2,3) | 1.33 |
| R3 | R(2,6) | 1.27 | R3 | R(2,6) | 1.26 | R3 | R(5,9) | 1.09 |
| R4 | R(2,3) | 1.40 | R4 | R(2,3) | 1.42 | R4 | R(1,5) | 1.61 |
| R5 | R(2,1) | 1.78 | R5 | R(2,1) | 1.78 | R5 | R(4,5) | 1.49 |
| R6 | R(5,9) | 1.06 | R6 | R(6,7) | 1.02 | R6 | R(4,10) | 1.10 |
| R7 | R(6,7) | 1.01 | R7 | R(5,8) | 1.08 | R7 | R(4,8) | 1.10 |
| R8 | R(3,8) | 1.01 | R8 | R(5,9) | 1.08 | R8 | R(3,4) | 1.45 |
| R9 | R(1,10) | 1.34 | R9 | R(1,10) | 1.34 | R9 | R(3,7) | 1.01 |
| A1 | A(6,2,3) | 117.4 | A1 | A(6,2,3) | 124.9 | A1 | A(9,5,1) | 120.5 |

| | | | | | | | | |
|---|---|---|---|---|---|---|---|---|
| A2 | A(6,2,1) | 124.4 | A2 | A(6,2,1) | 123.0 | A2 | A(9,5,4) | 112.7 |
| A3 | A(3,2,1) | 118.2 | A3 | A(3,2,1) | 112.0 | A3 | A(1,5,4) | 126.8 |
| A4 | A(2,6,7) | 113.6 | A4 | A(2,6,7) | 111.6 | A4 | A(5,4,10) | 108.0 |
| A5 | A(4,3,2) | 128.1 | A5 | A(4,5,8) | 120.2 | A5 | A(5,4,8) | 107.1 |
| A6 | A(4,3,8) | 119.0 | A6 | A(4,5,9) | 120.4 | A6 | A(5,4,3) | 113.3 |
| A7 | A(2,3,8) | 112.9 | A7 | A(8,5,9) | 119.4 | A7 | A(10,4,8) | 105.9 |
| A8 | A(2,1,10) | 97.8 | A8 | A(4,3,2) | 126.4 | A8 | A(10,4,3) | 110.4 |
| D1 | D(3,2,6,7) | -180.0 | A9 | A(2,1,10) | 94.3 | A9 | A(8,4,3) | 111.7 |
| D2 | D(1,2,6,7) | 0.0 | D1 | D(4,5,8,9) | -178.9 | A10 | A(2,3,4) | 120.5 |
| D3 | D(6,2,3,4) | -180.0 | D2 | D(8,5,3,2) | 91.6 | A11 | A(2,3,7) | 118.6 |
| D4 | D(6,2,3,8) | 0.0 | D3 | D(9,5,3,2) | -89.7 | A12 | A(4,3,7) | 115.7 |
| D5 | D(1,2,3,4) | 0.0 | D4 | D(3,2,6,7) | -1.7 | D1 | D(9,5,4,10) | -53.7 |
| D6 | D(1,2,3,8) | -180.0 | D5 | D(1,2,6,7) | -177.8 | D2 | D(9,5,4,8) | 60.0 |
| D7 | D(6,2,1,10) | 180.0 | D6 | D(6,2,3,4) | 111.4 | D3 | D(9,5,4,3) | -176.3 |
| D8 | D(3,2,1,10) | 0.0 | D7 | D(1,2,3,4) | -72.1 | D4 | D(1,5,4,10) | 126.9 |
| | | | D8 | D(6,2,1,10) | 0.5 | D5 | D(1,5,4,8) | -119.4 |
| | | | D9 | D(3,2,1,10) | -176.1 | D6 | D(1,5,4,3) | 4.3 |
| | | | | | | D7 | D(5,4,3,2) | -166.0 |
| | | | | | | D8 | D(5,4,3,7) | -11.8 |
| | | | | | | D9 | D(10,4,3,2) | 72.8 |
| | | | | | | D10 | D(10,4,3,7) | -133.0 |
| | | | | | | D11 | D(8,4,3,2) | -44.8 |
| | | | | | | D12 | D(8,4,3,7) | 109.4 |

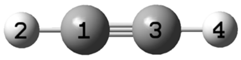


**fp23**

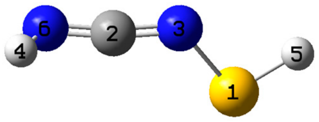


**fp26**

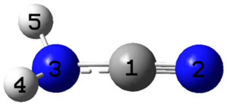


**fp27**

| | | | | | | | | |
|---|---|---|---|---|---|---|---|---|
| R1 | R(1,2) | 1.06 | R1 | R(2,6) | 1.22 | R1 | R(1,2) | 1.15 |
| R2 | R(1,3) | 1.20 | R2 | R(2,3) | 1.22 | R2 | R(1,3) | 1.34 |
| R3 | R(3,4) | 1.06 | R3 | R(4,6) | 1.01 | R3 | R(3,4) | 1.01 |
| | | | R4 | R(1,3) | 1.70 | R4 | R(3,5) | 1.01 |
| | | | R5 | R(1,5) | 1.34 | A1 | A(1,3,4) | 115.4 |
| | | | A1 | A(2,6,4) | 118.9 | A2 | A(1,3,5) | 115.4 |
| | | | A2 | A(2,3,1) | 126.2 | A3 | A(4,3,5) | 113.2 |
| | | | A3 | A(3,1,5) | 94.7 | D1 | D(1,3,5,4) | -136.1 |
| | | | D1 | D(4,6,3,1) | 94.1 | | | |
| | | | D2 | D(2,3,1,5) | -178.3 | | | |

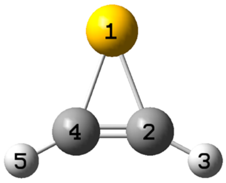


**fp28**

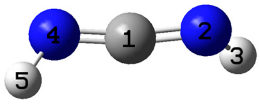


**fp29**

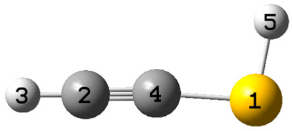


**fp30**

| | | | | | | | | |
|---|---|---|---|---|---|---|---|---|
| R1 | R(1,2) | 1.86 | R1 | R(1,2) | 1.22 | R1 | R(2,4) | 1.20 |
| R2 | R(1,4) | 1.86 | R2 | R(1,4) | 1.22 | R2 | R(1,4) | 1.69 |
| R3 | R(2,3) | 1.07 | R3 | R(2,3) | 1.01 | R3 | R(2,3) | 1.06 |

| | | |
|---|---|---|
| R4 | R(2,4) | 1.27 |
| R5 | R(4,5) | 1.07 |
| A1 | A(1,2,3) | 138.2 |
| A2 | A(3,2,4) | 151.9 |
| A3 | A(1,4,5) | 138.2 |
| A4 | A(2,4,5) | 151.9 |
| D1 | D(1,2,3,4) | -179.7 |
| D2 | D(3,2,4,5) | 0.4 |
| D3 | D(5,2,4,1) | 179.8 |

| | | |
|---|---|---|
| R4 | R(4,5) | 1.01 |
| A1 | A(1,2,3) | 120.1 |
| A2 | A(1,4,5) | 120.1 |
| D1 | D(3,2,4,5) | 93.1 |

| | | |
|---|---|---|
| R4 | R(1,5) | 1.35 |
| A1 | A(4,1,5) | 97.2 |

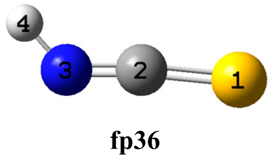

**fp36**

| | | |
|---|---|---|
| R1 | R(1,2) | 1.57 |
| R2 | R(2,3) | 1.20 |
| R3 | R(3,4) | 1.00 |
| A1 | A(4,3,2) | 132.0 |

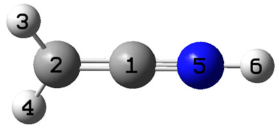

**fp39**

| | | |
|---|---|---|
| R1 | R(1,2) | 1.32 |
| R2 | R(1,5) | 1.18 |
| R3 | R(2,3) | 1.08 |
| R4 | R(2,4) | 1.08 |
| R5 | R(5,6) | 0.99 |
| A1 | A(1,2,3) | 120.0 |
| A2 | A(1,2,4) | 120.0 |
| A3 | A(3,2,4) | 120.0 |
| A4 | A(1,5,6) | 179.9 |
| D1 | D(1,2,3,4) | 179.9 |
| D2 | D(3,2,5,6) | -5.3 |
| D3 | D(4,2,5,6) | 174.7 |

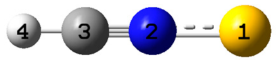

**fp49**

| | | |
|---|---|---|
| R1 | R(1,2) | 1.60 |
| R2 | R(2,3) | 1.16 |
| R3 | R(3,4) | 1.06 |

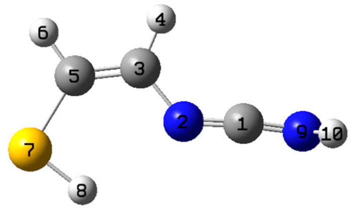

**fp1a**

| Name | Definition | Value |
|---|---|---|
| R1 | R(1,2) | 1.22 |
| R2 | R(1,9) | 1.22 |
| R3 | R(2,3) | 1.39 |
| R4 | R(3,4) | 1.08 |
| R5 | R(3,5) | 1.34 |
| R6 | R(5,6) | 1.08 |
| R7 | R(5,7) | 1.75 |
| R8 | R(7,8) | 1.34 |
| R9 | R(9,10) | 1.01 |
| A1 | A(1,2,3) | 130.9 |

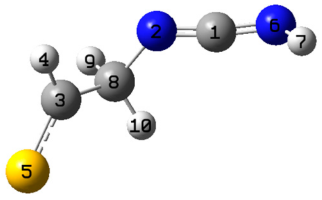

**fp2a**

| Name | Definition | Value |
|---|---|---|
| R1 | R(1,2) | 1.21 |
| R2 | R(1,6) | 1.22 |
| R3 | R(2,8) | 1.45 |
| R4 | R(3,4) | 1.09 |
| R5 | R(3,5) | 1.61 |
| R6 | R(3,8) | 1.50 |
| R7 | R(6,7) | 1.01 |
| R8 | R(8,9) | 1.10 |
| R9 | R(8,10) | 1.09 |
| A1 | A(1,2,8) | 127.6 |

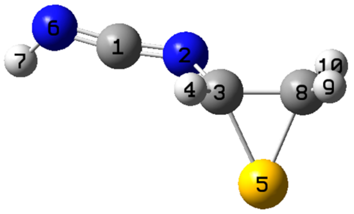

**fp3**

| Name | Definition | Value |
|---|---|---|
| R1 | R(1,2) | 1.22 |
| R2 | R(1,6) | 1.22 |
| R3 | R(2,3) | 1.42 |
| R4 | R(3,4) | 1.08 |
| R5 | R(3,5) | 1.84 |
| R6 | R(3,8) | 1.47 |
| R7 | R(5,8) | 1.83 |
| R8 | R(6,7) | 1.01 |
| R9 | R(8,9) | 1.08 |
| R10 | R(8,10) | 1.08 |

| | | | | | | | | |
|---|---|---|---|---|---|---|---|---|
| A2 | A(2,3,4) | 118.0 | A2 | A(4,3,5) | 121.1 | A1 | A(1,2,3) | 127.7 |
| A3 | A(2,3,5) | 122.2 | A3 | A(4,3,8) | 113.7 | A2 | A(2,3,4) | 115.1 |
| A4 | A(4,3,5) | 119.8 | A4 | A(5,3,8) | 125.2 | A3 | A(2,3,5) | 118.2 |
| A5 | A(3,5,6) | 118.7 | A5 | A(1,6,7) | 119.5 | A4 | A(2,3,8) | 117.8 |
| A6 | A(3,5,7) | 128.0 | A6 | A(2,8,3) | 110.8 | A5 | A(4,3,5) | 113.2 |
| A7 | A(6,5,7) | 113.3 | A7 | A(2,8,9) | 107.8 | A6 | A(4,3,8) | 117.7 |
| A8 | A(5,7,8) | 96.6 | A8 | A(2,8,10) | 113.2 | A7 | A(1,6,7) | 120.3 |
| A9 | A(1,9,10) | 120.5 | A9 | A(3,8,9) | 106.8 | A8 | A(3,8,9) | 118.3 |
| D1 | D(3,2,9,10) | 94.3 | A10 | A(3,8,10) | 110.5 | A9 | A(3,8,10) | 116.5 |
| D2 | D(1,2,3,4) | 2.1 | A11 | A(9,8,10) | 107.5 | A10 | A(5,8,9) | 115.6 |
| D3 | D(1,2,3,5) | -178.2 | D1 | D(8,2,6,7) | -93.0 | A11 | A(5,8,10) | 115.2 |
| D4 | D(2,3,5,6) | -179.7 | D2 | D(1,2,8,3) | 127.4 | A12 | A(9,8,10) | 115.5 |
| D5 | D(2,3,5,7) | 0.2 | D3 | D(1,2,8,9) | -116.0 | D1 | D(3,2,6,7) | -93.9 |
| D6 | D(4,3,5,6) | -0.1 | D4 | D(1,2,8,10) | 2.8 | D2 | D(1,2,3,4) | -31.6 |
| D7 | D(4,3,5,7) | 179.9 | D5 | D(4,3,8,2) | 32.5 | D3 | D(1,2,3,5) | 106.6 |
| D8 | D(3,5,7,8) | -0.5 | D6 | D(4,3,8,9) | -84.7 | D4 | D(1,2,3,8) | -177.5 |
| D9 | D(6,5,7,8) | 179.4 | D7 | D(4,3,8,10) | 158.7 | D5 | D(2,3,8,9) | 141.9 |
| | | | D8 | D(5,3,8,2) | -149.1 | D6 | D(2,3,8,10) | -2.7 |
| | | | D9 | D(5,3,8,9) | 93.7 | D7 | D(4,3,8,9) | -3.1 |
| | | | D10 | D(5,3,8,10) | -22.9 | D8 | D(4,3,8,10) | -147.7 |

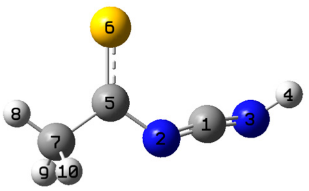

**fp4**

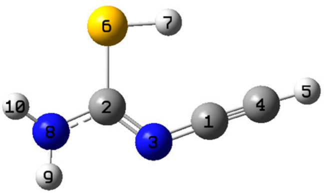

**fp14a**

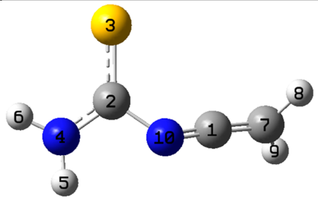

**fp15**

| | | | | | | | | |
|---|---|---|---|---|---|---|---|---|
| R1 | R(1,2) | 1.22 | R1 | R(1,3) | 1.32 | R1 | R(1,7) | 1.30 |
| R2 | R(1,3) | 1.21 | R2 | R(1,4) | 1.21 | R2 | R(1,10) | 1.22 |
| R3 | R(2,5) | 1.39 | R3 | R(2,3) | 1.29 | R3 | R(2,3) | 1.65 |
| R4 | R(3,4) | 1.01 | R4 | R(2,6) | 1.78 | R4 | R(2,4) | 1.34 |
| R5 | R(5,6) | 1.64 | R5 | R(2,8) | 1.36 | R5 | R(2,10) | 1.41 |
| R6 | R(5,7) | 1.50 | R6 | R(4,5) | 1.06 | R6 | R(4,5) | 1.01 |
| R7 | R(7,8) | 1.08 | R7 | R(6,7) | 1.35 | R7 | R(4,6) | 1.00 |
| R8 | R(7,9) | 1.09 | R8 | R(8,9) | 1.01 | R8 | R(7,8) | 1.08 |
| R9 | R(7,10) | 1.09 | R9 | R(8,10) | 1.00 | R9 | R(7,9) | 1.08 |
| A1 | A(1,2,5) | 129.3 | A1 | A(3,2,6) | 126.4 | A1 | A(3,2,4) | 124.9 |
| A2 | A(1,3,4) | 124.8 | A2 | A(3,2,8) | 119.6 | A2 | A(3,2,10) | 124.3 |
| A3 | A(2,5,6) | 124.0 | A3 | A(6,2,8) | 114.0 | A3 | A(4,2,10) | 110.7 |
| A4 | A(2,5,7) | 111.9 | A4 | A(1,3,2) | 123.1 | A4 | A(2,4,5) | 120.5 |
| A5 | A(6,5,7) | 124.1 | A5 | A(2,6,7) | 93.8 | A5 | A(2,4,6) | 119.2 |
| A6 | A(5,7,8) | 111.4 | A6 | A(2,8,9) | 116.6 | A6 | A(5,4,6) | 120.0 |
| A7 | A(5,7,9) | 109.6 | A7 | A(2,8,10) | 120.4 | A7 | A(1,7,8) | 119.8 |
| A8 | A(5,7,10) | 109.3 | A8 | A(9,8,10) | 117.7 | A8 | A(1,7,9) | 120.7 |
| A9 | A(8,7,9) | 109.9 | D1 | D(6,2,3,1) | -0.9 | A9 | A(8,7,9) | 119.5 |
| A10 | A(8,7,10) | 109.9 | D2 | D(8,2,3,1) | 177.2 | A10 | A(1,10,2) | 125.6 |
| A11 | A(9,7,10) | 106.7 | D3 | D(3,2,6,7) | -3.0 | D1 | D(1,7,8,9) | 178.4 |

| | | | | | | | | |
|---|---|---|---|---|---|---|---|---|
| D1 | D(5,2,3,4) | 78.2 | D4 | D(8,2,6,7) | 178.8 | D2 | D(8,7,10,2) | -85.0 |
| D2 | D(1,2,5,6) | -21.2 | D5 | D(3,2,8,9) | 9.6 | D3 | D(9,7,10,2) | 95.6 |
| D3 | D(1,2,5,7) | 160.7 | D6 | D(3,2,8,10) | 163.3 | D4 | D(3,2,4,5) | 174.4 |
| D4 | D(2,5,7,8) | 180.0 | D7 | D(6,2,8,9) | -172.1 | D5 | D(3,2,4,6) | 0.5 |
| D5 | D(2,5,7,9) | -58.1 | D8 | D(6,2,8,10) | -18.4 | D6 | D(10,2,4,5) | -2.6 |
| D6 | D(2,5,7,10) | 58.4 | | | | D7 | D(10,2,4,6) | -176.5 |
| D7 | D(6,5,7,8) | 1.9 | | | | D8 | D(3,2,10,1) | 46.2 |
| D8 | D(6,5,7,9) | 123.8 | | | | D9 | D(4,2,10,1) | -136.8 |
| D9 | D(6,5,7,10) | -119.7 | | | | | | |

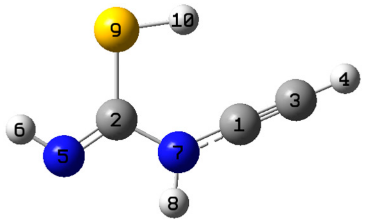


**fp17a**

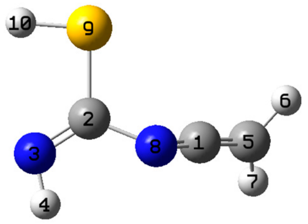


**fp18a**

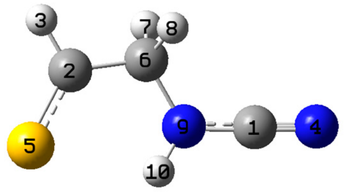


**fp33a**

| | | | | | | | | |
|---|---|---|---|---|---|---|---|---|
| R1 | R(1,3) | 1.20 | R1 | R(1,5) | 1.30 | R1 | R(1,4) | 1.16 |
| R2 | R(1,7) | 1.34 | R2 | R(1,8) | 1.22 | R2 | R(1,9) | 1.33 |
| R3 | R(2,5) | 1.27 | R3 | R(2,3) | 1.26 | R3 | R(2,3) | 1.09 |
| R4 | R(2,7) | 1.40 | R4 | R(2,8) | 1.42 | R4 | R(2,5) | 1.61 |
| R5 | R(2,9) | 1.78 | R5 | R(2,9) | 1.78 | R5 | R(2,6) | 1.49 |
| R6 | R(3,4) | 1.06 | R6 | R(3,4) | 1.02 | R6 | R(6,7) | 1.10 |
| R7 | R(5,6) | 1.01 | R7 | R(5,6) | 1.08 | R7 | R(6,8) | 1.10 |
| R8 | R(7,8) | 1.01 | R8 | R(5,7) | 1.08 | R8 | R(6,9) | 1.45 |
| R9 | R(9,10) | 1.34 | R9 | R(9,10) | 1.34 | R9 | R(9,10) | 1.01 |
| A1 | A(5,2,7) | 117.4 | A1 | A(3,2,8) | 124.9 | A1 | A(3,2,5) | 120.5 |
| A2 | A(5,2,9) | 124.4 | A2 | A(3,2,9) | 123.0 | A2 | A(3,2,6) | 112.7 |
| A3 | A(7,2,9) | 118.2 | A3 | A(8,2,9) | 112.0 | A3 | A(5,2,6) | 126.8 |
| A4 | A(2,5,6) | 113.6 | A4 | A(2,3,4) | 111.6 | A4 | A(2,6,7) | 108.0 |
| A5 | A(1,7,2) | 128.1 | A5 | A(1,5,6) | 120.2 | A5 | A(2,6,8) | 107.1 |
| A6 | A(1,7,8) | 119.0 | A6 | A(1,5,7) | 120.4 | A6 | A(2,6,9) | 113.3 |
| A7 | A(2,7,8) | 112.9 | A7 | A(6,5,7) | 119.4 | A7 | A(7,6,8) | 105.9 |
| A8 | A(2,9,10) | 97.8 | A8 | A(1,8,2) | 126.4 | A8 | A(7,6,9) | 110.4 |
| D1 | D(7,2,5,6) | -180.0 | A9 | A(2,9,10) | 94.3 | A9 | A(8,6,9) | 111.7 |
| D2 | D(9,2,5,6) | 0.0 | D1 | D(1,5,6,7) | -178.9 | A10 | A(1,9,6) | 120.5 |
| D3 | D(5,2,7,1) | -180.0 | D2 | D(6,5,8,2) | 91.6 | A11 | A(1,9,10) | 118.6 |
| D4 | D(5,2,7,8) | 0.0 | D3 | D(7,5,8,2) | -89.7 | A12 | A(6,9,10) | 115.7 |
| D5 | D(9,2,7,1) | 0.0 | D4 | D(8,2,3,4) | -1.7 | D1 | D(3,2,6,7) | -53.7 |
| D6 | D(9,2,7,8) | -180.0 | D5 | D(9,2,3,4) | -177.8 | D2 | D(3,2,6,8) | 60.0 |
| D7 | D(5,2,9,10) | 180.0 | D6 | D(3,2,8,1) | 111.4 | D3 | D(3,2,6,9) | -176.3 |
| D8 | D(7,2,9,10) | 0.0 | D7 | D(9,2,8,1) | -72.1 | D4 | D(5,2,6,7) | 126.9 |
| | | | D8 | D(3,2,9,10) | 0.5 | D5 | D(5,2,6,8) | -119.4 |
| | | | D9 | D(8,2,9,10) | -176.1 | D6 | D(5,2,6,9) | 4.3 |
| | | | | | | D7 | D(2,6,9,1) | -166.0 |
| | | | | | | D8 | D(2,6,9,10) | -11.8 |
| | | | | | | D9 | D(7,6,9,1) | 72.8 |

| | | |
|---|---|---|
| D10 | D(7,6,9,10) | -133.0 |
| D11 | D(8,6,9,1) | -44.8 |
| D12 | D(8,6,9,10) | 109.4 |

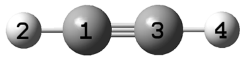

**fp23**

| | | |
|---|---|---|
| R1 | R(1,2) | 1.06 |
| R2 | R(1,3) | 1.20 |
| R3 | R(3,4) | 1.06 |

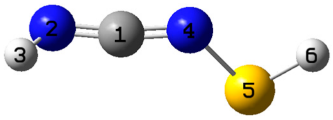

**fp26**

| | | |
|---|---|---|
| R1 | R(1,2) | 1.22 |
| R2 | R(1,4) | 1.22 |
| R3 | R(2,3) | 1.01 |
| R4 | R(4,5) | 1.70 |
| R5 | R(5,6) | 1.34 |
| A1 | A(1,2,3) | 118.9 |
| A2 | A(1,4,5) | 126.2 |
| A3 | A(4,5,6) | 94.7 |
| D1 | D(3,2,4,5) | 94.1 |
| D2 | D(1,4,5,6) | -178.3 |

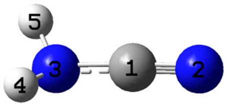

**fp27**

| | | |
|---|---|---|
| R1 | R(1,2) | 1.15 |
| R2 | R(1,3) | 1.34 |
| R3 | R(3,4) | 1.01 |
| R4 | R(3,5) | 1.01 |
| A1 | A(1,3,4) | 115.4 |
| A2 | A(1,3,5) | 115.4 |
| A3 | A(4,3,5) | 113.2 |
| D1 | D(1,3,5,4) | -136.1 |

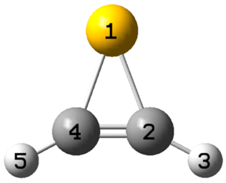

**fp28**

| | | |
|---|---|---|
| R1 | R(1,2) | 1.86 |
| R2 | R(1,4) | 1.86 |
| R3 | R(2,3) | 1.07 |
| R4 | R(2,4) | 1.27 |
| R5 | R(4,5) | 1.07 |
| A1 | A(1,2,3) | 138.2 |
| A2 | A(3,2,4) | 151.9 |
| A3 | A(1,4,5) | 138.2 |
| A4 | A(2,4,5) | 151.9 |
| D1 | D(1,2,3,4) | -179.7 |
| D2 | D(3,2,4,5) | 0.4 |
| D3 | D(5,2,4,1) | 179.8 |

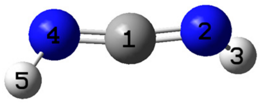

**fp29**

| | | |
|---|---|---|
| R1 | R(1,2) | 1.22 |
| R2 | R(1,4) | 1.22 |
| R3 | R(2,3) | 1.01 |
| R4 | R(4,5) | 1.01 |
| A1 | A(1,2,3) | 120.1 |
| A2 | A(1,4,5) | 120.1 |
| D1 | D(3,2,4,5) | 93.1 |

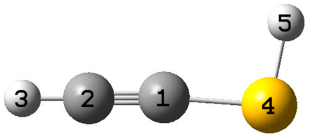

**fp30**

| | | |
|---|---|---|
| R1 | R(1,2) | 1.20 |
| R2 | R(1,4) | 1.69 |
| R3 | R(2,3) | 1.06 |
| R4 | R(4,5) | 1.35 |
| A1 | A(1,4,5) | 97.2 |

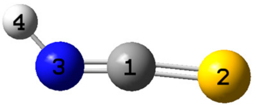

**fp36**

| | | |
|---|---|---|
| R1 | R(1,2) | 1.57 |
| R2 | R(1,3) | 1.20 |
| R3 | R(3,4) | 1.00 |
| A1 | A(1,3,4) | 132.0 |

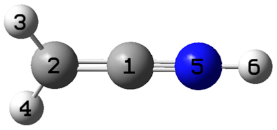

**fp39**

| | | |
|---|---|---|
| R1 | R(1,2) | 1.32 |
| R2 | R(1,5) | 1.18 |
| R3 | R(2,3) | 1.08 |
| R4 | R(2,4) | 1.08 |
| R5 | R(5,6) | 0.99 |
| A1 | A(1,2,3) | 120.0 |

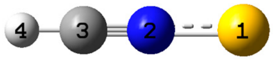

**fp49**

| | | |
|---|---|---|
| R1 | R(1,2) | 1.60 |
| R2 | R(2,3) | 1.16 |
| R3 | R(3,4) | 1.06 |

| | | |
|---|---|---|
| A2 | A(1,2,4) | 120.0 |
| A3 | A(3,2,4) | 120.0 |
| A4 | A(1,5,6) | 179.9 |
| D1 | D(1,2,3,4) | 179.9 |
| D2 | D(3,2,5,6) | -5.3 |
| D3 | D(4,2,5,6) | 174.7 |

**Table S4.** Cartesian coordinates (B3LYP-D3/6-311++G(3df,3pd)) of the identified ACA photoproduct molecules.

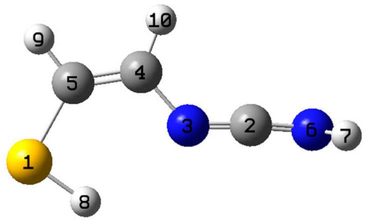

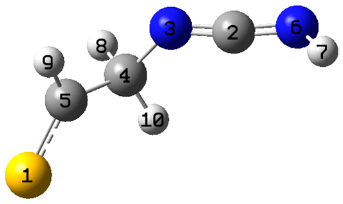

| **fp1a** | | | | | | **fp2a** | | | |
|---|---|---|---|---|---|---|---|---|---|
| Atom no. | Atom type | Coordinates (*Ångstroms*) | | | Atom no. | Atom type | Coordinates (*Ångstroms*) | | |
| | | X | Y | Z | | | X | Y | Z |
| 1 | S | 2.238747 | -0.567436 | -0.003559 | 1 | S | 2.539677 | -0.411313 | 0.029462 |
| 2 | C | -2.101298 | -0.176310 | -0.015004 | 2 | C | -2.235962 | -0.042838 | -0.092630 |
| 3 | N | -0.888551 | -0.096870 | 0.009030 | 3 | N | -1.229960 | 0.612542 | -0.262400 |
| 4 | C | -0.048251 | 1.012416 | -0.001114 | 4 | C | -0.001106 | 0.567950 | 0.513957 |
| 5 | C | 1.284259 | 0.897544 | -0.005031 | 5 | C | 1.188508 | 0.363959 | -0.382551 |
| 6 | N | -3.299641 | -0.363649 | 0.115895 | 6 | N | -3.338006 | -0.541557 | 0.079345 |
| 7 | H | -3.894416 | -0.466265 | -0.695493 | 7 | H | -3.556352 | -1.428837 | -0.353631 |
| 8 | H | 1.204270 | -1.425410 | 0.014292 | 8 | H | 0.127709 | 1.544555 | 0.995372 |
| 9 | H | 1.883305 | 1.796991 | -0.008663 | 9 | H | 1.079202 | 0.820004 | -1.366442 |
| 10 | H | -0.504029 | 1.995393 | -0.000764 | 10 | H | -0.018266 | -0.186039 | 1.302038 |

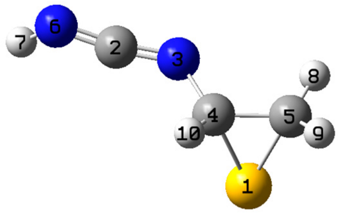

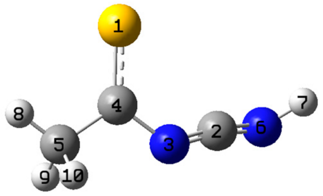

| **fp3** | | | | | | **fp4** | | | |
|---|---|---|---|---|---|---|---|---|---|
| Atom no. | Atom type | Coordinates (*Ångstroms*) | | | Atom no. | Atom type | Coordinates (*Ångstroms*) | | |
| | | X | Y | Z | | | X | Y | Z |
| 1 | S | -1.675962 | -0.801299 | 0.050458 | 1 | S | -1.029425 | 1.317363 | -0.001287 |
| 2 | C | 1.922841 | 0.041373 | 0.101133 | 2 | C | 1.667290 | -0.279490 | 0.058793 |
| 3 | N | 0.812724 | 0.460900 | 0.363883 | 3 | N | 0.575900 | -0.809190 | 0.226992 |
| 4 | C | -0.355864 | 0.387924 | -0.438094 | 4 | C | -0.694901 | -0.285308 | 0.032559 |
| 5 | C | -1.582962 | 1.023940 | 0.075732 | 5 | C | -1.729373 | -1.363170 | -0.097332 |
| 6 | N | 3.096086 | -0.205221 | -0.119432 | 6 | N | 2.793560 | 0.054209 | -0.210196 |
| 7 | H | 3.481266 | -1.107992 | 0.123185 | 7 | H | 3.337120 | 0.722028 | 0.314574 |
| 8 | H | -1.520230 | 1.468598 | 1.060045 | 8 | H | -2.718314 | -0.945402 | -0.248577 |
| 9 | H | -2.229165 | 1.534041 | -0.626359 | 9 | H | -1.471261 | -2.020740 | -0.930647 |
| 10 | H | -0.182235 | 0.416960 | -1.507989 | 10 | H | -1.721058 | -1.981021 | 0.803542 |

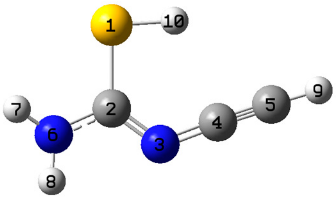

**fp14a**

| Atom no. | Atom type | Coordinates (*Ångstroms*) X | Y | Z |
|---|---|---|---|---|
| 1 | S | -0.869237 | 1.312570 | -0.004792 |
| 2 | C | -0.619781 | -0.449365 | 0.000194 |
| 3 | N | 0.514031 | -1.059459 | 0.009783 |
| 4 | C | 1.673353 | -0.426481 | -0.000106 |
| 5 | C | 2.755527 | 0.108534 | -0.001879 |
| 6 | N | -1.769285 | -1.171964 | -0.046186 |
| 7 | H | -2.646835 | -0.746733 | 0.193743 |
| 8 | H | -1.680839 | -2.167027 | 0.080620 |
| 9 | H | 3.719974 | 0.548072 | -0.007056 |
| 10 | H | 0.447683 | 1.588395 | 0.074935 |

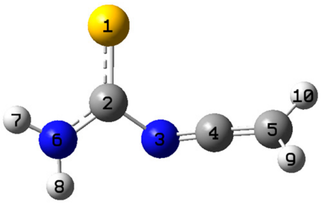

**fp15**

| Atom no. | Atom type | Coordinates (*Ångstroms*) X | Y | Z |
|---|---|---|---|---|
| 1 | S | 1.220239 | -1.220266 | 0.013782 |
| 2 | C | 0.696946 | 0.341786 | -0.057486 |
| 3 | N | -0.597181 | 0.731214 | -0.472336 |
| 4 | C | -1.643193 | 0.222055 | -0.107317 |
| 5 | C | -2.801763 | -0.265067 | 0.226107 |
| 6 | N | 1.456036 | 1.419521 | 0.207148 |
| 7 | H | 2.418825 | 1.289717 | 0.462520 |
| 8 | H | 1.094138 | 2.347350 | 0.062864 |
| 9 | H | -3.373777 | 0.173331 | 1.030262 |
| 10 | H | -3.186935 | -1.133932 | -0.287668 |

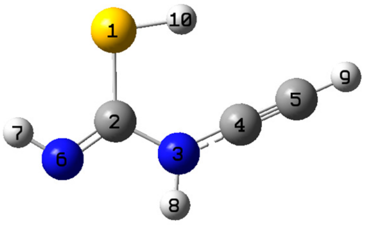

**fp17a**

| Atom no. | Atom type | Coordinates (*Ångstroms*) X | Y | Z |
|---|---|---|---|---|
| 1 | S | -0.962868 | 1.275865 | 0.000006 |
| 2 | C | -0.766739 | -0.493847 | -0.000049 |
| 3 | N | 0.528821 | -1.012834 | 0.000205 |
| 4 | C | 1.684217 | -0.342706 | 0.000120 |
| 5 | C | 2.731010 | 0.246916 | -0.000256 |
| 6 | N | -1.727213 | -1.321637 | -0.000139 |
| 7 | H | -2.640367 | -0.882702 | -0.000181 |
| 8 | H | 0.548169 | -2.023810 | 0.000247 |
| 9 | H | 3.655274 | 0.766882 | -0.000109 |
| 10 | H | 0.340624 | 1.604898 | 0.000595 |

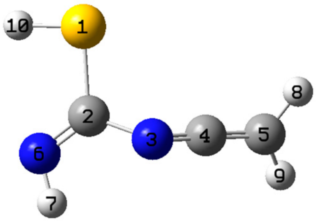

**fp18a**

| Atom no. | Atom type | Coordinates (*Ångstroms*) X | Y | Z |
|---|---|---|---|---|
| 1 | S | 1.388262 | 1.050000 | 0.012253 |
| 2 | C | 0.637007 | -0.557233 | -0.062925 |
| 3 | N | -0.651478 | -0.514983 | -0.653687 |
| 4 | C | -1.661910 | -0.056589 | -0.151906 |
| 5 | C | -2.793591 | 0.398862 | 0.306361 |
| 6 | N | 1.226435 | -1.607590 | 0.307299 |
| 7 | H | 0.650126 | -2.433071 | 0.155850 |
| 8 | H | -3.077688 | 1.425772 | 0.130917 |
| 9 | H | -3.447022 | -0.241152 | 0.880383 |
| 10 | H | 2.548657 | 0.596230 | 0.512333 |

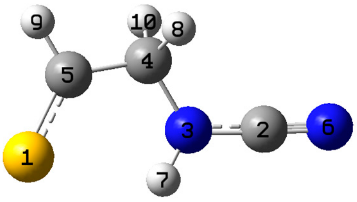

**fp33a**

| Atom no. | Atom type | Coordinates (*Ångstroms*) X | Y | Z |
|---|---|---|---|---|
| 1 | S | -2.144253 | -0.586495 | 0.032077 |
| 2 | C | 2.174904 | -0.282715 | -0.013276 |
| 3 | N | 0.862403 | -0.237322 | -0.208443 |
| 4 | C | 0.134637 | 1.002354 | -0.029875 |

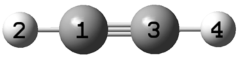

**fp23**

| Atom no. | Atom type | Coordinates (*Ångstroms*) X | Y | Z |
|---|---|---|---|---|
| 1 | C | -0.477192 | -0.144708 | 0.330545 |
| 2 | H | -1.324313 | -0.401446 | 0.917400 |
| 3 | C | 0.477196 | 0.144652 | -0.330572 |
| 4 | H | 1.324289 | 0.401780 | -0.917234 |

| | | | | |
|---|---|---|---|---|
| 5 | C | -1.345339 | 0.815129 | 0.039057 |
| 6 | N | 3.322886 | -0.293352 | 0.129446 |
| 7 | H | 0.323184 | -1.085650 | -0.080191 |
| 8 | H | 0.438092 | 1.523042 | 0.888897 |
| 9 | H | -1.885312 | 1.758415 | 0.108507 |
| 10 | H | 0.349837 | 1.694221 | -0.852906 |

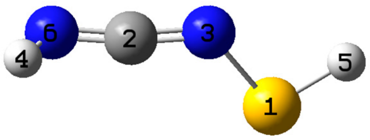


**fp26**

| Atom no. | Atom type | Coordinates (*Ångstroms)* | | |
|---|---|---|---|---|
| | | X | Y | Z |
| 1 | S | -1.460599 | -0.248608 | 0.000742 |
| 2 | C | 1.125105 | 0.140009 | -0.016767 |
| 3 | N | 0.006994 | 0.619025 | -0.009777 |
| 4 | H | 2.839152 | -0.423885 | -0.695833 |
| 5 | H | -2.235415 | 0.846241 | -0.035645 |
| 6 | N | 2.280894 | -0.231122 | 0.126949 |

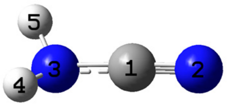


**fp27**

| Atom no. | Atom type | Coordinates (*Ångstroms)* | | |
|---|---|---|---|---|
| | | X | Y | Z |
| 1 | C | 0.221214 | -0.000056 | -0.000008 |
| 2 | N | 1.375459 | -0.000013 | 0.012475 |
| 3 | N | -1.116624 | -0.000016 | -0.081085 |
| 4 | H | -1.570112 | -0.842403 | 0.240240 |
| 5 | H | -1.569016 | 0.842942 | 0.240077 |

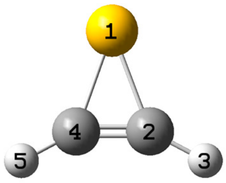


**fp28**

| Atom no. | Atom type | Coordinates (*Ångstroms)* | | |
|---|---|---|---|---|
| | | X | Y | Z |
| 1 | S | 0.000000 | 0.000000 | 0.847504 |
| 2 | C | 0.000000 | 0.635123 | -0.896269 |
| 3 | H | -0.001685 | 1.581118 | -1.402418 |
| 4 | C | 0.000000 | -0.635123 | -0.896269 |
| 5 | H | 0.001685 | -1.581118 | -1.402418 |

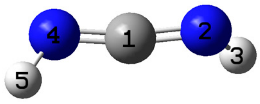


**fp29**

| Atom no. | Atom type | Coordinates (*Ångstroms)* | | |
|---|---|---|---|---|
| | | X | Y | Z |
| 1 | C | 0.000004 | -0.017364 | -0.000259 |
| 2 | N | 1.210670 | 0.077045 | 0.101204 |
| 3 | H | 1.813865 | -0.491972 | -0.475825 |
| 4 | N | -1.210758 | 0.081210 | -0.098054 |
| 5 | H | -1.813272 | -0.511629 | 0.455328 |

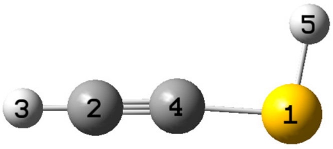


**fp30**

| Atom no. | Atom type | Coordinates (*Ångstroms)* | | |
|---|---|---|---|---|
| | | X | Y | Z |
| 1 | S | 1.037599 | -0.088020 | 0.000009 |
| 2 | C | -1.850182 | 0.004562 | 0.000022 |
| 3 | H | -2.911417 | -0.004257 | 0.000052 |
| 4 | C | -0.647456 | 0.025324 | -0.000058 |
| 5 | H | 1.295660 | 1.233269 | 0.000017 |

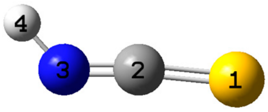


**fp36**

| Atom no. | Atom type | Coordinates (*Ångstroms)* | | |
|---|---|---|---|---|
| | | X | Y | Z |
| 1 | S | 0.028783 | -1.075074 | 0.000000 |
| 2 | C | 0.000000 | 0.493629 | 0.000000 |
| 3 | N | -0.140332 | 1.685509 | 0.000000 |
| 4 | H | 0.521804 | 2.440856 | 0.000000 |

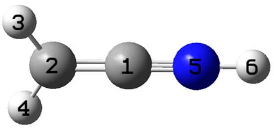


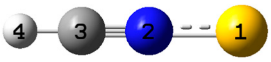

**fp39**

| Atom no. | Atom type | Coordinates (*Ångstroms)* X | Y | Z |
|---|---|---|---|---|
| 1 | C | 0.033521 | 0.000058 | 0.010878 |
| 2 | C | -1.267798 | -0.000071 | 0.010377 |
| 3 | H | -1.814508 | 0.932031 | 0.019763 |
| 4 | H | -1.815366 | -0.931527 | 0.020086 |
| 5 | N | 1.319062 | -0.000098 | -0.133295 |
| 6 | H | 1.802108 | 0.000261 | 0.765685 |

**fp49**

| Atom no. | Atom type | Coordinates (*Ångstroms)* X | Y | Z |
|---|---|---|---|---|
| 1 | S | 0 | 0 | 1.050874 |
| 2 | N | 0 | 0 | -0.545401 |
| 3 | C | 0 | 0 | -1.704946 |
| 4 | H | 0 | 0 | -2.766511 |

**Table S5**. Calculated and argon matrix experimental wavenumber shifts (in $cm^{-1}$) in HNCS-$CH_2$CNH and HCNS-$CH_2$CNH complexes with interaction energies $\Delta E^{CP}$ (in kJ $mol^{-1}$).

**fp 36-39 A**

| $\Delta\nu_{exp}$ | $\Delta\nu_{cal}$ | Assignmt |
|---|---|---|
| 0 | -1 | $\nu_{as}$CCN |
| 35 | 34 | νCN |

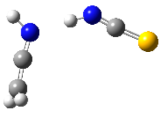

$\Delta E^{CP}$ = -31.1

**fp 36-39 B**

| $\Delta\nu_{exp}$ | $\Delta\nu_{cal}$ | Assignmt |
|---|---|---|
| - | 9 | $\nu_{as}$CCN |
| 18 | 14 | νCN |

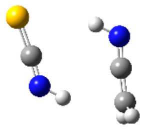

$\Delta E^{CP}$ = -21.7

**fp 36-39 C**

| $\Delta\nu_{exp}$ | $\Delta\nu_{cal}$ | Assignmt |
|---|---|---|
| - | 3 | $\nu_{as}$CCN |
| - | -26 | νCN |

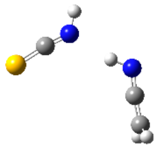

$\Delta E^{CP}$ = -12.1

**fp 39-49 D**

| $\Delta\nu_{exp}$ | $\Delta\nu_{cal}$ | Assignmt |
|---|---|---|
| -7 | -4 | $\nu_{as}$CCN |
| -8 | -13 | νCN |

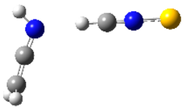

$\Delta E^{CP}$ = -25.3

**fp 39-49 E**

| $\Delta\nu_{exp}$ | $\Delta\nu_{cal}$ | Assignmt |
|---|---|---|
| 3 | 4 | $\nu_{as}$CCN |
| 12 | 12 | νCN |

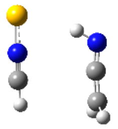

$\Delta E^{CP}$ = -18.4

**fp 39-49 F**

| $\Delta\nu_{exp}$ | $\Delta\nu_{cal}$ | Assignmt |
|---|---|---|
| 1 | 3 | $\nu_{as}$CCN |
| 16 | 16 | νCN |

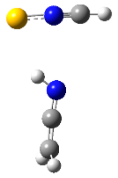

$\Delta E^{CP}$ = -17.9
